# Barriers and Facilitators of User Engagement With Digital Mental Health Interventions for People With Psychosis or Bipolar Disorder: Systematic Review and Best-Fit Framework Synthesis

**DOI:** 10.2196/65246

**Published:** 2025-01-20

**Authors:** Emily Eisner, Sophie Faulkner, Stephanie Allan, Hannah Ball, Daniela Di Basilio, Jennifer Nicholas, Aansha Priyam, Paul Wilson, Xiaolong Zhang, Sandra Bucci

**Affiliations:** 1 Division of Psychology and Mental Health University of Manchester Manchester United Kingdom; 2 Greater Manchester Mental Health NHS Foundation Trust Manchester United Kingdom; 3 School of Health and Wellbeing University of Glasgow Glasgow United Kingdom; 4 Division of Health Research Faculty of Health and Medicine Lancaster University Lancaster United Kingdom; 5 Centre for Youth Mental Health University of Melbourne Melbourne Australia; 6 Orygen Melbourne Australia; 7 Division of Population Health Health Services Research & Primary Care University of Manchester Manchester United Kingdom

**Keywords:** psychosis, bipolar, schizophrenia, smartphone, digital, wearable, mobile phone, PRISMA

## Abstract

**Background:**

Digital mental health interventions (DMHIs) to monitor and improve the health of people with psychosis or bipolar disorder show promise; however, user engagement is variable, and integrated clinical use is low.

**Objective:**

This prospectively registered systematic review examined barriers and facilitators of clinician and patient engagement with DMHIs, to inform implementation within real-world settings.

**Methods:**

A systematic search of 7 databases identified empirical studies reporting qualitative or quantitative data about factors affecting staff or patient engagement with DMHIs aiming to monitor or improve the mental or physical health of people with psychosis or bipolar disorder. The Consolidated Framework for Implementation Research was used to synthesize data on barriers and facilitators, following a best-fit framework synthesis approach.

**Results:**

The review included 175 papers (150 studies; 11,446 participants) describing randomized controlled trials; surveys; qualitative interviews; and usability, cohort, and case studies. Samples included people with schizophrenia spectrum psychosis (98/150, 65.3% of studies), bipolar disorder (62/150, 41.3% of studies), and clinicians (26/150, 17.3% of studies). Key facilitators were a strong recognition of DMHIs’ relative advantages, a clear link between intervention focus and specific patient needs, a simple, low-effort digital interface, human-supported delivery, and device provision where needed. Although staff thought patients would lose, damage, or sell devices, reviewed studies found only 11% device loss. Barriers included intervention complexity, perceived risks, user motivation, discomfort with self-reflection, digital poverty, symptoms of psychosis, poor compatibility with existing clinical workflows, staff and patient fears that DMHIs would replace traditional face-to-face care, infrastructure limitations, and limited financial support for delivery.

**Conclusions:**

Identified barriers and facilitators highlight key considerations for DMHI development and implementation. As to broader implications, sustainable business models are needed to ensure that evidence-based DMHIs are maintained and deployed.

**Trial Registration:**

PROSPERO CRD42021282871; https://www.crd.york.ac.uk/prospero/display_record.php?RecordID=282871

## Introduction

### Background

The costs and burdens of schizophrenia spectrum psychosis and bipolar disorder are huge and often neglected in research [1,2]. Digital mental health interventions (DMHIs) can help people with psychosis or bipolar disorder monitor, manage, and improve their symptoms and health [3-6]. Although there is promising evidence of feasibility, acceptability, and efficacy [7-9], and widespread expectations that DMHIs will become core to health care [10], user uptake and engagement are highly variable [11], and integrated clinical use is low [12]. The challenges of implementing DMHIs in health care are well established; attempts to implement well-evidenced DMHIs for common mental health problems in the United States and United Kingdom health services have frequently failed due to lack of patient and provider engagement and difficulty integrating DMHIs into clinical care [13]. For example, while randomized controlled trials (RCTs) of computerized cognitive behavior therapy (cCBT) for depression have reported relatively high engagement (47% of participants completing all cCBT sessions [14]), pragmatic trials delivering cCBT in real-world settings report much lower engagement (16%-18% of participants completing all sessions; most participants completing 1 session only) [15]. Implementing DMHIs for people with psychosis or bipolar disorder, within secondary care mental health services (eg, community mental health teams), presents additional challenges (eg, high staff caseloads and reactive care in response to increased risk or crisis), which have been examined in this review.

Implementation science acknowledges that intervention effectiveness cannot guarantee uptake in services; uptake depends largely on contextual barriers and facilitators [16]. Examining such contextual factors is therefore crucial to improving uptake in real-world settings, allowing tailoring of interventions to maximize engagement, and informing development and testing of implementation strategies. Barriers and facilitators of engagement with DMHIs for common mental health problems (eg, depression or anxiety) were examined relatively recently [11], but the last systematic review of factors affecting DMHI implementation in samples with psychosis or bipolar disorder was 5 years ago [17]. This review provides a timely update on this rapidly developing literature by examining barriers and facilitators of patient and staff engagement with DMHIs for this group.

Using a best-fit framework synthesis approach [18,19], the Consolidated Framework for Implementation Research (CFIR) [20] guided this analysis, enabling consideration of user engagement barriers and facilitators in the context of the complex, multilevel systems within and surrounding health care [21]. In supplementary analyses, we also examined safety reporting within reviewed studies to explore whether perceived harms may be additional barriers to DMHI uptake and to further explore recent findings on safety reporting quality [22,23].

### Objective

In summary, this review examines evidence from qualitative and quantitative studies regarding the barriers and facilitators of patient and staff engagement with DMHIs for psychosis or bipolar disorder. By synthesizing this information using CFIR, this review presents important findings about how to maximize engagement with such DMHIs, and ideal conditions for DMHI implementation in secondary mental health services.

## Methods

### Design

This systematic review follows PRISMA (Preferred Reporting Items for Systematic Reviews and Meta-Analyses; see Multimedia Appendix 1 for PRISMA checklist) guidance [24], with eligibility criteria developed according to the PICOS (participants, interventions, comparators, outcomes, study design) framework. The review was registered with PROSPERO (main review: CRD42021282871; safety review: CRD42022306123).

### Search Strategy and Selection Criteria

We conducted a PRISMA-compliant search of 7 databases using search terms relating to psychosis and bipolar disorder, DMHIs, and barriers, facilitators or implementation (see search terms and database list in Multimedia Appendix 2). Searches were restricted to peer-reviewed articles reporting on studies with human participants, published in English between January 2010 and October 2021. By restricting the search in this way, we were able to capture the period between the first studies of digital mental health tools being conducted (consistent with previous reviews [11,25]) and the COVID-19 pandemic. The search end date (1.5 years after the worldwide COVID-19 restrictions peaked [26]), allows for the lag from data collection to publication. The search retrieved very few papers reporting data gathered during or after the pandemic, suggesting this was a suitable watershed date. A future systematic review will examine studies from October 2021 onward.

Author EE combined, deduplicated, and screened the titles and abstracts of the search results; author AP independently screened a random selection (10%). Full-text of the remaining articles were independently screened against PICOS inclusion and exclusion criteria by authors EE and AP. Studies included and excluded by each researcher were compared, disagreements were resolved by consensus, and final reasons for all exclusions were recorded. Full inclusion and exclusion criteria are provided in Multimedia Appendix 2. In summary, the review included all published studies reporting qualitative or quantitative data on hypothetical or actual barriers or facilitators of patient or staff engagement with a DMHI aiming to monitor or improve the mental or physical health of adults with a bipolar or schizophrenia spectrum diagnosis using a digital method (eg, smartphone app, SMS text messaging, website, or wearable).

### Data Extraction

A data extraction form was used to extract relevant data: study metadata, design, DMHI details, and sample characteristics. Quantitative findings relating to barriers and facilitators of user engagement were extracted and imported into NVivo software (Lumivero) [27], as were the full results and discussion sections of qualitative studies. A second researcher checked all the extracted data. Study quality was assessed using the Mixed Methods Appraisal Tool [28]. In addition, from the main paper text and supplements of studies that tested the actual use of a DMHI, we extracted data on how adverse events (AEs) were monitored, classified (relatedness or severity), analyzed, reported, and discussed, and whether reporting followed the CONSORT (Consolidated Standards of Reporting Trials) harms checklist [29]. Given that the reporting of formal AE data is typically poor for DMHIs [22,23], we also extracted information about incidents that might be classifiable as AEs but were not described as such by the authors.

### Data Analysis

Study characteristics, quality checks, and AE information were tabulated and summarized descriptively. A best-fit framework synthesis approach [18,19] was used to synthesize qualitative and quantitative data from all included studies to address the following research question: What are the barriers and facilitators of service user or staff engagement with DMHIs for people with psychosis or bipolar disorder? Generally, within a best-fit framework approach, relevant published frameworks, models, or theories are systematically identified, and key elements of these are integrated into an a priori coding framework for use in the review [19]. Primary studies are then deductively coded into the a priori coding framework, with data not fitting within the framework coded inductively. In this review, we were able to use a single existing implementation framework, the CFIR, as the a priori coding framework [20,30], followed by inductive coding. CFIR was developed using a comprehensive systematic review of the implementation science literature [20,30]. It is a meta-theoretical framework incorporating constructs from a wide range of existing implementation theories into a single comprehensive framework and is used by researchers and practitioners to identify barriers and facilitators of successful implementation and tailor their strategies accordingly. Thus, it is an appropriate choice of framework to guide the systematic evaluation of potential barriers and facilitators of user engagement with DMHIs in this review. CFIR has 5 major domains as provided in Textbox 1.

**Major domains of Consolidated Framework for Implementation Research.**

- Innovation characteristics: features of the innovation or intervention being implemented, including its complexity, adaptability, design, and evidence base. For example, a smartphone app for monitoring symptoms of psychosis.
- Outer setting: the external context in which the organization is situated, including economic, political, legal, and social factors that may influence implementation. For example, a particular country or state.
- Inner setting: features of the organization in which the intervention is being implemented, including its culture, structure, resources, and readiness for change. For example, a secondary care mental health service.
- Individuals: characteristics of individuals involved in the implementation process, such as their knowledge, beliefs, and motivation. For example, patients with psychosis or bipolar disorder, and mental health staff.
- Implementation process: the implementation process itself, including planning, engaging, executing, and reflecting.

## Results

### Search Results

**Figure 1:**
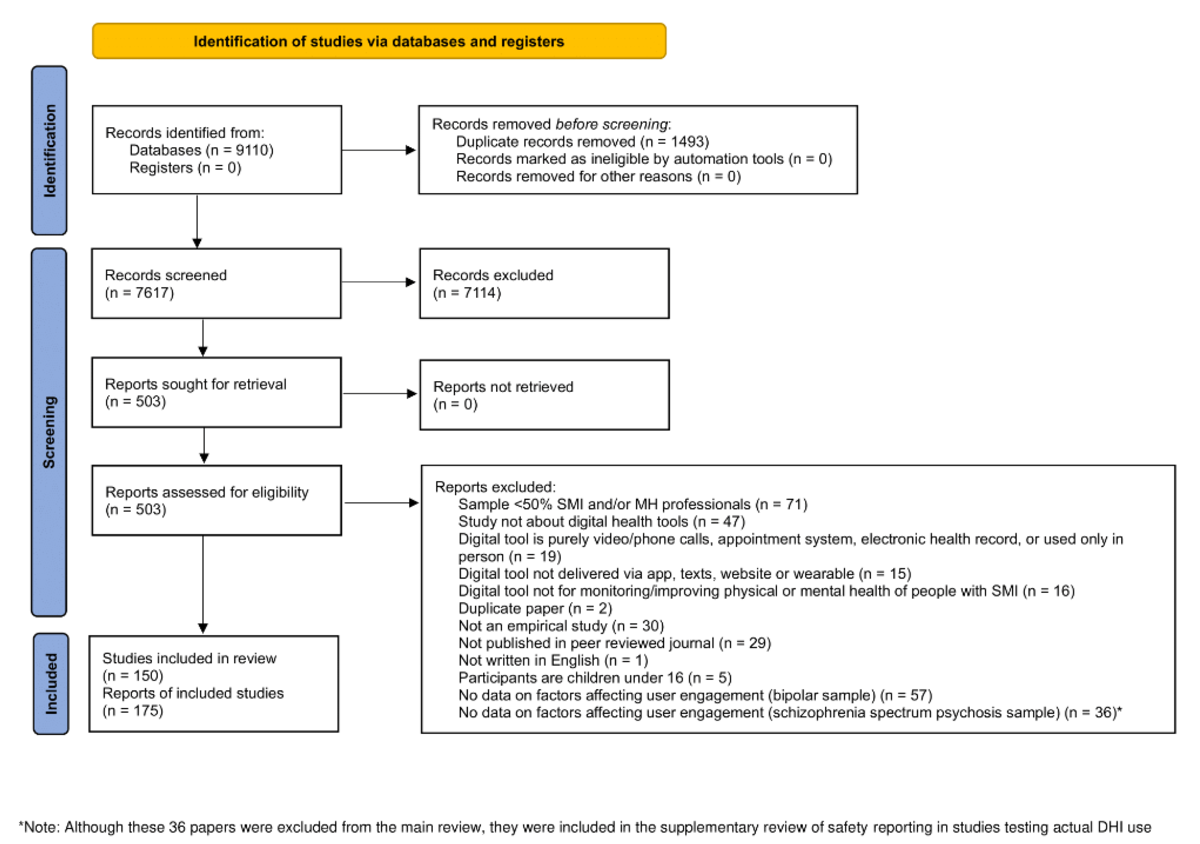
PRISMA (Preferred Reporting Items for Systematic reviews and Meta-Analyses) flow diagram.

Our search retrieved 7617 records after deduplication (Figure 1); 503 underwent full-text review, of which 175 were included based on inclusion and exclusion criteria. A list of the excluded studies and their reasons for exclusion are provided in Table S1 in Multimedia Appendix 2. Studies that did not contain data on barriers or facilitators of DMHIs were excluded (psychosis samples n=36; bipolar samples n=57). However, to supplement findings regarding the potential harms of DMHIs, we examined AE reporting in the 36 studies of psychosis samples.

### Description of Included Studies

We included 175 papers reporting 150 studies (11,446 participants). Tables S2-S5 in Multimedia Appendix 2 outline the study design, sample characteristics, and quality checks. Most DMHIs focused on general symptom management, relapse prevention, or similar. Some focused on a specific symptom (eg, cognitive difficulties and paranoia), and a minority on medication adherence, lifestyle, physical activity, or smoking cessation. Common technology types were mobile apps (77/150, 51.3% studies), websites (42/150, 28% studies), SMS text messaging (13/150, 8.7% studies), and wearables (15/150, 10% studies). Slightly more DMHIs were blended (ie, delivered with human support, 49/150, 32.7% studies) or stand-alone (24/150, 16% studies) than minimally blended (23/150, 15.3% studies) or included setup or technical input only (20/150, 13.3% studies). The median DMHI use was 8 (IQR 4-19) weeks. Study types included single-arm interventional studies (47/150, 31.3%), RCTs (25/150, 16.7%), qualitative studies (20/150, 13.3%), cross-sectional surveys (14/150, 9.3%), observational studies (7/150, 4.7%), intervention development studies (13/150, 8.7%), usability testing studies (11/150, 7.3%), and case studies (7/150, 4.7%). Most studies examined actual DMHI use (106/150, 70.7%), though some were hypothetical (ie, asking participants about potential DMHI use, 44/150, 29.3%), and some tested in-session usability rather than use in daily life (9/150, 6%). Samples included people with schizophrenia spectrum disorders (98/150, 65.3% studies), bipolar disorder (62/150, 41.3% studies), and clinicians (26/150, 17.3% studies). Participants were recruited from a range of settings, including inpatient services (21/150, 14% studies), community services (115/150, 76.7% studies), and online adverts (16/150, 10.7% studies). The Mixed Methods Appraisal Tool subsections (scored out of 5; Table S4 in Multimedia Appendix 2) indicated the moderate quality of qualitative (mean 4.1, SD 1.57) and quantitative (mean 4.0, SD 1.0) study elements, and lower mixed methods quality (mean 3.1, SD 1.9). The CONSORT harms checklist showed AE reporting to be mostly poor or missing (Table S5 in Multimedia Appendix 2); thus, it is difficult to draw informative conclusions regarding the harms of DMHIs.

### Findings Not Fitting Within CFIR Framework

Although most relevant findings were coded into CFIR, a few could not be coded: patterns of DMHI use over time, associations between demographic variables and DMHI engagement, and safety reporting findings.

#### Patterns of Use

Sixteen studies reported reduced DMHI use over time in this study, 7 reported stable use and none reported an increase.

#### Demographics

Demographic findings are outlined in the Table S6 in Multimedia Appendix 2. Only 28% (11/39) studies examining age found significant effects: 6 found higher engagement in older people, 4 in younger people, and 1 found mixed effects. Conversely, all staff participants in qualitative studies hypothesized higher DMHI engagement among younger people [31-35]. Only 28% (7/25) studies examining gender or sex reported significant effects; most (n=5) found higher DMHI engagement among women. Only 20% (4/20) studies examining education reported significant effects; all 4 found higher education associated with higher engagement. Only 20% (2/11) studies reported significant effects of race, ethnicity, or immigration: Ben-Zeev et al [36] reported significantly higher engagement among White participants than African American participants (and among White participants than Hispanic participants for some, but not all, engagement indices), while Bonet et al [37] reported significantly higher agreement to use a DMHI among non-native participants than native Spanish participants. Of studies examining the effects of IQ, 67% (2/3) showed significant effects, with higher IQ associated with higher DMHI engagement [38,39]. Reading level (0/1), employment (0/5), and marital status (0/2) showed no significant effects.

#### Safety Reporting

Multimedia Appendix 2 describes safety information extracted from 168 papers (139 studies) reporting actual DMHI use. Only 86 papers (76 studies) reported AE-related content; 23 reported AE frequency or percentage (median number of AEs reported per study 2, IQR 0.8-39.2). AEs included hospitalizations, psychological distress, suicidal ideation, physical health-related events, and death. Relatedness of AEs to the intervention was only reported in 11 papers, 8 of which deemed some events related.

### CFIR Coding

**Figure 2:**
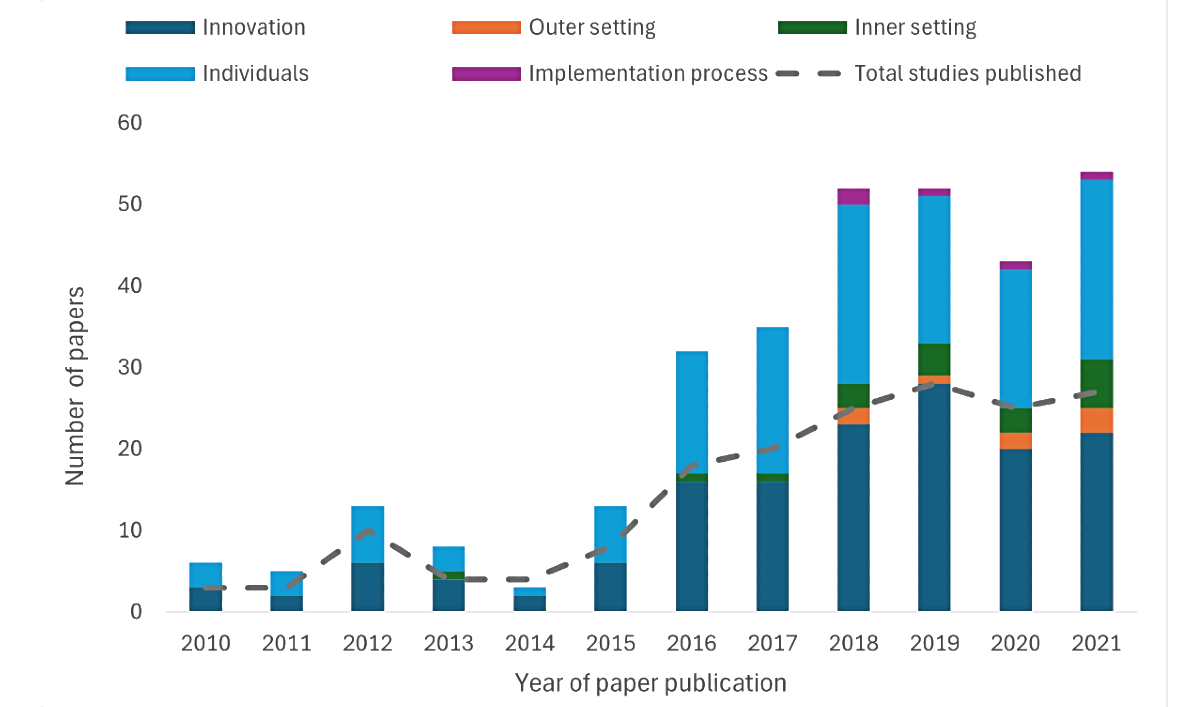
Number of papers coded into each Consolidated Framework for Implementation Research domain per year of publication.

Tables 1-5 provide detailed findings regarding the coding of reviewed studies into the 5 domains of the CFIR framework: *Innovation* (Table 1), *Outer setting* (Table 2), *Inner setting* (Table 3), *Individual characteristics* (Table 4), and *Implementation process* (Table 5). Findings were most frequently related to *Innovation* (Table 1; n=149 papers) and *Individual* (Table 4; n=137) domains of CFIR, with aspects of the *Outer setting* (Table 2; n=9), *Inner setting* (Table 3; n=20), and *Implementation process* (Table 5; n=5) domains rarely described. Figure 2 shows the number of papers coded into each CFIR domain per year of publication. Studies published between 2010 and 2015 were coded almost exclusively against the *Innovation* or *Individual* CFIR domains. Occasional studies were then coded against the *Inner setting* domain from 2016 onward, and against the *Inner setting*, *Outer setting*, and *Implementation process* domains from 2018 onward.

Prominent subcodes within the *Innovation* domain were *Relative advantage* (99/175, 56.6% papers), *Complexity* (56/175, 32% papers), and *Design* (128/175, 73.1% papers). As many papers commented on DMHI design, details of inductive subcodes are provided separately in Table 6. These included design features to increase DMHI engagement, customization, repetition vs variety, type of device used, user interface design, passive sensing, data security and privacy, and technical difficulties. *Need* (48/175, 27.4% papers), *Capability* (88/175, 50.3% papers), *Opportunity* (48/175, 27.4% papers), and *Motivation* (73/175, 41.7% papers) from the Individuals domain were all widely used, although almost exclusively in relation to innovation recipients rather than other roles. Details of how prominent findings from the CFIR-coded data related to the 5 crosscutting themes, are provided in the Crosscutting Themes section.

**Table 1:** Consolidated Framework for Implementation Research (CFIR) Innovation codes.

CFIR construct	Findings in relation to this construct	Papers, n (%)	Data type	Study type^a^	Sample
			Qualitative; quantitative; mixed	Hypothetical; usability; actual	Patient; staff; both
Source	Participants hypothesized that clear endorsement by a known institution (eg, health service or university) would increase engagement. One study [40] noted that online information from pharmaceutical companies was less trusted than other sources. Some preferred endorsement by individual staff because “organizations have hidden agendas” [41]. Perceived credibility of teams developing or testing the DMHI^b^ impacted engagement [42-44]. Teams that included people with lived experience of psychosis or bipolar disorder were especially credible. However, one study [45] highlighted that co-design is not a panacea; despite being co-designed, the study innovation (MindFrame) “was neither applicable nor appealing to all.”	14 (8)	11; 0; 3	7; 1; 8	10; 0; 4
Evidence base	A patient perspective piece [46] suggested that health care professionals discussing the evidence base for specific DMHIs with patients would likely increase engagement. Two surveys [47,48] collected mixed methods data on whether information on the evidence base of bipolar apps influenced users’ app choice. Qualitatively, “participants seldom reported evidence of efficacy or scientific quality to justify app choice,” but in quantitative responses, 93% [48] and 90% of participants [47] rated scientific quality as important. Health care professionals [49] cite a lack of knowledge regarding which apps were evidence based as the foremost barrier to discussing apps with patients with bipolar disorder.	4 (2)	1; 1; 2	4; 0; 0	3; 1; 0
Relative advantage	Connection with peers and normalization: qualitative data highlighted that DMHIs can facilitate peer connection and normalize psychosis or bipolar disorder experiences. Peer contact, both as a formal part of the intervention (peer facilitators or video resources) or via a bespoke or mainstream social media platform, provided “the opportunity to learn from others managing the same condition, reducing self-stigma through normalization of shared experiences” [50]. Peer support appeared to influence participants’ actual level of engagement with DMHIs [51], or participants hypothesized that it would [33,52,53]. Participants valued encouragement and accountability from others on the platform [54-57] and the opportunity to socialize with peers [58-62]. Even DMHIs without specific peer content were normalizing as their presence implied others with similar needs [63-65], and they used a similar format to everyday things (“...everyone uses an app these days... It’s normal now,” [41,64]). However, some were anxious that others would ask what the device or app was for [41,58,66,67], or preferred apps for “the general consumer, so I do not feel stigmatized as a patient for using it” [46].	43 (25)	31; 2; 10	18; 0; 29	31; 3; 9
Relative advantage	Availability and autonomy: reviewed studies highlighted DMHIs’ availability (anytime, anywhere) as a key advantage and reported that DMHIs increased participants’ autonomy. Availability was particularly beneficial for people constrained by working hours [52], childcare [52], geography [62,68,69], reversed sleep cycles [41], or during recovery from psychosis [41,52,70]. Patients contrasted DMHI availability with that of their care team [64] and valued DMHIs’ ecologic validity [71] and being able to visit, or revisit, content at their own pace [72-74]. Quantitative data [75] indicated similar app engagement off- and on-hours; ie, DMHIs are used outside clinic hours when available. Choosing when and where to use DMHIs enhanced feelings of autonomy, control, or empowerment [33,63], encouraging individuals to play an active role in managing their mental health (“They actually feel like they have part ownership in the process,” [76]) and decreasing reliance on health professionals (“it gives you a bit of freedom to say ‘hold on a second, I don’t have to wait for my CPN^c^’” [71]). Accessing information without a clinician’s direct involvement was particularly empowering, with patients frequently describing DMHIs as a friend or therapist. Patients and staff noted that internet-based mental health information ranges in accuracy, so providing “curated knowledge from a bunch of professionals rather than just what Google tells you” [68] was a significant advantage. Participants from 10 studies described a DMHI in human terms, including as a “CPN in your pocket” [71], “therapist in your phone,” “personal smoking trainer” [77], “life-coach” [78], “friend” [43,52,64,79,80], “buddy” [71], “close companion” [81] or “constant companion” [82]. Staff [33,83,84] and patients [34,41,52,55,63,64,68,71,85] across 12 studies highlighted a DMHI’s anonymity can be a substantial advantage. For example, some DMHIs provided an opportunity to vent without fears of a subsequent medication increase [63], to “scream on the keyboard” at any time [68], or to disclose sensitive or potentially disturbing information in a nonjudgmental, anonymous place instead of directly to a person [33,41,52,71,83,84]. A minority of participants viewed some automated DMHI functions as potentially detracting from autonomy; for example, automatic transfer of data from the app to services [31,66,78], and medication reminders [59]. Availability and autonomy contributed to perceptions of DMHIs as widening the reach of services. Symptom-monitoring or cognitive behavior therapy apps helped users feel safe with a more hands-off clinical approach (“it did... make me feel safe... I knew he was keeping an eye on those graphs,” [86]) while allowing prompt intervention if needed. More trivially, technology provided a convenient way to keep in touch with clinicians (eg, SMS text messaging, email, and instant messaging).	65 (37)	42; 8; 15	29; 2; 38	43; 7 15
Relative advantage	Self-awareness and memory: DMHIs’ availability helped patients notice patterns in their experiences (“to see what’s making me happy, what’s doing my head in” [31]), remember those experiences, and talk to others about them. Participants said DMHIs gave a useful structure for therapeutic conversations [66,86-91], promoting shared understanding, strengthening therapeutic relationships, helping patients feel “heard” [92] or “connected” [80,93,94], and facilitating help-seeking. One study highlighted a self-monitoring app’s value in translating individuals’ subjective experiences into an objective form, enabling others to access their inner experiences and take them more seriously (“If you were to answer the questions and go to the doctor and say, ‘look... you can see clearly there’s a change, and these are my experiences,’ that would be substantial evidence for the doctor to then sit up and take note” [64]). In some cases, this process was formalized as part of an Ecological Momentary Intervention [95,96]. One study drew a specific link between self-awareness and user engagement (“EMA^d^ self-monitoring and EMA-derived feedback were seen as helpful for improving awareness, highlighting a willingness to engage with more intense monitoring for clinical purposes” [95]. As well as sharing symptom data with clinicians, patients often shared it with family, facilitating communication, understanding, and support. However, 2 surveys reported that only a minority of patients [47,88] and staff [88] thought symptom information should be shared with family, and a qualitative study cautioned that users should always retain control over who had data access [78].	61 (35)	34; 9; 18	17; 0; 45	45; 1; 15
Relative advantage	Should enhance but not replace human care: qualitative studies outlined ways that DMHIs did not present a relative advantage over human care. Neither staff [84] nor patients [41] liked “fake empathy” offered by a machine, considering it degrading. Many saw DMHIs as a cost-cutting exercise [31,33,41,42,52], which might feel “dismissive” to patients, “as though they are not worthy of a clinician or therapist” [41], leading them to feel “fobbed-off” [33]. Some worried that using DMHIs blended within face-to-face sessions might render clinicians less “present” in the interaction [34,35,46] because they are “increasingly focused on a computer or on data rather than on the suffering human seated in front of them” [46]. Patients considered DMHIs a “poor substitute for seeing a person that knows you” [31], lacking personal contact [85,97], opportunities for “being listened to” [85], tone of voice, body language [68] and emotional reassurance [41], and unable to address everyday practical problems (eg, housing difficulties; [85]. Staff worried that DMHIs lacked nonverbal communication [33], context [31,33] and personalization [33,84], left patients “alone to deal with any issues that surface” [84], removed a key source of socialization and warmth, potentially reinforcing social avoidance [33], and that DMHI-generated data would be difficult to interpret without staff members’ clinical experience [31].	16 (9)	13; 0; 3	11; 0; 7	9; 4; 3
Adaptability	No studies provided information on whether the ability to adapt a DMHI to a specific service affected engagement. Personalization of DMHIs for individual users is coded under *Innovation design*.	0	—^e^	—	—
Trialability	An expert consensus study [83] and a patient perspective piece [46] highlighted that the availability of a free trial version of a DMHI would increase the likelihood that staff and patients would use it.	2 (1)	1; 1; 0	2; 0; 0	1; 1; 0
Complexity	Technology-related complexity, stemming from poor user experience design, increased user burden, and “mental fatigue” [98], prompting disengagement [69,96,98,99]. Lengthy login processes reduced engagement [69,88,89,100,101], as did difficult-to-navigate websites or apps (“I wanted to go and do it, but then I just couldn’t navigate it. And I was just like, ‘Oh, I can’t be bothered’,” [31]. Qualitative and quantitative data found shallow website and app structures, and pages with <200 words, most usable by patients. Unwieldy, multistep systems (eg, SMS text messaging–based symptom monitoring) were perceived as “awkward, laborious, and burdensome” [85] and associated with significantly lower engagement than a more streamlined native smartphone app [102]. One study noted that tailoring DMHI content increased complexity [86]. Patients suggested that training and support may mitigate the effects of technology-related complexity [34]. Individual capability (eg, cognitive ability, digital literacy; [34,86] also moderated the extent to which complexity hindered engagement and the amount of training that was needed to mitigate this effect [34,100]. Some studies noted that practical complexity, such as remembering to carry, charge, or use an additional device [66,103-105], hindered engagement. Although most issues of complexity were related to the technology, rather than the intervention more broadly, staff in several studies [31,33,69,92,106] found the information they received from symptom-monitoring apps complex to interpret, making it difficult to know how to respond clinically. Patients expressed concerns that some apps and websites had overwhelming amounts of information or features [55,107,108]. A patient perspective piece highlighting the fallacy of the “killer app,” preferring apps with a clear aim that do not try to do too much at once [46].	55 (31)	31; 10; 14	16; 7; 37	41; 2; 12
Design	As most reviewed studies commented on aspects of DMHI design, details are provided separately in Table 6.	128 (73)	56; 43; 29	38; 5; 93	101; 7; 20
Cost	Most participants (85%) in a large international survey of people with bipolar disorder (n=919) preferred free or low-cost apps [47] and qualitative survey data suggested DMHIs for bipolar should be “free or inexpensive so accessible to all or many” [73]. Five studies [109-113] listed financial costs associated with delivering a particular DMHI (eg, web hosting, licensing fees, texting fees, technical support, and staff time to deliver) but did not examine the effect on engagement.	9 (5)	1; 6; 2	4; 0; 6	7; 0; 2

^a^Some papers fall into more than one category, so categories may add to more than the overall total N.

^b^DMHI: digital mental health intervention.

^c^CPN: community psychiatric nurse.

^d^EMA: ecological momentary assessment.

^e^Not applicable.

**Table 2:** The Consolidated Framework for Implementation Research (CFIR) Outer setting codes.

CFIR construct	Findings in relation to this construct	Papers, n (%)	Data type	Study type^a^	Sample
			Qualitative; quantitative; mixed	Hypothetical; usability; actual	Patient; staff; both
Critical incidents	Patients and clinicians noted their use of technology had increased since the COVID-19 pandemic [106].	1 (1)	1; 0; 0	1; 0; 0	0; 0; 1
Local attitudes	—^b^	0	—	—	—
Local conditions	US clinicians [76] said local technological conditions were a major barrier to DMHI^c^ implementation. A lack of regional infrastructure (mobile phone signal) prevented app use by clinicians and patients. Many participants had government-sponsored smartphones, incapable of running the study app. Technological infrastructure is not a universal barrier though a survey with EIS^d^ clinicians across 30 US states indicated that 93% had high-speed internet available [114] and a study testing an app in a Canadian city indicated 100% mobile phone coverage [109].	3 (2)	1; 1 1	2; 0; 3	1; 2; 0
Partnerships	—	0	—	—	—
Policies and laws	Clinicians from Indian health services highlighted the need to understand regulations and abilities governing app-generated data before using apps with clients [106]; US clinicians in the same study did not raise such concerns. Study authors attributed the difference to recently introduced telehealth policies in India. Clinicians from Australia [35] and the United Kingdom [115] mental health services noted the lack of guidelines and policies as a barrier to DMHI engagement.	3 (2)	3; 0; 0	3; 0; 0	0; 1; 2
Financing	Empirical results did not directly report on financing in relation to DMHI engagement or implementation, but study authors noted that a sustainable business model for maintaining evidence-based DMHIs is often absent. One study noted that costs were offset by the billable nature of the service [110].	3 (2)	1; 0; 2	2; 0; 2	2; 0; 1
External pressure	—	0	—	—	—

^a^Some papers fall into more than one category, so categories may add to more than the overall total N.

^b^Not applicable.

^c^DMHI: digital mental health intervention.

^d^EIS: early intervention service

**Table 3:** The Consolidated Framework for Implementation Research (CFIR) Inner setting codes.

CFIR construct	Findings in relation to this construct	Papers, n (%)	Data type	Study type^a^	Sample
			Qualitative; quantitative; mixed	Hypothetical; usability; actual	Patient; staff; both
Structural characteristics	IT: poor IT infrastructure, including aging computers [62], poor bandwidth [35,62], and no wireless connection in hospitals [116] hindered engagement. Staff in one study expressed low confidence that their organization could implement DMHIs^b^ due to its historical failures to successfully deliver past IT projects [33].	4 (2)	3; 0; 1	2; 0; 2	1; 2; 1
Structural Characteristics	Physical infrastructure: a lack of quiet space in the clinic hindered implementation [62].	1 (1)	0; 0; 1	0; 0; 1	0; 0; 1
Structural characteristics	Work infrastructure: work infrastructure impacted staff DMHI engagement in one study [79]. Local mental health teams had been relocated and merged shortly before the DMHI was introduced. Study authors hypothesized that staff hesitated to fully engage with the DMHI for fear that it would increase their workload further.	1 (1)	1; 0; 0	0; 0; 1	0; 0; 1
Relational connections	—^c^	0	—	—	—
Communications	—	0	—	—	—
Culture	Human equality-centeredness: none	0	—	—	—
Culture	Recipient-centeredness: staff felt was it important to offer patients a choice to use a DMHI: “It’s also nice to think, to be able to give someone an option that you’re not forcing down their throat” [117]. Patients in this study appreciated this attitude, although the impact on DMHI engagement was not directly investigated.	1 (1)	1; 0; 0	0; 0; 1	0; 0; 1
Culture	Deliverer-centeredness: none	0	—	—	—
Culture	Learning-centeredness: none	0	—	—	—
Tension for change	—	0	—	—	—
Compatibility	The lack of compatibility between DMHIs and existing workflows was a key barrier to engagement and implementation. Clinicians believed DMHIs would create extra work, a major barrier in overstretched services [31,35,76,79,83]. Some DMHIs duplicated existing work [34,76]: “reporting symptoms via the app system, instead of saving time, added another workflow to manage.” Participants often questioned who should be responsible for managing and responding to real-time symptom data [33,69,84,92,106], particularly out-of-hours [33,80,84,85,92], as doing so did not fit well with their current ways of working. Several studies emphasized that compatibility with existing electronic medical record systems would be important [88,118,119]. Clinicians in one study [84] strongly suggested patients should bring symptom-monitoring data to appointments, rather than uploading data in real-time. Although arguably undermining a key advantage of DMHIs (real-time data), this suggestion highlights how challenging the staff find the idea of being presented with a “constant stream of information” [33], and the perceived additional responsibility that comes with that information.	17 (10)	11; 3; 3	9; 0; 9	2; 5; 10
Relative priority	—	0	—	—	—
Incentive systems	“Reimbursement by payers for time spent training patients and family members about DMHI and for time spent using or reviewing data from the DMHI” was a strong potential facilitator of HCP^d^ engagement with DMHIs [83]. Similarly, the “difficulty having the DMHI approved by insurance” was considered a substantial barrier to implementation [83].	1 (1)	0; 1; 0	1; 0; 0	0; 1; 0
Mission alignment	Although staff appeared to consider some aspects of DMHIs aligned with their mission (see *Relative advantage* in Table 1), 5 studies highlighted ways that implementing DMHIs was not aligned with the inner setting’s central mission. Staff felt funding was “channeled into technological advancement when perhaps it would be better channeled into staffing and training” [33]. Given limited time, clinicians considered other on-mission tasks more important than using DMHIs; eg, emergent crises [119], the client’s own priorities [34,35], and simply having “more pressing things to deal with” [34]. Staff in one study felt “a huge burden of responsibility toward protecting their clients from harm and take this responsibility incredibly seriously” [33]; they perceived some aspects of DMHIs to present additional risks, directly conflicting with this perceived mission. Other staff were cautious of DMHIs disrupting the trust they had built with clients (“I’m not prepared to do anything that could damage that relationship... you work so hard to get that trust,” [35]); hence, they would always prescreen DMHI content before showing it to a client.	5 (3)	4; 0; 1	3; 0; 2	0; 3; 2
Available resources	Materials and equipment: in response to digital exclusion challenges, it has been suggested that health services could provide devices to facilitate DMHI access. None of the reviewed studies directly investigated the impact of this, but staff in one study “felt the NHS should not provide the required technology because of concerns that tablets and mobile phones may get lost, sold, or damaged” [84]. However, across 15 reviewed studies that reported device loss rates, only 112 (11.1%) of 1011 provided devices were lost, broken, stolen, or damaged. Staff [80] and patients [70] also mentioned the need for devices for staff themselves, to facilitate blended DMHIs; eg, for SMS text messaging–based DMHIs [80], or as a backup if the data connection is lost during video-based aspects part of a blended DMHI [70].	20 (11)	7; 6; 7	5; 0; 15	14; 2; 4
Available resources	Funding: none	0	—	—	—
Available resources	Space: none	0	—	—	—
Access to knowledge and information	Providing staff with training in basic digital skills [33,114], how to evaluate DMHI credibility and quality [49,114], and how to use specific DMHIs [34,49,83] was hypothesized to facilitate engagement. One participant said, “There’s no point in [a patient] being a whizz on that computer and smartphone and me not having a clue cos I wouldn’t be able to support adequately, there would be... ongoing training needs,” [33]. Even staff who were digitally confident felt they would need more training to assist others [114] and lacked knowledge about which DMHIs are credible [49,114]. As well as clear training or instructions on how to support patients with specific DMHIs [49,83], staff also valued the opportunity to try the DMHI themselves as part of this training [34].	5 (3)	2; 3; 0	4; 0; 2	0; 4; 1

^a^Some papers fall into more than one category, so categories may add to more than the overall total N.

^b^DMHI: digital mental health intervention.

^c^Not applicable.

^d^HCP: health care professional.

**Table 4:** The Consolidated Framework for Implementation Research (CFIR) Individual codes.

CFIR construct	Findings in relation to this construct	Papers, n (%)	Data type	Study type^a^	Sample
			Qualitative; quantitative; mixed	Hypothetical; usability; actual	Patient; staff; both
Need	Perceived benefit: users need to perceive a DMHI’s^b^ benefit to try it (“People do not do things just because their doctors tell them to. They need to see the benefit of what they do,” [46] and continue using it (“If people think an app is helping them, they will continue using it,” [48]). Clinicians noted that *quickly* finding benefits helped sustain use: “People tend to download the app and then it decreases in use if people don’t see a quick benefit” [49].	34 (19)	16; 10; 8	10; 0; 25	24; 4; 6
Need	Stage of illness: qualitative data highlighted that needs may differ depending on patients’ stage of illness or recovery and that DMHI content (eg, psychoeducation) and functions (eg, symptom tracking) are often best suited to the needs of people with recent onset illness or ongoing symptoms. Quantitative data implied effects on engagement: people with recent onset psychosis were more likely to use an app than those with established psychosis (037); those with longer psychosis duration rated digital monitoring as less important to managing their illness than more recently diagnosed people. Several studies reported DMHI discontinuations due to improved mental health [54,120,121]. Some adapted their DMHI use to meet their own needs [122].	27 (15)	14; 8; 5	7; 0; 21	21; 1; 5
Capability	Physical capability: 4 studies mentioned that medication side effects (eg, fine motor tremors and blurry vision), interfered with participants’ ability to use a DMHI [80,93,123,124].	4 (2)	2; 0; 2	1; 0; 3	3; 0; 1
Capability	Psychological capability: Table S7 in Multimedia Appendix 2 outlines data from studies quantitatively examining the effects of specific symptoms, functioning, and diagnosis on DMHI engagement. Around a third of quantitative analyses found a significant effect of symptoms on engagement, with higher baseline positive, negative, or cognitive symptoms associated with lower DMHI engagement. Results for functioning and depression were more mixed. Quantitative studies did not examine the impact of relapse on DMHI engagement, but 11 qualitative studies noted decreased DMHI use during relapse due to thought disorder (“I wouldn’t have been able to make sense of the topics,” [51]), using a DMHI feeling “too confrontational” [92], avoiding certain symptom-related questions [64], difficulty staying awake during app training [93], or lack of energy or motivation to engage [121] when unwell. Nevertheless, relapsing participants were typically able to continue using a DMHI once symptom exacerbation had resolved [51,64], and one study noted that, for some, stopping the app might be a sign of imminent relapse [64]. A few participants avoided DMHIs due to interactions between their symptoms and technology, such as delusions or paranoia about technology [37,64,86,104], adverse effects of technology in mania [46,82], or social anxiety during DMHI social media use [54]. Some variables relating to psychological capability were examined in very few studies. Neither baseline biological rhythm disturbance [125] nor baseline medication adherence predicted DMHI engagement [94,126,127], although those who chose to use a DMHI were less likely to be using injectable medication [37]. Better premorbid adjustment was associated with higher DMHI engagement [128]. Cannabis use predicted lower DMHI engagement [99].	76 (43)	26; 33; 17	16; 0; 61	64; 4; 8
Capability	Digital literacy: lack of digital literacy was a barrier for some, and expert consensus participants considered self-efficacy beliefs about using devices as highly likely to promote DMHI engagement [83]. Some clinicians reported lacking the technological capability to deliver a DMHI [33,34,114]. Three studies quantitatively examined the effects of patients’ digital literacy on DMHI engagement. Two reported no significant effect [96,109]; in one study participants with prior smartphone experience engaged significantly more in a digital than a nondigital intervention, whereas no such difference was observed for people without previous smartphone experience.	33 (19)	14; 10; 9	9; 3; 22	24; 6; 3
Opportunity	Space: participants reported a lack of a suitable environment as a barrier. Busy homes, hospitals, public libraries, or homelessness presented challenges of noise, repeated interruptions, and lack of privacy. For blended DMHIs, the in-person location was sometimes a barrier [89,110,129].	12 (7)	7; 2; 3	1; 0; 11	9; 1; 2
Opportunity	Time: lack of time due to work [98,130], physical illness [131], preoccupation with delusions [43], or other commitments was a barrier to engagement for some patient participants. Conversely, being able to fit the DMHI within their daily or weekly routine was a facilitator, and inpatient participants enjoyed using a DMHI as “something to do” [116].	22 (13)	7; 10; 5	1; 0; 21	20; 0; 2
Opportunity	Technology: lack of access to technology was a barrier. This included not owning a phone, tablet, or computer [34,62,113,128], or having a device with limited functionality [76], insufficient storage [52], insufficient data allowance [41,132], poor internet connection [132], or frequently changed phone numbers [80,84]. Staff highlighted a key difficulty with using technology for health care: “phones are... a luxury item... if people aren’t working because of health needs... they are being denied healthcare because they can’t afford a phone” [33].	30 (17)	15; 12; 3	16; 0; 15	19; 6; 5
Motivation	General: some were motivated by financial or material factors (eg, vouchers or free study devices). Others described intrinsic motivations: commitment to participating [44], wanting to provide accurate data [31], agreement with DMHI tasks or goals [83], less “controlled motivations for treatment” [133], and interest in technology [83]. One study noted that people who tended to be more engaged with services were also more engaged with the DMHI [76].	12 (7)	7; 3; 2	4; 1; 9	9; 2; 1
Motivation	Support: across 45 qualitative studies, the following types of human support were mentioned: training in basic digital skills, managing technical difficulties, support navigating the DMHI, ongoing coaching to sustain use, discussion of psychoeducational materials, and interpreting or responding to changes in real-time symptoms data. Four randomized controlled trials quantitatively compared unsupported vs supported DMHI engagement. Two reported significantly higher engagement in a peer-supported group than in an unsupported group (*P*<.05 [121,134]; *P*=.005 [135]). One reported a significant interaction effect such that reviewing symptoms had a significant positive effect on app adherence only for younger women (*P*=.001), with no significant overall effect [136]. Finally, a pilot randomized controlled trial found moderate effect sizes in favor of the group allocated to support from a clinical helper (not powered to detect significant differences [137]). Regarding social support more generally, there were mixed quantitative results. One study reported lower social support among more actively engaged participants [61], with a second reporting the opposite pattern [138].	48 (27)	24; 13; 11	13; 1; 39	36; 4; 8
Motivation	Fear of relapse (barrier): anticipated or actual anxiety about DMHI use was sometimes a barrier. Staff [52] and patients [44,132] hypothesized that using a DMHI may increase awareness of symptoms, heightening users’ anxiety about deterioration, and potentially making symptoms worse. There was some evidence to indicate that this belief may hinder engagement. Quantitatively, users reporting higher fear of relapse at baseline showed lower engagement with a symptom-monitoring app over the following 6 months [126]. Elsewhere, one participant reported: “Being notified of all the changes sometimes made me anxious. It made me wonder if the illness was maybe about to get out of control” [45] and a minority of participants in other studies reported increased rumination [66,86,102]. This could be mitigated to some extent by support: “It really helped to have her sit there and talk things through with me... and just to have her to bring me back down, or bring me back into reality” [117]. Others were hesitant to (accurately) report symptoms via a DMHI for fear that it may lead to more restrictive treatment, such as hospital admission or increased medication [52,64,68,84].	26 (15)	11; 5; 10	8; 0; 19	20; 3; 3
Motivation	DMHI too confronting (barrier): some participants found DMHIs to be confronting: being reminded about their diagnosis or noticing how unwell they were, was disheartening and often prompted disengagement. A striking number of quotations related to this, for example, “My best days are when I typically forget that I have any kind of illness... to be very heavily reminded about the gravity of it all can be a bit unpleasant [51];” “It started me comparing how I am now to how I used to be... and its not good. That’s why I canceled it. I found it too personal in the end [85];” “I wasn’t ready to accept the illness... I wasn’t willing to change my life according to the program [121].” In one study [92], half the patients felt negatively influenced by frequent confrontations with their symptoms. In another study [88] only a minority (4%) found the DMHI confronting and others reported that it helped them recognize their progress. One study quantitatively examined if recovery style predicted DMHI engagement, finding no significant effect [133].	11 (6)	7; 3; 1	2; 0; 9	8; 0; 3

^a^Some papers fall into more than one category, so categories may add to more than the overall total N.

^b^DMHI: digital mental health intervention.

**Table 5:** The Consolidated Framework for Implementation Research (CFIR) Implementation process codes.

CFIR construct	Findings in relation to this construct	Papers, n (%)	Data type	Study type^a^	Sample
			Qualitative; quantitative; mixed	Hypothetical; usability; actual	Patient; staff; both
Teaming	—^b^	0	—	—	—
Assessing needs	Innovation deliverers: none	0	—	—	—
Assessing needs	Innovation recipients: a patient perspective piece highlighted the current lack of assessment of patient needs regarding DMHI^c^ implementation: “It is important for clinicians to explore with their patients what kinds of problems they are facing living their day-to-day lives and whether there are apps that can assist them” [46].	1 (1)	1; 0; 0	1; 0; 0	1; 0; 0
Assessing context	—	0	—	—	—
Planning	—	0	—	—	—
Tailoring strategies	—	0	—	—	—
Engaging	Innovation deliverers: none	0	—	—	—
Engaging	Innovation recipients: there were mixed findings regarding whether a recommendation from a health professional increased DMHI engagement. Three out of 4 studies reported that such a recommendation would usually prompt engagement [41,106,119], particularly in the context of a strong relationship between a patient and staff member: “I trust the early intervention team... so I would be fairly confident that it would be secure if they said so” [41]. Patients in another study were asked whether they would use an app recommended by a clinician and all said “no” [115], though reasons for this were not outlined.	4 (2)	3; 0; 1	3; 0; 1	1; 0; 3
Doing	—	0	—	—	—
Reflecting and evaluating	—	0	—	—	—
Adapting	—	0	—	—	—

^a^Some papers fall into more than one category, so categories may add to more than the overall total N.

^b^Not applicable.

^c^DMHI: digital mental health intervention.

**Table 6:** The Consolidated Framework for Implementation Research (CFIR) Innovation design inductive subcodes.

CFIR construct	Findings in relation to this construct	Papers, n (%)	Data type	Study type^a^	Sample
			Qualitative; quantitative; mixed	Hypothetical; usability; actual	Patient; staff; both
Digital reminder	Most participants found digital reminders (eg, push notifications or email) to engage with the DMHI^b^, or remember medication or appointments, helpful; some found them nagging [123], irritating [86], “pushy” [47], or repetitive [96]. Ideal timing and frequency of alerts were often discussed, eg, personalizing alert timing and frequency [47,99,139], allowing users to “snooze” alerts to receive a later reminder [64], considering users’ specific needs and schedules (eg, afternoon alerts for those sleeping late due to medication; [64,79], and allowing self-initiated use [64,107]. Studies testing self-initiated use report 47% [75], 52% [43], 63% [140], and 97% [141] of app engagements were self-initiated rather than in response to app alerts. Two quantitative studies examined the impact of alerts on engagement: individuals randomized to receive weekly emails had significantly higher DMHI engagement than a comparison group [133]; 10-times daily reminders were significantly more inconvenient than daily monitoring [66].	57 (33)	26; 14; 17	16; 0; 43	45; 2; 10
Gamification	Most participants across 12 studies thought gamification would increase engagement though one reported mismatching qualitative (positive) and quantitative (neutral or negative) survey data on this, and another that focus groups barely mentioned it [78]. Three studies testing DMHIs with gamification elements [77,89,142] reported impacts on enjoyment and engagement (eg, “I really like the badges idea... that really helped me to keep going,” [142]).	13 (7)	9; 1; 3	8; 0; 6	11; 1; 1
Feedback from the DMHI	Qualitative data suggested DMHI feedback would increase engagement; eg, a symptom data summary, symptom graph, summary of passive data (eg, step count), a summary of DMHI engagement (eg, minutes used) or encouraging messages. Two studies quantitatively investigated the effects of feedback on engagement; one reported significantly higher Fitbit wear rates in those receiving feedback than a nonrandomized comparison group [143]; another found no significant between-group difference in symptom-monitoring app use [144].	29 (17)	13; 7; 9	8; 0; 22	23; 0; 6
Tailoring, customization, personalization	“Tailoring,” “customization,” or “personalization” of DMHIs was a facilitator, as mental health experiences vary hugely (“10 people with the same diagnosis as me will have 10 different ways of experiencing the illness,” [79], and DMHI users have different symptoms, treatment goals, schedules, illness durations, and learning styles. Customizing aspects of a DMHI is advantageous, eg, alert schedules [47,71,145], symptom questions [39,64,146], self-management strategies [46,50], psychoeducation content [63,73,97,107,109] and delivery method [147]. Personalizing more superficial elements (eg, colors, [64]), was occasionally mentioned but much less prominent. Indeed, a survey of people with bipolar disorder [48] found 92% of participants rated “flexibility/customization” (DMHI frequency or what things are tracked) as important, 84% rated “tailored content” as important but only 33% rated “personalization” (changing interface or colors) as important.	34 (19)	19; 4; 11	12; 0; 23	26; 2; 6
Repetitiveness and variety	DMHI repetitiveness was a barrier (18 studies) and greater variety in DMHI content was a potential facilitator (12 studies). Participants “wanted more tips and strategies... to minimize repetition and boredom, particularly toward the end of the intervention period when users had selected the same domain repeatedly and started receiving the same tips and strategies” [139]. “Periodic content updates or staggered unlocking of content based on the user’s evolving needs” would encourage repeated use [48,97]. A patient testing a cognitive behavior therapy–based app suggested adding different “levels” for different needs: “Level one... at the beginning of their recovery... starting to learn about the illness. Level 2 is in the middle... they’re getting more self-awareness or understanding of the illness and how to cope with certain symptomatic episodes. And then you have the advanced level” [97]. There was some coding overlap with personalization [44,65,97], as a wider variety of content would allow people to select content that is more relevant to their needs.	27 (15)	13; 5; 9	7; 0; 21	22; 0; 5
Type of device used	Participants preferred smartphone apps more than computer-based [35,61,88,101,132] or SMS text messaging–based systems [85,102], reporting that smartphone apps were more convenient and easier to integrate into daily life. SMS text messaging contact was preferred to emails by most (87%, 74%, and 82%, respectively [148-150]). A comparison of smartphone app and wearable use for >6 weeks in people with bipolar disorder [136] reported a significant time-by-device interaction: participants used the wearable more at study start and the app more at the study end. Subjective reports indicated more (72%) participants would use a wearable for a year than the app (47%) and that the wearable required less effort and was slightly easier to use. Most participants (80%) in a small survey of people with bipolar disorder said they would use a wearable device but would not want stationary installations of sensors in their homes [81]. Five studies mentioned the importance of DMHIs being compatible across Android and iOS devices [76,99,107,109,141].	28 (16)	13; 9; 6	7; 1; 23	22; 2; 4
User interface design	Clear language was a facilitator of patient engagement and complex language was a barrier. Clinicians recommended that DMHIs should be usable by people with low literacy [151] and in languages other than English [49]. The “tone” of messages or information presented in DMHIs was important to users. The ideal tone was positive, relevant, validating, motivating, and encouraging without being patronizing, glib, or “mind-numbingly positive.” Given that users were people with experiences of mental health problems, some felt that an overly positive tone hindered discussion of difficult topics [54]. Users preferred visually appealing DMHIs (eg, attractive colors, modern-looking interface, and suitably sized fonts), which presented information in a variety of formats (video, text, audio, graphics) and were engaging and interactive. If DMHIs include personas or characters, these should be diverse in ethnicity and gender [107,152]. Graphs, while popular, were not always easy for patients to understand [153]. Connectivity with other apps was not generally important to users [47].	46 (26)	28; 5; 15	19; 5; 27	35; 2; 9
Passive sensing	Patients often considered passively gathered data to be more objective and accurate than self-reported data [82,105,106] though some highlighted the possibility of false positives and inaccuracies and suggested that DMHIs include disclaimers acknowledging this [66,82,145]. Two studies highlighted a tradeoff between increased data accuracy provided by passive sensing and increased agency and self-efficacy provided by active symptom monitoring [69,82]. Some participants found the idea of passive sensing invasive [106,154,155], whereas others were unconcerned [59], or embraced the idea: “I know that my phone knows everything about me. Why not get it to help me?” [106]. Data indicated that participants were most enthusiastic about passive sensing being used to track sleep [58,59,81,82,145] and activity patterns [58,59,81,82]. There was some evidence patients were willing for data to be passively gathered about neurocognition [81], psychophysiology [81], medication adherence [59], and heart rate [82]. Audio and video data collection was less popular [81]; GPS and social media usage data collection was less popular still [81]. Participants with bipolar were positive about an “intelligent” system that “learns” their routines and automatically detects changes [69,82], and staff said that passively collecting data provided an overwhelming amount of information [106].	11 (6)	5; 2; 4	7; 0; 4	8; 0; 3
Data security and privacy	Data security and privacy concerns were frequently mentioned barriers, including general concerns about security breaches and mental health-specific concerns. Some associated DMHIs with surveillance [45,89] or emphasized that mental health stigma made them worried about sensitive symptom data being disclosed [33,47,80,82]. Measures to protect data or mitigate the effects of data loss included the use of pseudonyms, password protection, and the use of general terms (eg, “illness”) rather than specific diagnoses. Five studies suggested, including a clear statement about what would happen to data, though, staff and patients in a study that included such a statement admitted they did not read it [106].	36 (21)	23; 5; 8	20; 0; 18	25; 2; 9
Technical difficulties	Twenty-five studies reported that participants experienced technical difficulties with the software (eg, notifications firing multiple times in error) or hardware (eg, poor battery life). These usually affected a minority; responses varied from mild irritation to total DMHI disengagement.	25 (14)	5; 13; 7	1; 0; 24	23; 0; 2

^a^Some papers fall into more than one category; therefore, categories may add to more than the overall total N.

^b^DMHI: digital mental health intervention.

### Crosscutting Themes

### Crosscutting Theme 1: DMHIs to Meet Specific Needs, Not Replace Human Care

Findings emphasized that DMHI functions and content should align with specific patient needs and that patients and staff must perceive a benefit if they are to engage with a DMHI (n=34 studies; *Individuals, Need*; Table 4). Patients preferred DMHIs with a clear aim, which did not try to do too many things (*Innovation: Complexity*; Table 1). Several DMHI benefits came through clearly in reviewed studies (*Innovation: Relative advantage*), including their value in being available anywhere and anytime (n=37 studies), promoting autonomy (n=26), facilitating self-awareness and memory (n=61), connecting people with peers (n=29), and normalizing mental health experiences (n=26; *Innovation: Relative advantage*). These advantages contributed to perceptions that, if successfully implemented, DMHIs could widen the reach of services. Nevertheless, while patients and staff acknowledged that DMHIs might enhance care in specific ways, they emphasized that they should not replace human care (n=16; *Innovation: Relative advantage*) and staff worried that the increasing emphasis on DMHIs might detract from health services’ ability to deliver care (*Inner setting: Mission alignment*; Table 3).

Seeing the benefit of a DMHI in meeting their needs prompted patients’ initial use and helped sustain use (*Individuals: Need*). Studies (n=27) highlighted that patient needs may change over time, depending on illness stage and symptom levels. Certain DMHI content (eg, psychoeducation) and functions (eg, symptom tracking) may be best suited to the needs of people with recent onset illness or ongoing symptoms. Indeed, 3 studies [54,120,121] testing DMHI use qualitatively reported discontinuations due to improved mental health, as participants no longer felt they needed the DMHI. Tailoring or customization (*Design*; Table 6) allows DMHIs to be adapted for varied needs; eg, different psychoeducation “levels,” staggered unlocking of content based on users’ evolving needs, or adaptive frequency of symptom monitoring depending on symptom levels.

While staff considered some aspects of DMHIs (*Innovation: Relative advantage*) aligned with their core “mission”, they saw human contact as a central tenet of their mission that was potentially undermined by DMHIs (*Inner setting: Mission alignment*). Staff worried that “patients would see using the app system as a replacement for care, something it was not designed to do” [76], and doubted DMHIs could be effective without a therapeutic relationship: “If you are going to talk evidence-based, the biggest thing in every single intervention... is therapeutic relationship, so you are just removing the most effective part” [33]. Such views could be a prominent barrier to DMHI implementation, particularly for stand-alone or minimally blended DMHIs. This underlines the importance of clearly communicating the specific purpose of the DMHI, and its limits, and reassuring staff that it is not intended to replace them.

### Crosscutting Theme 2: Human Support for DMHIs

Forty-nine studies highlighted human support as a key facilitator of DMHI engagement (*Individuals: Motivation;* Table 4), underlining a need to align DMHI implementation with clinical workflows. Reviewed studies found a lack of compatibility with current job roles and workflows (n=17), with DMHIs seen as creating extra work in already stretched teams (*Inner setting: Compatibility;* Table 3) and as less important than other tasks (*Inner setting: Mission alignment*). Clinicians did not want to receive a “constant stream of information” about patients [33]. They thought symptom monitoring-based DMHIs would increase their responsibilities, and questioned who would respond out-of-hours. Staff lacked time to facilitate DMHI use, found data from symptom-monitoring apps complex to interpret (*Innovation: Complexity;* Table 1), and sometimes lacked digital skills (*Inner setting: Access to knowledge and information*).

Data on types of support needed suggested that not all support must be provided by someone clinically trained (*Individuals: Motivation*). Support needs varied, depending on the innovation’s or individual’s status on other CFIR constructs (see CFIR codes in parentheses), but included: training in basic digital skills (*Individuals: Capability*), recommending suitable DMHIs, onboarding, managing technical difficulties (*Design*; Table 6), support navigating DMHIs (*Innovation Complexity*), ongoing coaching (*Individuals: Motivation*), moderation of social media components, and interpreting and triaging real-time symptom data. Arguably, these tasks could be undertaken by a suitably trained nonclinical staff member, with referrals made to clinicians as needed (eg, when symptoms increase above a predefined threshold, or risk is indicated). Similar observations have prompted the idea of restructuring services to include new job roles for DMHI facilitation (eg, digital navigator, peer supporter). Reviewed studies did not test the implementation of such roles within clinical services; although, many DMHIs were successfully delivered by someone in an equivalent role in a research context. Of 4 RCTs quantitatively examining the effects of support, 2 demonstrated significantly higher DMHI engagement in peer-supported than unsupported trial arms [121,134,135], 1 found a significant positive effect of brief interviewer-facilitated symptom reviews on app adherence for younger women (no significant overall effect [136]), and 1 found moderate effect sizes in favor of the supported group [137] but was not powered to detect a significant difference (*Individuals: Motivation*).

Six studies provided evidence regarding whom staff or patients might prefer to deliver DMHI support (ie, clinicians or nonclinicians). Studies with staff suggested a preference for nonclinician support for initial onboarding [83], technical [83,114,156], or peer-supported [114] aspects of DMHI use, with input from a clinician or team leader for more clinical aspects [114,157]. Patients expressed mixed views, with some preferring support from a known clinician [68,157], but others viewing this as more “intrusive” than nonclinician support [122]. Qualitative studies reported that, as well as overcoming practical barriers (eg, poor digital literacy, technical difficulties, complex DMHI layout), human connection helped address barriers like low motivation and anxiety about DMHI use, and provided accountability: “I’m going to see [coach]... within a few days, and we were going to discuss this, then there was accountability, I had to get some things done” [87]. Peer support was valued, whether as a formal part of the intervention (peer facilitators) or via social media (*Innovation: Relative advantage*), as peers “...can relate to each other and they know what they’ve been through” [54], and participants did “not feel scared people will judge you because they have been through the same thing and will be supportive” [55].

### Crosscutting Theme 3: Practical Helps and Hindrances

Reviewed studies outlined practical factors that can help or hinder DMHI implementation. Arising across CFIR constructs, these factors tended to hinge on the principle that DMHI use should be as low-effort as possible for users, and practical barriers to access should be addressed.

To maximize engagement, DMHIs should ideally be free or inexpensive to patients (*Innovation: Cost;* Table 1), use similar formats to everyday technology (eg, smartphone app), and allow on-demand, out-of-hours use (*Innovation: Relative advantage*). DMHIs should use straightforward language, be visually appealing (*Design*; Table 6), minimize complexity, avoid lengthy login processes, use an easy-to-navigate app or website structure (shallow structure, <200 words/page), and be compatible with users’ own devices (*Innovation: Complexity*). Patients found smartphone apps more convenient than computer or SMS text messaging systems, though apps must be thoroughly tested to minimize technical difficulties (*Design*). Other design elements to increase engagement included push-notification reminders; gamification features (eg, badges for completed tasks); automated feedback about symptoms or DMHI use (eg, symptom graph, step count, and minutes DMHI used); and tailored or varied content to minimize repetition.

These factors interact with each other. For example, tailoring DMHIs may increase engagement, but can also increase complexity, an engagement barrier. In turn, other factors, such as training and support, and individual capability (eg, cognitive ability and digital literacy) may moderate the negative effects of complexity on engagement. Nevertheless, even with training and support, and high individual capability, more complex interventions require more time and effort from users, making disengagement more likely. DMHI design teams must weigh the relative advantages and disadvantages of such interacting factors.

Some engagement barriers could be mitigated given sufficient resources. Lack of access to technology (*Individuals: Opportunity*; Table 4) and lack of digital literacy (*Individuals: Capability*) could be addressed, in theory, by providing devices and training in how to use them. Although staff thought patients would lose, damage, or sell devices, reviewed studies found only an 11% loss of study-provided devices. Data security and privacy concerns were frequently mentioned barriers; patients were concerned about sensitive symptom data being disclosed, due to the potential stigma associated with it. Measures to protect data or mitigate the effects of data loss included the use of pseudonyms, password protection, and the use of general terms (eg, “illness”) rather than specific diagnoses.

Some broader practical barriers may be difficult for implementation teams to address directly. These included the lack of mobile phone signal, publicly-funded smartphones being too basic, lack of national guidelines and policies on DMHI use in health services (*Outer setting*; Table 2), aging computers, poor bandwidth, no wireless connection in hospitals, poor organizational support for IT developments, and lack of quiet space (*Inner setting*; Table 3), patient time, or suitable environment (*Individuals: Opportunity*).

### Crosscutting Theme 4: Interactions Between Symptoms and DMHI Engagement

Approximately one-third of quantitative analyses (18/52, 35%) reported a significant effect of symptoms on DMHI engagement (*Individuals: Capability*; Table 4; Table S7 in Multimedia Appendix 2). Among these, higher baseline positive, negative, or cognitive symptoms were associated with lower DMHI engagement. Qualitative data suggested that staff and patients anticipated such a pattern (and so may recommend or agree to DMHI use accordingly), and some patients avoided DMHIs due to experiencing difficult interactions between symptoms and technology (eg, technology-related delusions or paranoia). Findings for functioning and depression were more mixed, with higher baseline functioning or depression sometimes predicting higher and sometimes lower DMHI engagement. Qualitative studies suggested that while symptoms of depression (eg, poor concentration and motivation) may interfere with DMHI engagement, low mood may prompt higher engagement for some “because of a desire to find meanings and solutions for their depressive symptoms” [72]. No quantitative studies examined the effects of relapse on engagement; qualitative studies noted decreased DMHI use during relapse; however, participants usually restarted DMHIs on remission. Some were hesitant to (accurately) report symptoms via DMHIs for fear it may lead to more restrictive treatment (hospital admission and increased medication).

Negative interactions between DMHIs and symptoms, or perceived risk of such interactions, emerged as possible motivational barriers. Patients sometimes experienced DMHIs as “deflating, confronting, or triggering,” reminding them how unwell they were, or about their diagnosis, which could be disheartening, “self-stigmatizing,” and often prompted disengagement (*Individuals: Motivation*). There were numerous quotations about this, across 10 studies; however, it was difficult to tell how many participants were typically affected. While 1 study reported that half the sample felt negatively influenced by logging their moods and symptoms 5 times per day [157], in other studies with less intensive report schedules, very few (1/27, 4%) participants found the DMHI to be confronting [88]. Relatedly, fear of relapse was a barrier to DMHI use. Staff and patients anticipated that, by increasing awareness of symptoms, DMHIs would heighten users’ anxiety about deterioration, which may worsen symptoms. In studies testing actual DMHI use, there was qualitative [45,66,88,102] and quantitative [126] evidence that fear of relapse and increased rumination about symptoms prevented DMHI engagement but the proportion of participants affected was unclear. Reviewed papers did not typically report such negative reactions to DMHI use (ie, finding the DMHI “confronting,” or experiencing fear of relapse) as adverse reactions in the context of formal AE monitoring (Multimedia Appendix 2). Arguably, doing so would allow a quantitative examination of how many participants were affected across studies.

### Crosscutting Theme 5: Financial Provision for Implementing DMHIs

There was scant empirical information across CFIR constructs about the effects of financial provision on DMHI implementation, probably because most studies examined DMHI use in research studies rather than services. However, findings implied that financial limitations concerning software development and maintenance may indirectly reduce engagement. One study noted that some participant-suggested technical improvements were not possible due to financial limitations [107]. Another noted that the look and feel of the study app were considered “old school” but insufficient funding precluded improvements [109] (*Innovation: Cost*; Table 1); study authors highlighted the challenge of competing with commercial apps: “It is difficult to obtain... funding at a level that can compete with the funding that goes into app design for functions that people routinely use... leaving [study app] looking ‘old school’” [109]. Others noted that, while patients prefer free-to-use DMHIs (*Outer setting: Financing;* Table 2), sustainable business models for maintaining evidence-based DMHIs are often absent: “...apps that have been empirically evaluated and developed in accordance with clinical guidelines are rarely made available via the app marketplace... Thoughtful plans for commercialization and fiscal sustainability are important, given respondents with BD endorsed free/low-cost apps” [47]. This statement was supported by a striking absence of information from other reviewed studies about their intended model for DMHI dissemination and implementation, except a US-based study which mentioned costs were offset by the service’s billable nature [110] and a Canadian study that anticipated care provider networks would purchase DMHI licenses [109].

Some studies (5/150, 3.3%) listed costs associated with delivering DMHIs in the research context, including web hosting, software licensing fees, texting fees, technical support, and staff time for DMHI delivery. Financial provision for these and other DMHI-associated costs will be key to scaling up DMHI use in services but was not directly discussed in reviewed studies. Nevertheless, to maximize engagement with DMHIs for psychosis or bipolar disorder per the cross-cutting themes outlined above, considerable financial support would be necessary, particularly to provide human support for DMHI use (theme 2), to source or develop well-designed DMHIs that are as low-effort as possible for users (theme 3), and to address practical needs, such as digital poverty (theme 3).

## Discussion

### Principal Results

This systematic review found that patient and staff engagement with DMHIs is likely to be strengthened by using DMHIs to meet specific needs, not replace human care; providing human support for DMHIs and considering compatibility with current workflows; making DMHIs as low effort as possible for patients and staff, inquiring about common practical barriers to use (eg, digital poverty or training needs) and addressing these where possible; acknowledging interactions between symptoms and DMHIs, including motivational barriers and fear of relapse; and ensuring appropriate financial provision to implement DMHIs in line with these recommendations.

### Comparison With Prior Work

Similar to the review by Aref-Adib et al [17] on DMHIs for psychosis or bipolar disorder, most data were coded into CFIR *Intervention* (149/175, 85.1% papers) or *Individuals* (137/175, 78.3% papers) domains, with very little covering *Outer setting* (9/175, 5.1% papers) or *Implementation process* (5/175, 2.9% papers). We found more data related to the *Inner setting* (20/175, 11.4% papers) than the previous review, with studies highlighting a lack of compatibility with clinical workflows (17/175, 9.7% papers) and lack of alignment with staff perceptions of their clinical “mission” (5/175, 2.9% papers) as important barriers to DMHI implementation. This difference appears to reflect progress in the psychosis and bipolar disorder DMHI literature over the intervening years (Figure 1). Workflow-related barriers are mentioned in reviews covering DMHIs for common mental health disorders [21,158,159], a literature which has a chronological head-start on the equivalent literature for psychosis and bipolar disorder.

Our finding that human support was a key facilitator of DMHI engagement is consistent with previous reviews of factors affecting the implementation of DMHIs for psychosis or bipolar disorder [17,160], common mental health problems [11,21,158,161,162], and a meta-synthesis of staff views [158]. Relatedly, our review and others reported that clinicians’ negative attitudes to DMHIs, resistance to change, and lack of digital skills were sometimes barriers to DMHI implementation [17,158,163]. Nevertheless, such considerations are likely eclipsed by a need for compatibility with clinical workflows (which our findings suggest is currently absent; see *Inner setting: Compatibility*). Restructuring services to include new “digital navigator” roles, alongside traditional clinical roles, has been suggested as a model for implementing DMHIs in mental health services [10,164], and meta-analysis shows similar effectiveness across DMHIs guided by clinicians and nonclinicians [165]. Whether this model or any other service model is used, staff members (clinicians and nonclinicians) would need allocated time, training, and supervision to support patients’ DMHI use [158], all of which have financial implications [166].

DMHIs are frequently cited as potential methods of extending care in a cost-effective manner [167]. There is some empirical evidence supporting this, albeit from DMHIs tested in a research context [168], but economic evaluations of mental health DMHIs are rare [169]. Nevertheless, up-front costs of restructuring services and ongoing costs of delivering DMHIs will likely be substantial [17]. Few reviewed papers (5/175, 2.9%) directly evaluated financial factors, but our findings highlight the importance of considering staff-related costs and costs of addressing engagement barriers (eg, dated-looking digital interfaces; patients lacking suitable devices or skills). Without financial investment, DMHI engagement will likely be poor, potentially perpetuating existing treatment disparities [170,171]. More broadly, reviewed studies highlighted the difficulties of developing evidence-based DMHIs against a background of rapid technological advances and attractive but poorly evidenced commercial apps. Hill et al [172] outlined similar issues in relation to developing evidence-based DMHIs for common mental health problems; they recommended that researchers collaborate with industry partners to commercialize well-evidence DMHIs, generating funds to cover ongoing maintenance and development, and enhancing long-term sustainability.

We found that mental health symptoms and DMHIs sometimes interacted in ways that prevented DMHI engagement, which echoes and expands on findings from the review by Aref-Adib et al [17]. We found more evidence of motivational barriers relating to increased rumination or fear of relapse (26/175, 14.9% papers) than that found in the previous review (1/26, 4%). We also found that patients disengaged with DMHIs because they found increased self-reflection and attention toward negative emotional states too “confronting,” which has not been reported in previous reviews. Evidence from reviewed studies did not allow an estimate of how many patients are affected by these barriers. Future studies should consider examining such reactions quantitatively, perhaps as part of formal AE monitoring, to help future users make informed choices about DMHI risks and benefits. More generally, our supplementary analysis of safety reporting suggested that AE reporting warrants considerably more attention, in line with recent findings from other reviews [22,23,173] and recommendations regarding AE monitoring and reporting for DMHIs for psychosis [174].

### Strengths and Limitations of the Evidence Base

The reviewed literature contained a wealth of qualitative and quantitative data outlining factors that may affect staff and patient engagement with DMHIs for psychosis or bipolar disorder. However, the current evidence base has important limitations. First, most studies were conducted in traditional research settings rather than evaluating the process of implementing DMHIs directly in health services. Consequently, identified barriers and facilitators may not all translate and generalize to care provision settings. Nevertheless, the review provides an early indication of likely barriers and facilitators, across the CFIR domains, enabling these to be considered when designing future DMHIs and DMHI implementation strategies. This information will also be valuable in highlighting key implementation factors to prospectively examine in future implementation studies. Second, most studies seeking health professional views sought this information hypothetically (ie, they asked staff, who had not necessarily already used DMHIs, what they thought about using DMHIs in their clinical practice, in theory), whereas studies seeking information from patients more often asked about actual experiences of DMHI use (ie, they asked patients, who had already used a specific DMHI, about what it was like to use that DMHI). Berry et al [175] suggested that hypothetical views and actual experiences of DMHIs do not always align. Therefore, the difference in methods between patient and health professional studies must be kept in mind when considering the findings of this review.

### Strengths and Limitations of This Review

A key strength was the use of CFIR as a framework, allowing examination of interactions between staff and patient barriers and facilitators and wider contextual factors, and sensitizing us to implementation factors that may not otherwise have been identified in reviewed studies. Studies were identified using an inclusive search strategy, with intentionally wide inclusion criteria (eg, DMHI types, settings, and engagement indicators) to allow examination of a broad range of barriers and facilitators. However, the resulting studies were extremely heterogeneous, precluding meta-analysis and potentially conflating results from dissimilar studies. Likewise, the review covered a 21-year period (2010 to 2021), spanning a time of great technological advancement during which societal acceptance of DMHIs may have changed considerably, adding further heterogeneity. Finally, we limited our search to the period before the COVID-19 pandemic. Although this avoided COVID-19 pandemic–related heterogeneity, if the search had been conducted later, findings related to the impacts of the COVID-19 pandemic on digital uptake (*Outer setting: Critical incidents*) would likely have been prominent. We recommend that a future review should focus on mixed methods studies published since the pandemic, to examine the shifting views of staff and patients regarding potential barriers and facilitators of digital health tools for psychosis and bipolar disorder in the intervening period.

### Conclusions

While DMHIs have a role in supporting those with psychosis or bipolar disorder, intervention, individual, and contextual factors all impact uptake. To enhance engagement, DMHIs must be simple, low effort, meet specific needs, and not be heralded as a replacement for face-to-face care. Human support can overcome engagement barriers related to motivation and practicalities of DMHI engagement. Financial provision is likely to be key to sustainable DMHI implementation.

## Appendix

Multimedia Appendix 1 PRISMA (Preferred Reporting Items for Systematic reviews and Meta-Analyses) checklist.

Multimedia Appendix 2 Systematic review search terms and inclusion and exclusion criteria, detailed analysis of adverse event reports, list of excluded studies, and supplementary tables detailing characteristics and quality of included studies.

## Abbreviations

AE: adverse event
cCBT: computerized cognitive behavior therapy
CFIR: Consolidated Framework for Implementation Research
CONSORT: Consolidated Standards of Reporting Trials
DMHI: digital mental health intervention
PRISMA: Preferred Reporting Items for Systematic Reviews and Meta-Analyses
RCT: randomized controlled trial
