# Supplementary material for: Barriers and Facilitators of User Engagement With Digital Mental Health Interventions for People With Psychosis or Bipolar Disorder: Systematic Review and Best-Fit Framework Synthesis
Source: JMIR Ment Health. 2025 Jan 20;12:e65246. doi: 10.2196/65246 (PMC11791459; doi:10.2196/65246)
Supplement: Multimedia Appendix 2 [file mental_v12i1e65246_app2.docx]

**Multimedia Appendix 1: Supplementary Materials**

Manuscript title: Barriers and facilitators of user engagement with digital mental health interventions for people with psychosis or bipolar disorder: systematic review and best-fit framework synthesis

Contents

[Supplementary Methods SM1. Search terms and database list. 2](#_Toc185516937)

[Supplementary Methods SM2. PICOS inclusion/exclusion criteria (participants, interventions, comparators, outcomes, study design). 6](#_Toc185516938)

[Supplementary Results SR1. Analysis of adverse events reports 8](#_Toc185516939)

[Supplementary Table S1. Excluded studies and reasons for exclusion from main review 9](#_Toc185516940)

[Supplementary Table S2. Study design and details of digital mental health interventions. 28](#_Toc185516941)

[Supplementary Table S3. Sample characteristics of reviewed studies 44](#_Toc185516942)

[Supplementary Table S4. Mixed Methods Appraisal Tool (MMAT) Quality checks 55](#_Toc185516943)

[Supplementary Table S5. CONSORT harms checklist 60](#_Toc185516944)

[Supplementary Table S6. Quantitative associations between demographic variables and digital health intervention engagement in reviewed studies 69](#_Toc185516945)

[Supplementary Table S7. Associations between clinical variables and digital health intervention engagement in reviewed studies 71](#_Toc185516946)

[Supplementary Table S8. Reference list of studies included in the main review. 73](#_Toc185516947)

## Supplementary Methods SM1. Search terms and database list.

***Overview of databases searched***

Articles were identified through a PRISMA-compliant search of seven databases (Cochrane Central Register of Controlled Trials, MEDLINE, PsychInfo, PsychArticles, Embase, PubMed, Web of Science), conducted on 11/10/21. The search strategy combined search terms relating to severe mental health problems, digital mental health tools and barriers/facilitators/implementation. Searches were restricted to peer-reviewed articles published in English between January 2010 and October 2021 with human participants.

***OVID search (CCRCT, MEDLINE, PsychInfo, PsychArticles, Embase)***

(schizophren* or schizo?affective or psychotic or psychos?s or paranoia or paranoid or hallucination* or delusion* or bipolar or SMI or serious mental illness or severe mental illness or manic or mania or grandios* or serious mental health or severe mental health or EIS or Early intervention service? or Early intervention team? or early intervention program? or CMHT? or community mental health team? or assertive outreach team? or AOT? or home treatment team? or home based treatment team? or crisis team?).ti,kw,ab.

AND

(digital or m?health or e?health or phone? or smart?phone? or mobile? or app? or application? or wearable? or online or internet or cyber or web or mtherap* or etherap* or Technology or technologies or technological or webpage or website or smart?watch or smart?watches or AI or artificial intelligence or machine learning or smart?device? or digital phenotyping or digital phenotype? or sensing or passive data or passive monitoring or GPS or global positioning system or gyroscope? or accelerometer?).ti,kw,ab.

AND

(barrier? or facilitator? or enabler? or obstacle or implement* or engage* or adhere* or satisfaction or acceptability or usability or user experience? or satisfaction or usage or adoption or user perspective? or qualitative or interview or focus group or feasibility or Co-design).ti,kw,ab.

Wildcards: ‘?’ and ‘*’ are wildcards representing one (‘?’) or more (‘*’) characters, respectively

Search limits: English language, human, 2010-present, peer-reviewed articles, not reviews

Number of references retrieved**:** 3090

***PubMed search***

(schizophrenia[Title/Abstract] OR schizophrenic[Title/Abstract] OR schizoaffective[Title/Abstract] OR psychotic[Title/Abstract] OR psychosis[Title/Abstract] OR psychoses[Title/Abstract] OR paranoia[Title/Abstract] OR paranoid[Title/Abstract] OR hallucination[Title/Abstract] OR delusion[Title/Abstract] OR hallucinations[Title/Abstract] OR delusions[Title/Abstract] OR delusional[Title/Abstract] OR hallucinatory[Title/Abstract] OR bipolar[Title/Abstract] OR SMI[Title/Abstract] OR serious mental illness[Title/Abstract] OR severe mental illness[Title/Abstract] OR manic[Title/Abstract] OR mania[Title/Abstract] OR grandiose[Title/Abstract] OR grandiosity[Title/Abstract] OR serious mental health[Title/Abstract] OR severe mental health[Title/Abstract] OR EIS[Title/Abstract] OR Early intervention service[Title/Abstract] OR Early intervention team[Title/Abstract] OR early intervention program[Title/Abstract] OR Early intervention services[Title/Abstract] OR Early intervention teams[Title/Abstract] OR early intervention programs[Title/Abstract] OR CMHT[Title/Abstract] OR CMHTs[Title/Abstract] OR community mental health team[Title/Abstract] OR community mental health teams[Title/Abstract] OR AOT[Title/Abstract] OR AOTs[Title/Abstract] OR assertive outreach team[Title/Abstract] OR assertive outreach teams[Title/Abstract] OR home treatment team[Title/Abstract] OR home treatment teams[Title/Abstract] OR home based treatment team[Title/Abstract] OR home based treatment teams[Title/Abstract] OR crisis team[Title/Abstract] OR crisis teams[Title/Abstract])

AND

(digital[Title/Abstract] OR mhealth[Title/Abstract] OR ehealth[Title/Abstract] OR m-health[Title/Abstract] OR e-health[Title/Abstract] OR phone[Title/Abstract] OR smartphone[Title/Abstract] OR mobile[Title/Abstract] OR app[Title/Abstract] OR application[Title/Abstract] OR wearable[Title/Abstract] OR phones[Title/Abstract] OR smartphones[Title/Abstract] OR mobiles[Title/Abstract] OR apps[Title/Abstract] OR applications[Title/Abstract] OR wearables[Title/Abstract] OR online[Title/Abstract] OR internet[Title/Abstract] OR cyber[Title/Abstract] OR web[Title/Abstract] OR mtherapy[Title/Abstract] OR etherapy[Title/Abstract] OR m-therapy[Title/Abstract] OR e-therapy[Title/Abstract] OR m-therapies[Title/Abstract] OR e-therapies[Title/Abstract] OR mtherapies[Title/Abstract] OR etherapies[Title/Abstract] OR Technology[Title/Abstract] OR technologies[Title/Abstract] OR technological[Title/Abstract] OR webpage[Title/Abstract] OR website[Title/Abstract] OR smartwatch[Title/Abstract] OR smart-watch[Title/Abstract] OR smart watch[Title/Abstract] OR webpages[Title/Abstract] OR websites[Title/Abstract] OR smartwatches[Title/Abstract] OR smart-watches[Title/Abstract] OR smart watches[Title/Abstract] OR AI[Title/Abstract] OR artificial intelligence[Title/Abstract] OR machine learning[Title/Abstract] OR smartdevice[Title/Abstract] OR smart device[Title/Abstract] OR digital phenotyping[Title/Abstract] OR digital phenotype[Title/Abstract] OR digital phenotypes[Title/Abstract] OR sensing[Title/Abstract] OR passive data[Title/Abstract] OR passive monitoring[Title/Abstract] OR GPS[Title/Abstract] OR Global Positioning System[Title/Abstract] OR gyroscope[Title/Abstract] OR gyroscopes[Title/Abstract] OR accelerometer[Title/Abstract] OR accelerometers[Title/Abstract])

AND

(barrier[Title/Abstract] OR facilitator[Title/Abstract] OR obstacle[Title/Abstract] OR enabler[Title/Abstract] barriers[Title/Abstract] OR facilitators[Title/Abstract] OR obstacles[Title/Abstract] OR enablers[Title/Abstract] OR implement[Title/Abstract] OR implementation[Title/Abstract] OR engage[Title/Abstract] OR engagement[Title/Abstract] OR engagements[Title/Abstract] OR adhere[Title/Abstract] OR adherent[Title/Abstract] OR adherence[Title/Abstract] OR satisfaction[Title/Abstract] OR acceptability[Title/Abstract] OR usability[Title/Abstract] OR user experience[Title/Abstract] OR user experiences[Title/Abstract] OR satisfaction[Title/Abstract] OR usage[Title/Abstract] OR adoption[Title/Abstract] OR user perspective[Title/Abstract] OR user perspectives[Title/Abstract] OR qualitative[Title/Abstract] OR interview[Title/Abstract] OR focus group[Title/Abstract] OR feasibility[Title/Abstract] OR Co-design[Title/Abstract] OR interviews[Title/Abstract] OR focus groups[Title/Abstract])

Wildcards: none

Search limits: English language, human, 2010-present

Number of references retrieved**:** 1489

***Web of science search terms***

TI=(“schizophren*”) OR TI=(“schizo$affective”) OR TI=(“psychotic”) OR TI=(“psychos$s”) OR TI=(“paranoia”) OR TI=(“paranoid”) OR TI=(“hallucination*”) OR TI=(“delusion*”) OR TI=(“bipolar”) OR TI=(“SMI”) OR TI=(“serious mental illness”) OR TI=(“severe mental illness”) OR TI=(“manic”) OR TI=(“mania”) OR TI=(“grandios*”) OR TI=(“serious mental health”) OR TI=(“severe mental health”) OR TI=(“EIS”) OR TI=(“Early intervention service$”) OR TI=(“Early intervention team$”) OR TI=(“early intervention program$”) OR TI=(“CMHT$”) OR TI=(“community mental health team$”) OR TI=(“assertive outreach team$”) OR TI=(“AOT$”) OR TI=(“home treatment team$”) OR TI=(“home based treatment team$”) OR TI=(“crisis team$”) OR AB=(“schizophren*”) OR AB=(“schizo$affective”) OR AB=(“psychotic”) OR AB=(“psychos$s”) OR AB=(“paranoia”) OR AB=(“paranoid”) OR AB=(“hallucination*”) OR AB=(“delusion*”) OR AB=(“bipolar”) OR AB=(“SMI”) OR AB=(“serious mental illness”) OR AB=(“severe mental illness”) OR AB=(“manic”) OR AB=(“mania”) OR AB=(“grandios*”) OR AB=(“serious mental health”) OR AB=(“severe mental health”) OR AB=(“EIS”) OR AB=(“Early intervention service$”) OR AB=(“Early intervention team$”) OR AB=(“early intervention program$”) OR AB=(“CMHT$”) OR AB=(“community mental health team$”) OR AB=(“assertive outreach team$”) OR AB=(“AOT$”) OR AB=(“home treatment team$”) OR AB=(“home based treatment team$”) OR AB=(“crisis team$”) OR AK=(“schizophren*”) OR AK=(“schizo$affective”) OR AK=(“psychotic”) OR AK=(“psychos$s”) OR AK=(“paranoia”) OR AK=(“paranoid”) OR AK=(“hallucination*”) OR AK=(“delusion*”) OR AK=(“bipolar”) OR AK=(“SMI”) OR AK=(“serious mental illness”) OR AK=(“severe mental illness”) OR AK=(“manic”) OR AK=(“mania”) OR AK=(“grandios*”) OR AK=(“serious mental health”) OR AK=(“severe mental health”) OR AK=(“EIS”) OR AK=(“Early intervention service$”) OR AK=(“Early intervention team$”) OR AK=(“early intervention program$”) OR AK=(“CMHT$”) OR AK=(“community mental health team$”) OR AK=(“assertive outreach team$”) OR AK=(“AOT$”) OR AK=(“home treatment team$”) OR AK=(“home based treatment team$”) OR AK=(“crisis team$”)

AND

TI=(“digital”) OR TI=(“m$health”) OR TI=(“e$health”) OR TI=(“phone$”) OR TI=(“smart$phone”) OR TI=(“smart$phones”) OR TI=(“mobile$”) OR TI=(“app$”) OR TI=(“application$”) OR TI=(“wearable$”) OR TI=(“online”) OR TI=(“internet”) OR TI=(“cyber”) OR TI=(“web”) OR TI=(“mtherap*”) OR TI=(“etherap*”) OR TI=(“Technology”) OR TI=(“technologies”) OR TI=(“technological”) OR TI=(“webpage”) OR TI=(“website”) OR TI=(“smart$watch”) OR TI=(“smart$watches”) OR TI=(“AI”) OR TI=(“artificial intelligence”) OR TI=(“machine learning”) OR TI=(“smart$device”) OR TI=(“smart$devices”) OR TI=(“digital phenotyping”) OR TI=(“digital phenotype$”) OR TI=(“sensing”) OR TI=(“passive data”) OR TI=(“passive monitoring”) OR TI=(“GPS”) OR TI=(“global positioning system”) OR TI=(“gyroscope$”) OR TI=(“accelerometer$”) OR AB=(“digital”) OR AB=(“m$health”) OR AB=(“e$health”) OR AB=(“phone$”) OR AB=(“smart$phone”) OR AB=(“smart$phones”) OR AB=(“mobile$”) OR AB=(“app$”) OR AB=(“application$”) OR AB=(“wearable$”) OR AB=(“online”) OR AB=(“internet”) OR AB=(“cyber”) OR AB=(“web”) OR AB=(“mtherap*”) OR AB=(“etherap*”) OR AB=(“Technology”) OR AB=(“technologies”) OR AB=(“technological”) OR AB=(“webpage”) OR AB=(“website”) OR AB=(“smart$watch”) OR AB=(“smart$watches”) OR AB=(“AI”) OR AB=(“artificial intelligence”) OR AB=(“machine learning”) OR AB=(“smart$device”) OR AB=(“smart$devices”) OR AB=(“digital phenotyping”) OR AB=(“digital phenotype$”) OR AB=(“sensing”) OR AB=(“passive data”) OR AB=(“passive monitoring”) OR AB=(“GPS”) OR AB=(“global positioning system”) OR AB=(“gyroscope$”) OR AB=(“accelerometer$”) OR AK=(“digital”) OR AK=(“m$health”) OR AK=(“e$health”) OR AK=(“phone$”) OR AK=(“smart$phone”) OR AK=(“smart$phones”) OR AK=(“mobile$”) OR AK=(“app$”) OR AK=(“application$”) OR AK=(“wearable$”) OR AK=(“online”) OR AK=(“internet”) OR AK=(“cyber”) OR AK=(“web”) OR AK=(“mtherap*”) OR AK=(“etherap*”) OR AK=(“Technology”) OR AK=(“technologies”) OR AK=(“technological”) OR AK=(“webpage”) OR AK=(“website”) OR AK=(“smart$watch”) OR AK=(“smart$watches”) OR AK=(“AI”) OR AK=(“artificial intelligence”) OR AK=(“machine learning”) OR AK=(“smart$device”) OR AK=(“smart$devices”) OR AK=(“digital phenotyping”) OR AK=(“digital phenotype$”) OR AK=(“sensing”) OR AK=(“passive data”) OR AK=(“passive monitoring”) OR AK=(“GPS”) OR AK=(“global positioning system”) OR AK=(“gyroscope$”) OR AK=(“accelerometer$”)

AND

TI=(“barrier$”) OR TI=(“facilitator$”) OR TI=(“enabler$”) OR TI=(“obstacle”) OR TI=(“implement*”) OR TI=(“engage*”) OR TI=(“adhere*”) OR TI=(“satisfaction”) OR TI=(“acceptability”) OR TI=(“usability”) OR TI=(“user experience$”) OR TI=(“satisfaction”) OR TI=(“usage”) OR TI=(“adoption”) OR TI=(“user perspective$”) OR TI=(“qualitative”) OR TI=(“interview”) OR TI=(“focus group”) OR TI=(“feasibility”) OR TI=(“Co-design”) OR AB=(“barrier$”) OR AB=(“facilitator$”) OR AB=(“enabler$”) OR AB=(“obstacle”) OR AB=(“implement*”) OR AB=(“engage*”) OR AB=(“adhere*”) OR AB=(“satisfaction”) OR AB=(“acceptability”) OR AB=(“usability”) OR AB=(“user experience$”) OR AB=(“satisfaction”) OR AB=(“usage”) OR AB=(“adoption”) OR AB=(“user perspective$”) OR AB=(“qualitative”) OR AB=(“interview”) OR AB=(“focus group”) OR AB=(“feasibility”) OR AB=(“Co-design”) OR AK=(“barrier$”) OR AK=(“facilitator$”) OR AK=(“enabler$”) OR AK=(“obstacle”) OR AK=(“implement*”) OR AK=(“engage*”) OR AK=(“adhere*”) OR AK=(“satisfaction”) OR AK=(“acceptability”) OR AK=(“usability”) OR AK=(“user experience$”) OR AK=(“satisfaction”) OR AK=(“usage”) OR AK=(“adoption”) OR AK=(“user perspective$”) OR AK=(“qualitative”) OR AK=(“interview”) OR AK=(“focus group”) OR AK=(“feasibility”) OR AK=(“Co-design”)

Wildcards: ‘*’, ‘?’ and ‘$’ are wildcards representing any group of characters (*), any single character (?), and zero or one character ($), respectively.

Search limits: English language, 2010-present, not reviews.

Number of references retrieved: 4531

## Supplementary Methods SM2. PICOS inclusion/exclusion criteria (participants, interventions, comparators, outcomes, study design).

***Participants***

Included:

- People aged 16 or over with a schizophrenia spectrum diagnosis (e.g., schizophrenia, schizoaffective, psychosis not otherwise specified, first episode psychosis) or bipolar disorder diagnosis.
- Mental health professionals who work with people aged 16 or over with a schizophrenia spectrum diagnosis or bipolar disorder diagnosis.

Excluded:

- Studies with mixed samples in which less than half of the participants meet inclusion criteria.

***Interventions***

Included:

- Delivered by smartphone app, text messaging, online/website or wearable device.
- Digital health tool which aims to monitor and/or improve the mental and/or physical health of people with psychosis/bipolar. Can include (not exhaustive):
  - Active symptom monitoring
  - Passive monitoring/sensing
  - Cognitive remediation delivered digitally
  - CBT delivered digitally
  - Medication adherence interventions/monitoring
  - Smoking interventions/monitoring
  - Exercise interventions/monitoring
  - Self-harm interventions/monitoring
  - Substance use interventions/monitoring
  - Sleep interventions/monitoring
  - Digital pills
  - EMI – ecological momentary intervention (but not EMA/ESM if it is purely for research)
- The study can be testing actual use of a digital mental health tool or can be hypothetical (i.e., asking people what they think about a digital mental health tool in theory)

Excluded:

- Digital tools that are used as a component during an in-person session but cannot be used remotely outside of these sessions.
- Digital mental health tools for families/carers of people with psychosis/bipolar.
- Digital mental health tools for mental health staff themselves.
- Intervention/monitoring delivered via video-conferencing or phone calls only.
- Digital tools that serve only as an appointment booking system for in-person therapy.
- Electronic health records that only health professionals (not service users) can view and contribute to
- Screening apps.
- Interventions delivered by virtual reality (VR)^[[1]](#footnote-2)^.
- Experience Sampling Methodology (ESM), Ecological Momentary Assessment (EMA) or other digital monitoring studies where ESM/EMA is used purely as a research tool (e.g. ESM investigating the association between X and Y) as we are interested in potential clinical use, not purely research use.
- Interventions/monitoring where the only device used is a pedometer

***Comparators***

No comparison group needed.

***Outcomes***

Included

- Must examine factors affecting user engagement with a digital mental health tool (not necessarily as the primary research focus).
  - These may be examined qualitatively or quantitatively.
  - User engagement can be measured by any User Engagement Indicator (UEI) listed in Ng and colleagues’ (2019)^[[2]](#footnote-3)^ review. UEIs include subjective criteria (interview or survey) and objective criteria (usage data) variously examining usage, usability, user satisfaction, acceptability or feasibility.

***Study design***

Included:

- Peer reviewed studies
- Qualitative, quantitative, or mixed methods studies

Excluded:

- Grey literature
- Conference abstracts
- Dissertations or theses
- Protocols
- Studies in languages other than English
- Non-empirical studies: e.g., literature reviews, commentary papers, opinion pieces, editorials
- Letters to the editor

## Supplementary Results SR1. Analysis of adverse events reports

There were 168 papers reporting on 139 studies including actual use of a digital tool (as opposed to hypothetical discussion or usability testing in session) from which adverse events (AE) information was extracted. Seventy-six studies reported some content related to AEs in one or more of their outputs, and 86 papers in total reported some content related to AEs. However, in only 23 cases was there actual numerical or percentage-based reporting of AEs. These papers reported AEs such as hospitalisations, psychological distress, suicidal ideation, occasionally physical health related events, and occasionally death. Relatedness to the intervention was only reported in 11 of these papers; in 8 of these some events were deemed related.

Some papers reported only SAEs (n=4 papers), or discussed only related events/adverse *effects*, not all AEs (including those which might not be related) (n=32). Some studies quantitatively reported some undesirable outcomes, experiences or events, but did not mention is these were classed as AEs (n=25 papers) and sometimes events which might be classified as AEs (e.g., hospitalisation, death, relapse) were being measured as outcomes of studies, so were reported as such, and not as AEs (n=19 papers). Reporting of AE data was most commonly in the findings, although it was reported only in the appendix in six cases, and in three cases was first introduced in the discussion section without reference to data supporting conclusions.

Some papers qualitatively examined and reported events which might be classified as AEs but were not reported as such (n=30). Within this sub-group, most examined only related events (n=18 of 30). These studies often asked participants about negative effects of use of the digital tool, and some common responses included that use of tools was irritating, frustrating, prompts “felt like nagging”, or that mood or symptom monitoring could cause undesired self-reflection or increased attention toward negative emotional states. Occasionally, participants reported worry related to privacy, fear of monitoring, or fear of being hospitalised if symptoms reported through a digital tool were interpreted as relapse. The mechanisms via which some commonly reported negative effects of DHIs might occur were the same as those responsible for some commonly reported benefits: increased self-monitoring could improve self-awareness or cause undesired self-reflection, and information being conveyed to others (such as the care team) could help detect and respond to change, or could be negative if privacy was a concern.

Supplementary Table ST7 examines to what extent each paper met criteria from the CONSORT harms checklist. Reporting was overall poor or lacking, which may or may not relate to poor actual assessment and monitoring of AEs. Very few articles reported use of a validated instrument to report AE severity (n=3), or definition of an AE (n=4), and few reported AEs and SAEs separately (n=6), whilst more (but still a minority) reported how and when harms data were collected (n=37, n=29).

## Supplementary Table S1. Excluded studies and reasons for exclusion from main review

**Note:** Although the 36 papers marked with an * in the first column were excluded from the main review, they were included in the supplementary review of safety reporting in studies testing actual DHI use (Supplementary Results SR1; Supplementary Table S5).

| ***#*** | **Study** | **Primary reason for exclusion from main review** |
| --- | --- | --- |
| 1 | Busk J, Faurholt-Jepsen M, Frost M, Bardram JE, Vedel Kessing L, Winther O. Forecasting Mood in Bipolar Disorder From Smartphone Self-assessments: Hierarchical Bayesian Approach. JMIR mHealth and uHealth. 2020;8(4):e15028. | No data on factors affecting user engagement |
| 2 | Minami H, Brinkman HR, Rivera-Mindt M, Vieira C, Donnelly R, McClain LM, et al. Rationale, design and pilot feasibility results of a smartphone-assisted, mindfulness-based intervention for smokers with mood disorders: Project mSMART MIND. Contemporary Clinical Trials. 2018;66:36-44. | Sample <50% SMI or MH professionals |
| 3 | Poole R, Smith D, Simpson S. How Patients Contribute to an Online Psychoeducation Forum for Bipolar Disorder: A Virtual Participant Observation Study. JMIR MENTAL HEALTH. 2015;2(3). | No data on factors affecting user engagement |
| 5 | Murray G, Leitan N, Berk M, Thomas N, Michalak E, Berk L, et al. The orbit project: pilot evidence for feasibility and efficacy of a novel international online mindfulness-based intervention for late stage bipolar disorder. BIPOLAR DISORDERS. 2015;17:54-. | Not published in peer reviewed journal |
| 6 | Williams J, Stubbs B, Richardson S, Flower C, Barr-Hamilton L, Grey B, et al. 'Walk this way': results from a pilot randomised controlled trial of a health coaching intervention to reduce sedentary behaviour and increase physical activity in people with serious mental illness. BMC Psychiatry. 2019;19(1):287. | Study not about digital health tools |
| 7 | Vitger T, Petersen L, Korsbek L, Austin SF, Tonder ES, Nordentoft M. The Momentum trial: The efficacy of using a smartphone application to promote patient activation and support shared decision making in people with a diagnosis of schizophrenia in outpatient treatment settings: A randomized controlled single-blind trial. BMC Psychiatry. 2019;19(1) | No data on factors affecting user engagement |
| 9 | Valenza G, Nardelli M, Bertschy G, Lanatà A, Barbieri R, Scilingo EP. Maximal-radius multiscale entropy of cardiovascular variability: a promising biomarker of pathological mood states in bipolar disorders. Annu Int Conf IEEE Eng Med Biol Soc. 2014;2014:6663-6. | Not published in peer reviewed journal |
| 10 | Friehs MA, Dechant M, Vedress S, Frings C, Mandryk RL. Effective Gamification of the Stop-Signal Task: Two Controlled Laboratory Experiments. JMIR SERIOUS GAMES. 2020;8(4):257-77. | Sample <50% SMI or MH professionals |
| 12 | Peck CE, Lim MH, Purkiss M, Foley F, Hopkins L, Thomas N. Development of a Lived Experience-Based Digital Resource for a Digitally-Assisted Peer Support Program for Young People Experiencing Psychosis. FRONTIERS IN PSYCHIATRY. 2020;11. | Digital tool is purely video/phone calls, appointment system, EHR, or used only in person |
| 13 | Gitlow L, Abdelaal F, Etienne A, Hensley J, Krukowski E, Toner M. Exploring the Current Usage and Preferences for Everyday Technology among People with Serious Mental Illnesses. OCCUPATIONAL THERAPY IN MENTAL HEALTH. 2017;33(1):1-14. | Study not about digital health tools |
| 14* | Ben-Zeev D, Kaiser SM, Krzos I. Remote hovering with individuals with psychotic disorders and substance use: feasibility, engagement, and therapeutic alliance with a text-messaging mobile interventionist. J Dual Diagn. 2014;10(4):197-203. | No data on factors affecting user engagement |
| 15 | Dev R, Li J, Zhang D, Cai Y, Hao C, Hou F, et al. An economic evaluation of a mobile text messaging intervention to improve mental health care in resource-poor communities in China: a cost-effectiveness study. BMC Health Serv Res. 2020;20(1):989. | Not an empirical study |
| 16 | Skanderson M, Ramsey C, Abel EA, Erdos J, Shimada SL, Houston TK, et al. Dual Use of a Patient Portal and Clinical Video Telehealth by Veterans with Mental Health Diagnoses: Retrospective, Cross-Sectional Analysis. Journal of medical Internet research. 2018;20(11):e11350. | Sample <50% SMI or MH professionals |
| 18 | van de Mheen D, van Oers H, Buitenweg D, van Nieuwenhuizen C, Grund J-P. Visual and personalized quality of life assessment app for people with severe mental health problems: Qualitative evaluation. JMIR Mental Health. 2020;7(12):e19593. | Sample <50% SMI or MH professionals |
| 21 | Tasma, M., et al. (2018). The development and evaluation of a computerized decision aid for the treatment of psychotic disorders. BMC Psychiatry 18(1): 163. | Digital tool not delivered via app, texts, website or wearable |
| 25 | Moccia, A., et al. (2020). Cognitive training in virtual reality vs. cogpack in patients with schizophrenia: study proposal. Minerva Psichiatrica 61(1): 5-9. | Not an empirical study |
| 30 | Fletcher, K., et al. (2018). Web-Based Self-Management Programs for Bipolar Disorder: Insights From the Online, Recovery-Oriented Bipolar Individualised Tool Project. Journal of Medical Internet Research 20(10): e11160. | No data on factors affecting user engagement |
| 31 | Wang, X., et al. (2021). HOPES: An integrative digital phenotyping platform for data collection, monitoring, and machine learning. Journal of Medical Internet Research 23(3): e23984. | Digital tool not for monitoring/improving physical or mental health of people with SMI |
| 32 | Povey, J., et al. (2020). Drafting the Aboriginal and Islander Mental Health Initiative for Youth (AIMhi-Y) App: Results of a formative mixed methods study. Internet Interventions 21: 100318. | Participants are children under 16 |
| 33 | Wong, S. K., et al. (2014). Mobile phone text message reminders of antipsychotic medication: Is it time and who should receive them? A cross-sectional trust-wide survey of psychiatric inpatients. BMC Psychiatry 14(1): 15. | Sample <50% SMI or MH professionals |
| 34 | Cho, C.-H., et al. (2017). Design and methods of the mood disorder cohort research consortium (MDCRC) study. Psychiatry Investigation 14(1): 100-106. | Not an empirical study |
| 36 | Isleyen, F., et al. (2014). Evaluation of the usability of a serious game aiming to teach facial expressions to schizophrenic patients. Studies in health technology and informatics 205: 662-666. | No data on factors affecting user engagement |
| 37 | Knights, J., et al. (2019). Evaluating digital medicine ingestion data from seriously mentally ill patients with a Bayesian Hybrid Model. npj Digital Medicine 2. | No data on factors affecting user engagement |
| 38 | Vilardaga, R., et al. (2020). Pilot Randomized Controlled Trial of a Novel Smoking Cessation App Designed for Individuals With Co-Occurring Tobacco Use Disorder and Serious Mental Illness. Nicotine & tobacco research : official journal of the Society for Research on Nicotine and Tobacco 22(9): 1533-1542. | No data on factors affecting user engagement |
| 39 | Cosgrove, V., et al. (2018). User engagement within the moodswings 2.0 online self-guided intervention for bipolar disorder. Bipolar Disorders 20: 30-30. | Not published in peer reviewed journal |
| 40* | Kalinich, M., et al. (2021). Applying machine learning to smartphone based cognitive and sleep assessments in schizophrenia. Schizophrenia Research: Cognition: 100216. | No data on factors affecting user engagement |
| 41 | Abdullah, S., et al. (2016). Automatic detection of social rhythms in bipolar disorder. Journal of the American Medical Informatics Association 23(3): 538-543. | No data on factors affecting user engagement |
| 42* | Abel, D. B. and K. S. Minor (2021). Social functioning in schizophrenia: Comparing laboratory-based assessment with real-world measures. Journal of Psychiatric Research 138: 500-506. | No data on factors affecting user engagement |
| 43 | Abplanalp, S. J., et al. (2021). Feasibility of using smartphones to capture speech during social interactions in schizophrenia. Schizophr Res 228: 51-52. | Not published in peer reviewed journal |
| 44* | Adler, D. A., et al. (2020). Predicting Early Warning Signs of Psychotic Relapse From Passive Sensing Data: An Approach Using Encoder-Decoder Neural Networks. JMIR mHealth and uHealth 8(8): e19962. | No data on factors affecting user engagement |
| 46 | Alipour, A., et al. (2020). Ingestible sensors and medication adherence: Focus on use in serious mental illness. Pharmacy 8(2): 103. | Not an empirical study |
| 48 | Alonso-Solís, A., et al. (2020). A Method to Compare the Delivery of Psychiatric Care for People with Treatment-Resistant Schizophrenia. Int J Environ Res Public Health 17(20). | Sample <50% SMI or MH professionals |
| 49 | Alvarez-Jimenez, M., et al. (2013). On the HORYZON: Moderated online social therapy for long-term recovery in first episode psychosis. Schizophrenia Research 143(1): 143-149. | Sample <50% SMI or MH professionals |
| 51* | Amiri, A. M., et al. (2019). Predicting physical activity levels in individuals with schizophrenia through integrated global positioning system and accelerometer data. Schizophrenia Research 212: 246-248. | No data on factors affecting user engagement |
| 52 | Anonymous (2019). Aripiprazole with digital ingestion tracking (Abilify mycite). Medical Letter on Drugs and Therapeutics 61(1564): 15-16. | Not an empirical study |
| 53 | Anttila, M., et al. (2012). Use of web-based patient education sessions on psychiatric wards. International Journal of Medical Informatics 81(6): 424-433. | Digital tool is purely video/phone calls, appointment system, EHR, or used only in person |
| 54 | Arevian, A. C., et al. (2020). Clinical state tracking in serious mental illness through computational analysis of speech. PLoS ONE 15(1): e0225695. | Digital tool not delivered via app, texts, website or wearable |
| 58* | Aschbrenner, K. A., et al. (2016). A Qualitative Study of Client-Clinician Text Exchanges in a Mobile Health Intervention for Individuals With Psychotic Disorders and Substance Use. J Dual Diagn 12(1): 63-71. | No data on factors affecting user engagement |
| 59 | Aschbrenner, K. A., et al. (2019). Adolescents' Use of Digital Technologies and Preferences for Mobile Health Coaching in Public Mental Health Settings. Frontiers in public health 7. | Participants are children under 16 |
| 60 | Aschbrenner, K., et al. (2019). Randomized trial of a lifestyle intervention for young adults with serious mental illness in community mental health centers. Schizophrenia Bulletin 45(Supplement 2): S135. | Not published in peer reviewed journal |
| 61 | Asuzu, K. and M. Z. Rosenthal (2021). Mobile device use among inpatients on a psychiatric unit: A preliminary study. Psychiatry Research 297: 113720. | Sample <50% SMI or MH professionals |
| 63* | Bain, E. E., et al. (2017). Use of a Novel Artificial Intelligence Platform on Mobile Devices to Assess Dosing Compliance in a Phase 2 Clinical Trial in Subjects With Schizophrenia. JMIR mHealth and uHealth 5(2). | No data on factors affecting user engagement |
| 65 | Barrera, A., et al. (2020). Introducing artificial intelligence in acute psychiatric inpatient care: qualitative study of its use to conduct nursing observations. Evidence-based mental health 23(1): 34-38. | Study not about digital health tools |
| 67 | Bauer, A. M., et al. (2018). Applying the Principles for Digital Development: Case Study of a Smartphone App to Support Collaborative Care for Rural Patients With Posttraumatic Stress Disorder or Bipolar Disorder. Journal of Medical Internet Research 20(6): e10048. | Sample <50% SMI or MH professionals |
| 70 | Beard, C., et al. (2019). Smartphone, Social Media, and Mental Health App Use in an Acute Transdiagnostic Psychiatric Sample. JMIR mHealth and uHealth 7(6): e13364. | Sample <50% SMI or MH professionals |
| 72 | Becker, M. P., et al. (2016). Preferences for Early Intervention Mental Health Services: A Discrete-Choice Conjoint Experiment. Psychiatr Serv 67(2): 184-191. | No data on factors affecting user engagement |
| 73 | Becker, M., et al. (2019). Investigating service features to sustain engagement in early intervention mental health services. Early Interv Psychiatry 13(2): 241-250. | No data on factors affecting user engagement |
| 74 | Beebe, L. H., et al. (2010). Keeping in touch: Cell phone use in people with schizophrenia spectrum disorders. Journal of Psychosocial Nursing and Mental Health Services 48(4): 32-37. | Study not about digital health tools |
| 75 | Beentjes, T. A. A., et al. (2016). Development of an e-supported illness management and recovery programme for consumers with severe mental illness using intervention mapping, and design of an early cluster randomized controlled trial. BMC health services research 16: 20. | Not an empirical study |
| 79 | Ben-Zeev, D., et al. (2013). Mobile technologies in schizophrenia: Current use and readiness for mobile interventions. Schizophrenia Bulletin 39(SUPPL. 1): S111. | Not published in peer reviewed journal |
| 81 | Ben-Zeev, D., et al. (2014). SERVICES & POLICY. Journal of Dual Diagnosis 10(4): 197-203. | Not an empirical study |
| 82 | Ben-Zeev, D., et al. (2015). Strategies for mHealth Research: Lessons from 3 Mobile Intervention Studies. ADMINISTRATION AND POLICY IN MENTAL HEALTH AND MENTAL HEALTH SERVICES RESEARCH 42(2): 157-167. | Not an empirical study |
| 87 | Ben-Zeev, D., et al. (2020). Augmenting evidence-based care with a texting mobile interventionist: A pilot randomized controlled trial. Psychiatric Services 71(12): 1218-1224. | No data on factors affecting user engagement |
| 88 | Ben-Zeev, D., et al. (2021). A Digital Toolkit (M-Healer) to Improve Care and Reduce Human Rights Abuses Against People With Mental Illness in West Africa: User-Centered Design, Development, and Usability Study. JMIR Mental Health 8(7). | Sample <50% SMI or MH professionals |
| 90 | Ben-Zeev, D., et al. (2021). Cost of mHealth Versus Clinic-Based Care for Serious Mental Illness: Same Effects, Half the Price Tag. Psychiatric Services 72(4): 448-451. | No data on factors affecting user engagement |
| 96 | Biagianti, B., et al. (2017). CLIMB: A MOBILE INTERVENTION TO ENHANCE SOCIAL FUNCTIONING IN PEOPLE WITH PSYCHOTIC DISORDERS: RESULTS FROM A FEASIBILITY STUDY. Schizophrenia Bulletin 43: S210-S210. | Not published in peer reviewed journal |
| 97 | Bianco, C. L., et al. (2021). Can Smartphone Apps Assist People with Serious Mental Illness in Taking Medications as Prescribed? Sleep Medicine Clinics 16(1): 213-222. | Not an empirical study |
| 98 | Bidargaddi, N., et al. (2021). Demonstration of automated non-adherence and service disengagement risk monitoring with active follow-up for severe mental illness. Australian and New Zealand Journal of Psychiatry 55(10): 976-982. | Sample <50% SMI or MH professionals |
| 99 | Bilderbeck, A. C., et al. (2016). Psychoeducation and online mood tracking for patients with bipolar disorder: A randomised controlled trial. Journal of Affective Disorders 205: 245-251. | No data on factors affecting user engagement |
| 100 | Bird, J., et al. (2017). Diversity in eMental Health Practice: An Exploratory Qualitative Study of Aboriginal and Torres Strait Islander Service Providers. JMIR Mental Health 4(2). | Sample <50% SMI or MH professionals |
| 101 | Bonet, L., et al. (2018). Is internet harmful for psychotic patients? Schizophrenia Bulletin 44(Supplement 1): S424. | Not published in peer reviewed journal |
| 103 | Bonfils, K. A., et al. (2018). Implementing CommonGround in a Community Mental Health Center: Lessons in a Computerized Decision Support System. Psychiatric rehabilitation journal 41(3): 216-223. | Digital tool is purely video/phone calls, appointment system, EHR, or used only in person |
| 108 | Boydell, K., et al. (2017). Self-management in young adults with bipolar disorder: Strategies and challenges. Journal of Affective Disorders 209: 201-208. | Study not about digital health tools |
| 109 | Bradshaw, T., et al. (2012). Developing a healthy living intervention for people with early psychosis using the Medical Research Council's guidelines on complex interventions: Phase 1 of the HELPER - InterACT programme. International journal of nursing studies 49(4): 398-406. | Study not about digital health tools |
| 110 | Browne, J., et al. (2021). Engagement with a digital therapeutic for smoking cessation designed for persons with psychiatric illness fully mediates smoking outcomes in a pilot randomized controlled trial. Translational Behavioral Medicine. | No data on factors affecting user engagement |
| 111 | Brucker, D. L. and M. Doty (2019). Community mental health center staff attitudes about employment for persons with serious mental illness. Psychiatric rehabilitation journal 42(1): 32-40. | Study not about digital health tools |
| 113 | Bruins, J., et al. (2020). Qualitative analysis of clinicians' perspectives on the use of a computerized decision aid in the treatment of psychotic disorders. BMC medical informatics and decision making 20(1): 234. | Digital tool not delivered via app, texts, website or wearable |
| 114 | Brunette, M. (2012). Smoking cessation websites do not meet usability guidelines or content needs of people with schizophrenia. American Journal on Addictions 21(4): 382-383. | Not published in peer reviewed journal |
| 115 | Brunette, M. F., et al. (2016). Coordinated technology-delivered treatment to prevent rehospitalization in schizophrenia: A novel model of care. Psychiatric Services 67(4): 444-447. | Not an empirical study |
| 116 | Brunette, M. F., et al. (2017). Use of computers and Internet among people with severe mental illnesses at peer support centers. Psychiatric rehabilitation journal 40(4): 405-408. | Sample <50% SMI or MH professionals |
| 118 | Brusilovskiy, E., et al. (2016). Social media use, community participation and psychological well-being among individuals with serious mental illnesses. Computers in Human Behavior 65: 232-240. | Study not about digital health tools |
| 119 | Brusilovskiy, E., et al. (2020). Examining the relationship between community mobility and participation using GPS and self-report data. Social Science and Medicine 265: 113539. | Digital tool not for monitoring/improving physical or mental health of people with SMI |
| 123 | Budiarto, E., et al. (2021). The Effect of Online Acceptance and Commitment Therapy and Family Psychoeducation Therapy on Personal and Social Performances and Treatment Compliance for Schizophrenia Clients with the Risk of Behavioral Violence and Hallucinations in Community. PAKISTAN JOURNAL OF MEDICAL & HEALTH SCIENCES 15(1): 292-300. | Study not about digital health tools |
| 124 | Buitenweg, D. C., et al. (2019). Cocreative Development of the QoL-ME: A Visual and Personalized Quality of Life Assessment App for People With Severe Mental Health Problems. JMIR Mental Health 6(3). | Sample <50% SMI or MH professionals |
| 125 | Busk, J., et al. (2020). Daily estimates of clinical severity of symptoms in bipolar disorder from smartphone-based self-assessments. Translational Psychiatry 10(1): 194. | No data on factors affecting user engagement |
| 127 | Campos, C., et al. (2015). Feasibility and acceptability of an exergame intervention for schizophrenia. Psychology of Sport and Exercise 19: 50-58. | Digital tool is purely video/phone calls, appointment system, EHR, or used only in person |
| 129 | Carpiniello, B., et al. (2020). Challenges and future prospects of precision medicine in psychiatry. Pharmacogenomics and Personalized Medicine 13: 127-140. | Not an empirical study |
| 131* | Cella, M., et al. (2018). Using wearable technology to detect the autonomic signature of illness severity in schizophrenia. Schizophrenia Research 195: 537-542. | No data on factors affecting user engagement |
| 132* | Cella, M., et al. (2019). Blending active and passive digital technology methods to improve symptom monitoring in early psychosis. Early Interv Psychiatry 13(5): 1271-1275. | No data on factors affecting user engagement |
| 133 | Chamberlain, D. (2013). Tracking tactics: can a digital tablet help with medication adherence in schizophrenia? NEUROPSYCHIATRY 3(5): 461-461. | Not an empirical study |
| 134 | Chapman, J. J., et al. (2017). Quantification of free-living activity patterns using accelerometry in adults with mental illness. Scientific reports 7: 43174. | Digital tool not for monitoring/improving physical or mental health of people with SMI |
| 136* | Chen, M.-D., et al. (2016). Efficacy of Baduanjin exercise and feasibility of mobile text reminders on follow-up participation in people with severe mental illness: An exploratory study. Journal of Psychiatric Practice 22(3): 241-249. | No data on factors affecting user engagement |
| 138 | Choi, W. T. H., et al. (2020). Habits and attitudes of video gaming and information technology use in people with Schizophrenia: Cross-sectional survey. Journal of Medical Internet Research 22(7): e14865. | Study not about digital health tools |
| 139 | Cochran, J. M., et al. (2021). Characterization of activity behavior using a digital medicine system and comparison to medication ingestion in patients with serious mental illness. npj Digital Medicine 4(1): 63. | No data on factors affecting user engagement |
| 140* | Cohen, A. S., et al. (2020). Ambulatory digital phenotyping of blunted affect and alogia using objective facial and vocal analysis: Proof of concept. Schizophrenia Research 220: 141-146. | No data on factors affecting user engagement |
| 141 | Cole, B., et al. (2019). REPORT ON THE USE OF TELEHEALTH IN EARLY INTERVENTION IN COLORADO: STRENGTHS AND CHALLENGES WITH TELEHEALTH AS A SERVICE DELIVERY METHOD. INTERNATIONAL JOURNAL OF TELEREHABILITATION 11(1): 33-40. | Digital tool is purely video/phone calls, appointment system, EHR, or used only in person |
| 142 | Confino, J., et al. (2017). Role of social media and the Internet in pathways to care for adolescents and young adults with psychotic disorders and non-psychotic mood disorders. Early Intervention in Psychiatry 11(4): 290-295. | Study not about digital health tools |
| 143 | Connor, C., et al. (2016). Don't turn your back on the symptoms of psychosis: The results of a proof-of-principle, quasi-experimental intervention to reduce duration of untreated psychosis. BMC Psychiatry 16. | Study not about digital health tools |
| 144 | Corring, D., et al. (2012). A smart apartment for psychiatric inpatients. Psychiatric Services 63(5): 508. | Study not about digital health tools |
| 145 | Costa, D., et al. (2020). Improving treatment decision-making in bipolar II disorder: A phase II randomised controlled trial of an online patient decision-aid. BMC Psychiatry 20(1): 447. | No data on factors affecting user engagement |
| 146 | Cox, C. R., et al. (2021). Validating Biobehavioral Technologies for Use in Clinical Psychiatry. Frontiers in Psychiatry 12: 503323. | Sample <50% SMI or MH professionals |
| 147 | Crespi, S., et al. (2013). Treatment and early intervention in psychosis program (TIPP-Lausanne): Implementation of an early intervention programme for psychosis in Switzerland. Early Intervention in Psychiatry 7(3): 322-328. | Study not about digital health tools |
| 148 | Cuesta-Frau, D., et al. (2020). Classification of Actigraphy Records from Bipolar Disorder Patients Using Slope Entropy: A Feasibility Study. ENTROPY 22(11). | No data on factors affecting user engagement |
| 149 | Czaja, S. J., et al. (2017). Assessing functional performance using computer-based simulations of everyday activities. Schizophrenia Research 183: 130-136. | Not an empirical study |
| 150* | Dabit, S., et al. (2021). Improving social functioning in people with schizophrenia-spectrum disorders via mobile experimental interventions: Results from the CLIMB pilot trial. Schizophrenia Research: Cognition 26: 100211. | No data on factors affecting user engagement |
| 151 | D'Alfonso, S., et al. (2017). Artificial intelligence-assisted online social therapy for youth mental health. Frontiers in Psychology 8. | Not an empirical study |
| 152 | D'Arcey, J., et al. (2020). A randomized controlled trial of sms text messaging to improve clinical engagement in early psychosis. Neuropsychopharmacology 45: 124-125. | Not published in peer reviewed journal |
| 153 | Daumit, G. L., et al. (2019). Care Coordination and Population Health Management Strategies and Challenges in a Behavioral Health Home Model. Medical Care 57(1): 79-84. | Study not about digital health tools |
| 155 | Daus, H., et al. (2020). Development of an Emotion-Sensitive mHealth Approach for Mood-State Recognition in Bipolar Disorder. JMIR Mental Health 7(7). | Not an empirical study |
| 157* | de Almeida, R. S., et al. (2018). Mobile application for self-management in schizophrenia: A pilot study. Journal of Technology in Human Services 36(4): 179-190. | No data on factors affecting user engagement |
| 158 | de Leeuw, R. and I. Boerema (2012). Self management for people with psychotic vulnerability: a feasibility study. INTERNATIONAL JOURNAL OF INTEGRATED CARE 12. | Not published in peer reviewed journal |
| 159 | Deb, K. S., et al. (2018). Is India ready for mental health apps (MHApps)? A quantitative-qualitative exploration of caregivers' perspective on smartphone-based solutions for managing severe mental illnesses in low resource settings. PLoS ONE 13(9): e0203353. | No data on factors affecting user engagement |
| 161 | Dewa, L. H., et al. (2019). Young adults' perceptions of using wearables, social media and other technologies to detect worsening mental health: A qualitative study. PLoS ONE 14(9): e0222655. | Sample <50% SMI or MH professionals |
| 163 | Doran, D., et al. (2010). A pilot study of an electronic interprofessional evidence-based care planning tool for clients with mental health problems and addictions. Worldviews on evidence-based nursing / Sigma Theta Tau International, Honor Society of Nursing 7(3): 174-184. | Digital tool is purely video/phone calls, appointment system, EHR, or used only in person |
| 164 | Doryab, A., et al. (2015). Impact factor analysis: combining prediction with parameter ranking to reveal the impact of behavior on health outcome. PERSONAL AND UBIQUITOUS COMPUTING 19(2): 355-365. | No data on factors affecting user engagement |
| 165 | Dupuy, M., et al. (2018). Mobile cognitive testing in patients with schizophrenia: A controlled study of feasibility and validity. Journal de Therapie Comportementale et Cognitive 28(4): 204-213. | Digital tool not for monitoring/improving physical or mental health of people with SMI |
| 166* | Eack, S. M., et al. (2010). Web-based psychoeducational intervention for persons with schizophrenia and their supporters: One-year outcomes. Psychiatric Services 61(11): 1099-1105. | No data on factors affecting user engagement |
| 167 | Eiring, Ø., et al. (2017). The development and feasibility of a personal health-optimization system for people with bipolar disorder. BMC Med Inform Decis Mak 17(1): 102. | Sample <50% SMI or MH professionals |
| 170 | Elliott, A. L., et al. (2021). Changing practice: assessing attitudes toward a NICE-informed collaborative treatment pathway for bipolar disorder. BJPsych Open 7(2). | Digital tool is purely video/phone calls, appointment system, EHR, or used only in person |
| 171 | El-Mallakh, P., et al. (2014). Challenges of implementing a medication management evidence-based practice in a community mental health setting: results of a qualitative study. Issues in mental health nursing 35(7): 517-525. | Digital tool is purely video/phone calls, appointment system, EHR, or used only in person |
| 172 | Emden, D., et al. (2021). Technical feasibility and adherence of the Remote Monitoring Application in Psychiatry (ReMAP) for the assessment of affective symptoms. Journal of Affective Disorders 294: 652-660. | Sample <50% SMI or MH professionals |
| 173 | Ennis, L., et al. (2012). Can't surf, won't surf: The digital divide in mental health. Journal of Mental Health 21(4): 395-403. | Study not about digital health tools |
| 174 | Ennis, L., et al. (2014). Collaborative development of an electronic Personal Health Record for people with severe and enduring mental health problems. BMC Psychiatry 14. | Sample <50% SMI or MH professionals |
| 177 | Etingen, B., et al. (2019). How Do Patients with Mental Health Diagnoses Use Online Patient Portals? An Observational Analysis from the Veterans Health Administration. Administration and policy in mental health 46(5): 596-608. | Sample <50% SMI or MH professionals |
| 178 | Faber, K., et al. (2018). Digital trajectories to care in first-episode psychosis. Psychiatric Services 69(12): 1259-1263. | Study not about digital health tools |
| 179 | Faurholt-Jepsen, M. (2018). Electronic monitoring in bipolar disorder. Danish medical journal 65(3). | Not an empirical study |
| 180 | Faurholt-Jepsen, M., et al. (2015). Daily electronic self-monitoring in bipolar disorder using smartphones - the MONARCA I trial: a randomized, placebo-controlled, single-blind, parallel group trial. Psychological medicine 45(13): 2691-2704. | No data on factors affecting user engagement |
| 183 | Fazeli, P. L., et al. (2015). UPSA-M: Feasibility and initial validity of a mobile application of the UCSD Performance-Based Skills Assessment. Schizophrenia Research 164(1-3): 187-192. | Digital tool is purely video/phone calls, appointment system, EHR, or used only in person |
| 185 | Fernandez-Aranda, F., et al. (2012). Video games as a complementary therapy tool in mental disorders: PlayMancer, a European multicentre study. Journal of Mental Health 21(4): 364-374. | Sample <50% SMI or MH professionals |
| 186 | Ferrari, M. and A. Suzanne (2017). Youth mental health, family practice, and knowledge translation video games about psychosis: Family physicians' perspectives. Journal of the Canadian Academy of Child and Adolescent Psychiatry 26(3): 184-189. | Sample <50% SMI or MH professionals |
| 187 | Ferron, J. C., et al. (2011). Developing a quit smoking website that is usable by people with severe mental illnesses. Psychiatric rehabilitation journal 35(2): 111-116. | Sample <50% SMI or MH professionals |
| 189 | Filip, T., et al. (2019). Gamified Mobile Cognitive Tests: Preliminary Feasibility and Acceptability in Persons with Bipolar Disorder. ARCHIVES OF CLINICAL NEUROPSYCHOLOGY 34(6): 984-984. | Not published in peer reviewed journal |
| 190 | Firth, J., et al. (2018). The validity and value of self-reported physical activity and accelerometry in people with schizophrenia: A population-scale study of the UK biobank. Schizophrenia Bulletin 44(6): 1293-1300. | No data on factors affecting user engagement |
| 195 | Forma, F., et al. (2021). DIGITAL HEALTH TOOLS TO MONITOR MEDICATION ADHERENCE AMONG BIPOLAR DISORDER, MAJOR DEPRESSIVE DISORDER AND SCHIZOPHRENIA. Value in Health 24: S133-S134. | Not published in peer reviewed journal |
| 196 | Fortney, J. C., et al. (2018). Implementation of evidence-based practices for complex mood disorders in primary care safety net clinics. Families, systems & health : the journal of collaborative family healthcare 36(3): 267-280. | Study not about digital health tools |
| 197 | Fortuna, K. L., et al. (2018). Certified Peer Specialists and Older Adults With Serious Mental Illness' Perspectives of the Impact of a Peer-Delivered and Technology-Supported Self-Management Intervention. J Nerv Ment Dis 206(11): 875-881. | Sample <50% SMI or MH professionals |
| 198 | Fortuna, K. L., et al. (2018). Feasibility, Acceptability, and Preliminary Effectiveness of a Peer-Delivered and Technology Supported Self-Management Intervention for Older Adults with Serious Mental Illness. Psychiatric Quarterly 89(2): 293-305. | Sample <50% SMI or MH professionals |
| 199 | Fortuna, K. L., et al. (2018). Integration of Peer Philosophy into a Standardized Self-Management Mobile Health Intervention. Psychiatric Quarterly 89(4): 795-800. | Sample <50% SMI or MH professionals |
| 200 | Fortuna, K. L., et al. (2018). Smartphone Ownership, Use, and Willingness to Use Smartphones to Provide Peer-Delivered Services: Results from a National Online Survey. Psychiatric Quarterly 89(4): 947-956. | Sample <50% SMI or MH professionals |
| 201 | Fortuna, K. L., et al. (2019). Text message exchanges between older adults with serious mental illness and older certified peer specialists in a smartphone-supported self-management intervention. Psychiatric rehabilitation journal 42(1): 57-63. | Sample <50% SMI or MH professionals |
| 202 | Fosuaah, A., et al. (2019). A qualitative study of the acceptability of cognitive bias modification for paranoia (CBM-pa) in patients with psychosis. BMC Psychiatry 19(1): 225. | Digital tool is purely video/phone calls, appointment system, EHR, or used only in person |
| 203 | Fox, L. G. and D. Barnes (2016). Boosterbuddy: Using gamification as a compensatory strategy for motivational deficits. Psychiatric Services 67(1): 141-142. | Not an empirical study |
| 204 | Fraccaro, P., et al. (2019). Behavioural Phenotyping of Daily Activities Relevant to Social Functioning Based on Smartphone-Collected Geolocation Data. Stud Health Technol Inform 264: 945-949. | Sample <50% SMI or MH professionals |
| 207 | Garcia-Penalvo, F. J., et al. (2016). Psychiatric Patients Tracking Through a Private Social Network for Relatives: Development and Pilot Study. Journal of Medical Systems 40(7): 172. | Not an empirical study |
| 208* | Garety, P., et al. (2021). Effects of SlowMo, a Blended Digital Therapy Targeting Reasoning, on Paranoia among People with Psychosis: A Randomized Clinical Trial. JAMA Psychiatry 78(7): 714-725. | No data on factors affecting user engagement |
| 209 | Gaudiano, B. A., et al. (2018). Time-lagged predictors of daily medication nonadherence beliefs during the month post-hospital discharge in patients with psychotic-spectrum disorders. Psychiatry Res 270: 253-256. | Digital tool not for monitoring/improving physical or mental health of people with SMI |
| 210 | Gay, K., et al. (2016). Digital Technology Use Among Individuals with Schizophrenia: Results of an Online Survey. JMIR Mental Health 3(2). | Study not about digital health tools |
| 211 | Gershon, A. and P. Eidelman (2015). Inter-episode affective intensity and instability: predictors of depression and functional impairment in bipolar disorder. J Behav Ther Exp Psychiatry 46: 14-18. | Study not about digital health tools |
| 213 | Gleeson, J. F. M., et al. (2012). Moderated online social therapy for recovery from early psychosis. Psychiatric Services 63(7): 719. | Not an empirical study |
| 214 | Glick, G., et al. (2016). Use of mobile technology in a community mental health setting. Journal of telemedicine and telecare 22(7): 430-435. | Study not about digital health tools |
| 215 | Gliddon, E., et al. (2017). Discussion forum engagement within an internet-based program for bipolar disorder: impacts on social support, stigma, quality of life and mood severity. Bipolar Disorders 19: 53-53. | Not published in peer reviewed journal |
| 216 | Gliddon, E., et al. (2019). A randomized controlled trial of MoodSwings 2.0: An internet-based self-management program for bipolar disorder. Bipolar Disorders 21(1): 28-39. | No data on factors affecting user engagement |
| 217 | Glynn, S. M., et al. (2010). A proof of concept trial of an online psychoeducational program for relatives of both veterans and civilians living with schizophrenia. Psychiatric rehabilitation journal 33(4): 278-287. | No data on factors affecting user engagement |
| 218* | Golas, A. C., et al. (2015). Cognitive remediation for older community-dwelling individuals with schizophrenia: A pilot and feasibility study. International Journal of Geriatric Psychiatry 30(11): 1129-1134. | No data on factors affecting user engagement |
| 219 | Goodsmith, N., et al. (2021). Computerized conjoint analysis of the weight treatment preferences of individuals with schizophrenia. Psychiatric Services 72(3): 288-294. | Study not about digital health tools |
| 222 | Gottlieb, J. D., et al. (2013). Web-based cognitive-behavioral therapy for auditory hallucinations in persons with psychosis: A pilot study. Schizophrenia Research 145(1-3): 82-87. | Digital tool is purely video/phone calls, appointment system, EHR, or used only in person |
| 223 | Gottlieb, J. D., et al. (2017). Randomized controlled trial of an internet cognitive behavioral skills-based program for auditory hallucinations in persons with psychosis. Psychiatric rehabilitation journal 40(3): 283-292. | Digital tool is purely video/phone calls, appointment system, EHR, or used only in person |
| 224 | Gowarty, M. A., et al. (2021). Acceptability and Usability of Mobile Apps for Smoking Cessation Among Young Adults With Psychotic Disorders and Other Serious Mental Illness. Frontiers in Psychiatry 12: 656538. | Sample <50% SMI or MH professionals |
| 226 | Granholm, E., et al. (2013). Ecological Momentary Assessment of social functioning in schizophrenia: Impact of performance appraisals and affect on social interactions. Schizophrenia Research 145(1-3): 120-124. | Digital tool not for monitoring/improving physical or mental health of people with SMI |
| 227 | Grasa, E., et al. (2019). Results of the m-RESIST feasibility study. A Mobile therapeutic attention for treatment-resistant schizophrenia. INTERNATIONAL JOURNAL OF INTEGRATED CARE 19. | No data on factors affecting user engagement |
| 228 | Greenwood, K., et al. The impact of Patient and Public Involvement in the SlowMo study: Reflections on peer innovation. HEALTH EXPECTATIONS. | Study not about digital health tools |
| 229 | Grünerbl, A., et al. (2015). Smartphone-based recognition of states and state changes in bipolar disorder patients. IEEE J Biomed Health Inform 19(1): 140-148. | No data on factors affecting user engagement |
| 230 | Gumley, A., et al. (2018). Implementation of a Digital Software System for Recovery from Psychosis Early Signs. Early Intervention in Psychiatry 12: 31-31. | Not published in peer reviewed journal |
| 231 | Gyllensten, A. L. and K. A. Forsberg (2017). Computerized physical activity training for persons with severe mental illness - experiences from a communal supported housing project. Disabil Rehabil Assist Technol 12(8): 780-788. | Sample <50% SMI or MH professionals |
| 232 | Hansen, J. P., et al. (2013). The feasibility of cognitive adaptation training for outpatients with schizophrenia in integrated treatment. Community mental health journal 49(6): 630-635. | Study not about digital health tools |
| 237 | Harvey, P. D., et al. (2021). Capturing clinical symptoms with ecological momentary assessment: Convergence of momentary reports of psychotic and mood symptoms with diagnoses and standard clinical assessments. Innovations in Clinical Neuroscience 18(1-3): 24-30. | No data on factors affecting user engagement |
| 239 | Heffner, J. L., et al. (2020). Pilot randomized controlled trial of web-delivered acceptance and commitment therapy versus smokefree.gov for smokers with bipolar disorder. Nicotine and Tobacco Research 22(9): 1543-1552. | No data on factors affecting user engagement |
| 240* | Henson, P. and J. Torous (2020). Feasibility and correlations of smartphone meta-data toward dynamic understanding of depression and suicide risk in schizophrenia. International journal of methods in psychiatric research 29(2): e1825. | No data on factors affecting user engagement |
| 241* | Herse, C., et al. (2020). Brief interventions for improving adherence in schizophrenia: A pilot study using electronic medication event monitoring. Psychiatry Research 285: 112780. | No data on factors affecting user engagement |
| 242 | Hidalgo-Mazzei, D., et al. (2016). Initial usability and feasibility evaluation of the SIMPLe Smartphone application to monitor and psychoeducate bipolar patients. European Psychiatry 33: S122-S122. | Not published in peer reviewed journal |
| 246 | Holden, J. L., et al. (2020). What Do People with Schizophrenia Do All Day? Ecological Momentary Assessment of Real-World Functioning in Schizophrenia. Schizophrenia Bulletin 46(2): 242-251. | Digital tool not for monitoring/improving physical or mental health of people with SMI |
| 247 | Hollandare, F., et al. (2015). Internet-Based Cognitive Behavioral Therapy for Residual Symptoms in Bipolar Disorder Type II: A Single-Subject Design Pilot Study. JMIR RESEARCH PROTOCOLS 4(2). | No data on factors affecting user engagement |
| 248 | Hosgelen, E. I., et al. (2020). Prevelance use of technological devices and internet among patients diagnosed with schizophrenia and schizoaffective disorder. Schizophrenia Bulletin 46(Supplement 1): S173. | Not published in peer reviewed journal |
| 249 | Houle, J., et al. (2019). Getting Better My Way: Feasibility Study of a Self-Management Support Tool for People With Mood and Anxiety Disorders. Psychiatric rehabilitation journal 42(2): 158-168. | Sample <50% SMI or MH professionals |
| 252 | Jain, N., et al. (2015). Opportunities and barriers in service delivery through mobile phones (mHealth) for Severe Mental Illnesses in Rajasthan, India: A multi-site study. Asian Journal of Psychiatry 14: 31-35. | Study not about digital health tools |
| 253 | Janssen, B., et al. (2010). Improving outpatient treatment in schizophrenia: Effects of computerized guideline implementation-results of a multicenter-study within the German Research Network on Schizophrenia. European Archives of Psychiatry and Clinical Neuroscience 260(1): 51-57. | Digital tool is purely video/phone calls, appointment system, EHR, or used only in person |
| 254 | Jego, M., et al. (2019). Caring for patients with mental disorders in primary care: a qualitative study on French GPs' views, atittudes and needs. Fam Pract 36(1): 72-76. | Sample <50% SMI or MH professionals |
| 255 | Johnson, S. L., et al. (2020). A brief online intervention to address aggression in the context of emotion-related impulsivity for those treated for bipolar disorder: Feasibility, acceptability and pilot outcome data. Journal of Behavioral and Cognitive Therapy 30(1): 65-74. | No data on factors affecting user engagement |
| 256 | Jonathan, G. K., et al. (2017). Augmenting mHealth With Human Support: Notes From Community Care of People With Serious Mental Illnesses. Psychiatric rehabilitation journal 40(3): 336-338. | Not an empirical study |
| 259 | Jones, R. B., et al. (2020). A Digital Intervention for Adolescent Depression (MoodHwb): Mixed Methods Feasibility Evaluation. JMIR Mental Health 7(7). | Participants are children under 16 |
| 260 | Jones, S. H., et al. (2017). Web-based integrated bipolar parenting intervention for parents with bipolar disorder: a randomised controlled pilot trial. Journal of child psychology and psychiatry, and allied disciplines 58(9): 1033-1041. | Digital tool not for monitoring/improving physical or mental health of people with SMI |
| 261 | Joseph, H. L., et al. (2021). Group Cognitive-Behavioral Therapy via Telebehavioral Health for Those With Psychotic Spectrum Disorders: A Case Series. Cognitive and Behavioral Practice. | Digital tool is purely video/phone calls, appointment system, EHR, or used only in person |
| 263 | Kane, I., et al. (2012). Feasibility of pedometers for adults with schizophrenia: pilot study. Journal of psychiatric and mental health nursing 19(1): 8-14. | Digital tool not delivered via app, texts, website or wearable |
| 266 | Kannisto, K. A., et al. (2017). Factors Associated With Dropout During Recruitment and Follow-Up Periods of a mHealth-Based Randomized Controlled Trial for Mobile.Net to Encourage Treatment Adherence for People With Serious Mental Health Problems. Journal of Medical Internet Research 19(2): e46. | Duplicate |
| 267 | Karadzhov, D. (2021). Expanding the methodological repertoire of participatory research into homelessness: The utility of the mobile phone diary. Qualitative Social Work: Research and Practice 20(3): 813-831. | Digital tool not for monitoring/improving physical or mental health of people with SMI |
| 268 | Kaufmann, C. N., et al. (2016). Clinical significance of mobile health assessed sleep duration and variability in bipolar disorder. Journal of Psychiatric Research 81: 152-159. | Digital tool not delivered via app, texts, website or wearable |
| 269 | Kerner, B., et al. (2019). Preferences of Information Dissemination on Treatment for Bipolar Disorder: Patient-Centered Focus Group Study. JMIR Mental Health 6(6). | Study not about digital health tools |
| 270 | Khoja, S., et al. (2016). Impact of simple conventional and Telehealth solutions on improving mental health in Afghanistan. Journal of telemedicine and telecare 22(8): 495-498. | Sample <50% SMI or MH professionals |
| 272 | Killackey, E., et al. (2011). Using internet enabled mobile devices and social networking technologies to promote exercise as an intervention for young first episode psychosis patients. BMC Psychiatry 11: 80. | Not an empirical study |
| 273* | Kim, S. W., et al. (2016). Development and feasibility of smartphone application for cognitive behavioural case management of individuals with early psychosis. Early Intervention in Psychiatry 10: 188-188. | No data on factors affecting user engagement |
| 274 | Kimhy, D., et al. (2010). Concurrent measurement of real-world stress and arousal in individuals with psychosis: assessing the feasibility and validity of a novel methodology. Schizophr Bull 36(6): 1131-1139. | Digital tool not delivered via app, texts, website or wearable |
| 275 | Kimhy, D., et al. (2017). Autonomic Regulation and Auditory Hallucinations in Individuals With Schizophrenia: An Experience Sampling Study. Schizophr Bull 43(4): 754-763. | Digital tool not delivered via app, texts, website or wearable |
| 276 | King, D. B., et al. (2016). An ecological momentary sampling tool for movement patterns and psychiatric symptom variability: A pilot study. Gerontechnology 14(2): 105-109. | Sample <50% SMI or MH professionals |
| 277 | Kinney, A., et al. (2015). Peer health coaching for overweight and obese individuals with serious mental illness: intervention development and initial feasibility study. Translational Behavioral Medicine 5(3): 277-284. | Sample <50% SMI or MH professionals |
| 278 | Klee, A., et al. (2016). Interest in Technology-Based Therapies Hampered by Access: A Survey of Veterans With Serious Mental Illnesses. Psychiatric rehabilitation journal 39(2): 173-179. | Sample <50% SMI or MH professionals |
| 279 | Klein, P. J., et al. (2020). Differential medication attitudes to antihypertensive and mood stabilizing agents in response to an automated text-messaging adherence enhancement intervention. Journal of Behavioral and Cognitive Therapy 30(1): 57-64. | No data on factors affecting user engagement |
| 280 | Knights, J., et al. (2020). Detection of behavioral anomalies in medication adherence patterns among patients with serious mental illness engaged with a digital medicine system. JMIR Mental Health 7(9): e21378. | Sample <50% SMI or MH professionals |
| 281 | Koivunen, M., et al. (2010). Effects of the implementation of the web-based patient support system on staff's attitudes towards computers and IT use: A randomised controlled trial. Scandinavian Journal of Caring Sciences 24(3): 592-599. | Study not about digital health tools |
| 282 | Komatsu, H., et al. (2013). Effectiveness of Information Technology Aided Relapse Prevention Programme in Schizophrenia excluding the effect of user adherence: A randomized controlled trial. Schizophrenia Research 150(1): 240-244. | Study not about digital health tools |
| 283 | Kopelovich, S. L. and D. Turkington (2021). Remote CBT for Psychosis During the COVID-19 Pandemic: Challenges and Opportunities. Community mental health journal 57(1): 30-34. | Not an empirical study |
| 285* | Krzystanek, M., et al. (2019). A telemedicine platform to improve clinical parameters in paranoid schizophrenia patients: Results of a one-year randomized study. Schizophrenia Research 204: 389-396. | No data on factors affecting user engagement |
| 286 | Kung, N. J., et al. (2021). Usability and acceptability of two smartphone apps for smoking cessation among young adults with serious mental illness: Mixed methods study. JMIR Mental Health 8(7): e26873. | Sample <50% SMI or MH professionals |
| 287 | Kuo, C.-C., et al. (2017). A pilot comparative study of one-way versus two-way text message program to promote physical activity among people with severe mental illness. Mental Health and Physical Activity 13: 143-151. | No data on factors affecting user engagement |
| 288 | Kuosmanen, L., et al. (2010). Usability evaluation of a web-based patient information system for individuals with severe mental health problems. Journal of advanced nursing 66(12): 2701-2710. | No data on factors affecting user engagement |
| 289* | Lahti, A. C., et al. (2021). Clinical utility of wearable sensors and patient-reported surveys in patients with schizophrenia: Noninterventional, observational study. JMIR Mental Health 8(8): e26234. | No data on factors affecting user engagement |
| 290 | Laine, A., et al. (2016). Modification of an Internet-based patient education program for adults with schizophrenia spectrum disorder to suit adolescents with psychosis. Informatics for health & social care 41(3): 230-246. | Participants are children under 16 |
| 293 | Latalova, K., et al. (2014). Internet psychoeducation for bipolar affective disorder: basis for preparation and first experiences. Psychiatr Q 85(2): 241-255. | No data on factors affecting user engagement |
| 294 | Lauder, S., et al. (2013). Development of an online intervention for bipolar disorder. www.moodswings.net.au. Psychology, health & medicine 18(2): 155-165. | No data on factors affecting user engagement |
| 295 | Lauder, S., et al. (2015). A randomized head to head trial of MoodSwings.net.au: An internet based self-help program for bipolar disorder. Journal of Affective Disorders 171: 13-21. | No data on factors affecting user engagement |
| 296 | Layman, D. M., et al. (2018). Use of a web-based shared decision-making program: Impact on ongoing treatment engagement and antipsychotic adherence. Psychiatric Services 69(12): 1215-1221. | Digital tool is purely video/phone calls, appointment system, EHR, or used only in person |
| 297 | Layman, D., et al. (2019). Implementation and Use of a Client-Facing Web-Based Shared Decision-Making System (MyCHOIS-CommonGround) in Two Specialty Mental Health Clinics. Community mental health journal 55(4): 641-650. | Digital tool is purely video/phone calls, appointment system, EHR, or used only in person |
| 299 | Lee, H., et al. (2014). Telephone-delivered physical activity intervention for individuals with serious mental illness: a feasibility study. Journal of the American Psychiatric Nurses Association 20(6): 389-397. | Study not about digital health tools |
| 300 | Levin, J. B., et al. (2015). Use of automated medication adherence monitoring in bipolar disorder research: pitfalls, pragmatics, and possibilities. Therapeutic Advances in Psychopharmacology 5(2): 76-87. | Digital tool not delivered via app, texts, website or wearable |
| 301 | Levin, J. B., et al. (2019). Outcomes of Psychoeducation and a Text Messaging Adherence Intervention Among Individuals With Hypertension and Bipolar Disorder. Psychiatric services (Washington, D.C.) 70(7): 608-612. | No data on factors affecting user engagement |
| 303* | Lewis, S., et al. (2020). Smartphone-enhanced symptom management in psychosis: Open, randomized controlled trial. Journal of Medical Internet Research 22(8): e17019. | No data on factors affecting user engagement |
| 304 | Lieberman, D. Z., et al. (2010). A randomized comparison of online and paper mood charts for people with bipolar disorder. Journal of Affective Disorders 124(1-2): 85-89. | No data on factors affecting user engagement |
| 305 | Lin, E. C. L., et al. (2020). Development and feasibility testing of a continuous moodsensor smartphone application to construct an early-relapse recognition model for individuals with bipolar disorder. Bipolar Disorders 22: 118-118. | Not published in peer reviewed journal |
| 307 | Lindberg, S., et al. (2019). Unlocking design potential: Design with people diagnosed with schizophrenia. Inform Health Soc Care 44(1): 31-47. | Study not about digital health tools |
| 308 | Lobban, F. (2016). Feasibility and acceptability of web-based enhanced relapse prevention for bipolar disorder (ERPonline). Bipolar Disorders 18: 68-68. | Not published in peer reviewed journal |
| 309 | Loi, S. M., et al. (2017). A pilot study using apps as a novel strategy for the management of challenging behaviors seen in people living in residential care. International Psychogeriatrics 29(4): 637-643. | Sample <50% SMI or MH professionals |
| 310 | Londono, T., et al. Creative Technology-Based Strategies for Engaging Young People with Serious Mental Health Conditions in Longitudinal Mental Health Services Research. CHILD AND ADOLESCENT SOCIAL WORK JOURNAL. | Sample <50% SMI or MH professionals |
| 312 | Looijmans, A., et al. (2017). Design of the Lifestyle Interventions for severe mentally ill Outpatients in the Netherlands (LION) trial; a cluster randomised controlled study of a multidimensional web tool intervention to improve cardiometabolic health in patients with severe mental illness. BMC Psychiatry 17. | Not an empirical study |
| 313 | Looijmans, A., et al. (2020). Cost-effectiveness and budget impact of a lifestyle intervention to improve cardiometabolic health in patients with severe mental illness. GLOBAL & REGIONAL HEALTH TECHNOLOGY ASSESSMENT 7(1): 131-138. | Sample <50% SMI or MH professionals |
| 314 | Lopez-Morinigo, J. D., et al. (2021). Pending challenges to e-mental health in the COVID-19 era: Acceptability of a smartphone-based ecological momentary assessment application among patients with schizophrenia spectrum disorders. European Psychiatry 64: S343-S343. | Not published in peer reviewed journal |
| 318 | Madden, D. R., et al. (2020). Real-Time Data Collection to Examine Relations Between Physical Activity and Affect in Adults With Mental Illness. Journal of sport & exercise psychology: 1-8. | Sample <50% SMI or MH professionals |
| 319 | Maijala, R., et al. (2015). Internet delivered question and answer column for patients with schizophrenia. Informatics for health & social care 40(3): 267-278. | Study not about digital health tools |
| 320 | Mandiberg, J. M. and L. B. Gates (2017). A community of practice for peer mental workers: Lessons learned. American Journal of Psychiatric Rehabilitation 20(2): 156-174. | Sample <50% SMI or MH professionals |
| 321 | Martinez-Gras, I., et al. (2019). Digital Technology for Internet Access by Patients With Early-Stage Schizophrenia in Spain: Multicenter Research Study. Journal of Medical Internet Research 21(4): e11824. | Study not about digital health tools |
| 322* | Mateos, R., et al. (2016). E-Motional Training: Pilot study on a novel online training program on social cognition for patients with schizophrenia. Schizophrenia Research: Cognition 4: 10-17. | No data on factors affecting user engagement |
| 324 | Maurino, J., et al. (2010). Short message service (SMS)-based strategy to improve antipsychotic adherence among patients with schizophrenia. Value in Health 13(7): A242. | Not published in peer reviewed journal |
| 325 | Mausbach, B. T., et al. (2016). The temporal course and clinical correlates of subjective impulsivity in bipolar disorder as revealed through ecological momentary assessment. Journal of Affective Disorders 193: 145-150. | Digital tool not for monitoring/improving physical or mental health of people with SMI |
| 327 | McEnery, C., et al. (2019). Development of a Moderated Online Intervention to Treat Social Anxiety in First-Episode Psychosis. Frontiers in Psychiatry 10. | No data on factors affecting user engagement |
| 328* | McEnery, C., et al. (2021). Social anxiety in young people with first-episode psychosis: Pilot study of the EMBRACE moderated online social intervention. Early Intervention in Psychiatry 15(1): 76-86. | No data on factors affecting user engagement |
| 332 | Menon, V., et al. (2018). Therapeutic effects of mobile-based text message reminders for medication adherence in bipolar I disorder: Are they maintained after intervention cessation? Journal of Psychiatric Research 104: 163-168. | No data on factors affecting user engagement |
| 334 | Meyer, N., et al. (2016). Detecting Early Signs of Relapse in Psychosis Using Remote Monitoring Technology: Acceptability and Feasibility of a Passive Sensing Approach. Early Intervention in Psychiatry 10: 112-112. | Not published in peer reviewed journal |
| 336 | Miklowitz, D. J., et al. (2012). Facilitated Integrated Mood Management for adults with bipolar disorder. Bipolar Disorders 14(2): 185-197. | Study not about digital health tools |
| 337 | Miklowitz, D. J., et al. (2021). Development and Open Trial of a Technology-Enhanced Family Intervention for Adolescents at Risk for Mood Disorders. J Affect Disord 281: 438-446. | Participants are children under 16 |
| 339* | Minor, K. S., et al. (2021). Personalizing interventions using real-world interactions: Improving symptoms and social functioning in schizophrenia with tailored metacognitive therapy. Journal of Consulting and Clinical Psychology. | No data on factors affecting user engagement |
| 343* | Montes, J. M., et al. (2012). A short message service (SMS)-based strategy for enhancing adherence to antipsychotic medication in schizophrenia. Psychiatry Research 200(2-3): 89-95. | No data on factors affecting user engagement |
| 344 | Moore, D. J., et al. (2015). Individualized Texting for Adherence Building (iTAB): Improving Antiretroviral Dose Timing Among HIV-Infected Persons with Co-occurring Bipolar Disorder. AIDS AND BEHAVIOR 19(3): 459-471. | No data on factors affecting user engagement |
| 345 | Moran, E. K., et al. (2018). Emotion regulation predicts everyday emotion experience and social function in schizophrenia. Clinical Psychological Science 6(2): 271-279. | Digital tool not for monitoring/improving physical or mental health of people with SMI |
| 346* | Moritz, S., et al. (2015). Metacognition-augmented cognitive remediation training reduces jumping to conclusions and overconfidence but not neurocognitive deficits in psychosis. Frontiers in Psychology 6. | No data on factors affecting user engagement |
| 347* | Moritz, S., et al. (2016). Effects of online intervention for depression on mood and positive symptoms in schizophrenia. Schizophrenia Research 175(1-3): 216-222. | No data on factors affecting user engagement |
| 352 | Muralidharan, A., et al. (2018). Impact of Online Weight Management With Peer Coaching on Physical Activity Levels of Adults With Serious Mental Illness. Psychiatric services (Washington, D.C.) 69(10): 1062-1068. | Sample <50% SMI or MH professionals |
| 354 | Murray, G., et al. (2011). Self-management strategies used by 'high functioning' individuals with bipolar disorder: from research to clinical practice. Clinical psychology & psychotherapy 18(2): 95-109. | Study not about digital health tools |
| 356 | Mutschler, J., et al. (2012). Application of electronic diaries in patients with schizophrenia and bipolar disorders. Psychiatria Danubina 24(2): 206-210. | No data on factors affecting user engagement |
| 357 | Naslund, J. A., et al. (2015). Feasibility of popular m-health technologies for activity tracking among individuals with serious mental illness. Telemedicine journal and e-health : the official journal of the American Telemedicine Association 21(3): 213-216. | Sample <50% SMI or MH professionals |
| 359 | Naslund, J. A., et al. (2016). Feasibility of Behavioral Weight Loss Treatment Enhanced with Peer Support and Mobile Health Technology for Individuals with Serious Mental Illness. Psychiatric Quarterly 87(3): 401-415. | Sample <50% SMI or MH professionals |
| 360 | Naslund, J. A., et al. (2016). How people with serious mental illness use smartphones, mobile apps, and social media. Psychiatric rehabilitation journal 39(4): 364-367. | Study not about digital health tools |
| 363 | Nicholson, J., et al. (2018). The WorkingWell Mobile Phone App for Individuals With Serious Mental Illnesses: Proof-of-Concept, Mixed-Methods Feasibility Study. JMIR Mental Health 5(4). | Sample <50% SMI or MH professionals |
| 365 | Niendam, T., et al. (2015). Preliminary Longitudinal Study Examining the Clinical Correlates of Medication Adherence Assessed via a Mobile Health Application in Early Psychosis Care. Neuropsychopharmacology 40: S576-S576. | Not published in peer reviewed journal |
| 367 | Olmos-Ochoa, T. T., et al. (2019). Barriers to participation in web-based and in-person weight management interventions for serious mental illness. Psychiatr Rehabil J 42(3): 220-228. | Sample <50% SMI or MH professionals |
| 368 | Ozeki, T., et al. (2021). Impression Survey and Grounded Theory Analysis of the Development of Medication Support Robots for Patients with Schizophrenia. JOURNAL OF ROBOTICS AND MECHATRONICS 33(4): 747-755. | Digital tool not delivered via app, texts, website or wearable |
| 371 | Papola, D., et al. (2018). Can a digital medicine system improve adherence to antipsychotic treatment? Epidemiology and Psychiatric Sciences 27(3): 227-229. | Not an empirical study |
| 372 | Pelletier, J.-F., et al. (2013). No personalization without participation: on the active contribution of psychiatric patients to the development of a mobile application for mental health. BMC medical informatics and decision making 13: 78. | Sample <50% SMI or MH professionals |
| 373 | Pemovska, T., et al. (2021). Implementing a digital mental health intervention for individuals with psychosis - a multi-country qualitative study. BMC Psychiatry 21(1): 468. | Digital tool is purely video/phone calls, appointment system, EHR, or used only in person |
| 376 | Petros, R. and P. Solomon (2021). How Adults With Serious Mental Illness Learn and Use Wellness Recovery Action Plan's Recovery Framework. Qualitative health research 31(4): 631-642. | Study not about digital health tools |
| 378 | Pratt, S. I., et al. (2013). Feasibility and effectiveness of an automated telehealth intervention to improve illness self-management in people with serious psychiatric and medical disorders. Special Issue: Illness/Wellness Management for Individuals With Serious Mental Illnesses 36(4): 297-305. | Sample <50% SMI or MH professionals |
| 379 | Prociow, P. A. and J. A. Crowe (2010). Towards personalised ambient monitoring of mental health via mobile technologies. TECHNOLOGY AND HEALTH CARE 18(4-5): 275-284. | Sample <50% SMI or MH professionals |
| 383 | Rahman, T. (2019). Should Trackable Pill Technologies Be Used to Facilitate Adherence Among Patients Without Insight? AMA journal of ethics 21(4): E332-E336. | Not an empirical study |
| 385 | Rauschenberg, C., et al. (2021). A Compassion-Focused Ecological Momentary Intervention for Enhancing Resilience in Help-Seeking Youth: Uncontrolled Pilot Study. JMIR Mental Health 8(8). | Sample <50% SMI or MH professionals |
| 388 | Richters, B., et al. (2016). Blended internet care for patients with severe mental illnesses: An open label prospective controlled cohort pilot study. Internet Interventions 5: 51-55. | Sample <50% SMI or MH professionals |
| 389 | Riley, M., et al. (2016). ApTiC: A feasibility trial of a communication method using mobile technology to improve assessment within an early intervention service. European Psychiatry 33: S608-S608. | Not published in peer reviewed journal |
| 391 | Robotham, D., et al. (2015). Electronic personal health records for people with severe mental illness; a feasibility study. BMC Psychiatry 15(1): 192. | Sample <50% SMI or MH professionals |
| 392 | Robotham, D., et al. (2016). Do We Still Have a Digital Divide in Mental Health? A Five-Year Survey Follow-up. Journal of Medical Internet Research 18(11): e309. | Study not about digital health tools |
| 394 | Rodriguez-Villa, E., et al. (2021). Psychiatric rehabilitation through teaching smartphone skills to improve functional outcomes in serious mental illness. Internet Interventions 23: 100366. | Study not about digital health tools |
| 395 | Rohatagi, S., et al. (2016). Optimization of a digital medicine system in psychiatry. Journal of Clinical Psychiatry 77(9): e1101-e1107. | No data on factors affecting user engagement |
| 396 | Ross, M. K., et al. (2021). Naturalistic smartphone keyboard typing reflects processing speed and executive function. Brain and Behavior. | No data on factors affecting user engagement |
| 399 | Ruegg, N., et al. (2018). Metacognitive training online: A pilot study of an Internet-based intervention for people with schizophrenia. Zeitschrift fur Neuropsychologie 29(1): 35-47. | Not written in English |
| 400 | Rusten, K. and M. D. Thomas (2019). Using CIRCuiTS cognitive remediation therapy to improve the functioning of adults with schizophrenia. Brain Impairment 20(3): 368. | No data on factors affecting user engagement |
| 402 | Sajatovic, M., et al. (2015). A technology-enabled adherence enhancement system for people with bipolar disorder: Results from a feasibility and patient acceptance analysis. Patient Preference and Adherence 9: 753-758. | Study not about digital health tools |
| 403 | Sanghara, H., et al. (2010). Using short message services in mental health services: Assessing feasibility. Mental Health Review Journal 15(2): 28-33. | No data on factors affecting user engagement |
| 405 | Schärer, L. O., et al. (2015). Validation of life-charts documented with the personal life-chart app - a self-monitoring tool for bipolar disorder. BMC Psychiatry 15: 49. | No data on factors affecting user engagement |
| 406* | Scherer, E. A., et al. (2017). Analyzing mHealth Engagement: Joint Models for Intensively Collected User Engagement Data. JMIR mHealth and uHealth 5(1). | No data on factors affecting user engagement |
| 408 | Schlosser, D., et al. (2016). Feasibility, Acceptability, and Preliminary Outcome of PRIME: A Mobile App Intervention to Treat Young People with Schizophrenia. Early Intervention in Psychiatry 10: 75-75. | Not published in peer reviewed journal |
| 409 | Schrank, B., et al. (2010). How patients with schizophrenia use the internet: qualitative study. Journal of Medical Internet Research 12(5): e70. | Study not about digital health tools |
| 410 | Schwartz, S., et al. (2016). Daily mood monitoring of symptoms using smartphones in bipolar disorder: A pilot study assessing the feasibility of ecological momentary assessment. J Affect Disord 191: 88-93. | No data on factors affecting user engagement |
| 411 | Scott, J., et al. (2017). A pilot study to determine whether combinations of objectively measured activity parameters can be used to differentiate between mixed states, mania, and bipolar depression. International Journal of Bipolar Disorders 5(1): 5. | No data on factors affecting user engagement |
| 413 | Selvakumar, N., et al. (2017). Effect Of Twice Weekly Text Message Reminders On Medication Adherence In Bipolar Disorder (BP): A Randomized Controlled Trial. Indian Journal of Psychiatry 59(6): S151-S151. | Not published in peer reviewed journal |
| 414* | Shin, S., et al. (2016). Activity monitoring using a mHealth device and correlations with psychopathology in patients with chronic schizophrenia. Psychiatry Research 246: 712-718. | No data on factors affecting user engagement |
| 415* | Sibeko, G., et al. (2017). Improving adherence in mental health service users with severe mental illness in South Africa: a pilot randomized controlled trial of a treatment partner and text message intervention vs. treatment as usual. BMC research notes 10(1): 584. | No data on factors affecting user engagement |
| 416 | Sidana, A., et al. (2020). Telemedicine for mental health during COVID-19: Need and accessibility by patients of community outreach clinics. JOURNAL OF MENTAL HEALTH AND HUMAN BEHAVIOUR 25(2): 138-142. | Sample <50% SMI or MH professionals |
| 417 | Simmons, M. B., et al. (2018). The CHOICE pilot project: Challenges of implementing a combined peer work and shared decision-making programme in an early intervention service. Early Interv Psychiatry 12(5): 964-971. | Sample <50% SMI or MH professionals |
| 419 | Sin, J., et al. (2019). Usability evaluation of an eHealth intervention for family carers of individuals affected by psychosis: A mixed-method study. DIGITAL HEALTH 5. | Sample <50% SMI or MH professionals |
| 420 | Smith, D. J., et al. (2011). Beating Bipolar: exploratory trial of a novel Internet-based psychoeducational treatment for bipolar disorder. Bipolar Disord 13(5-6): 571-577. | No data on factors affecting user engagement |
| 421 | Snethen, G., et al. (2021). The relationship between community participation and physical activity among individuals with serious mental illnesses. Mental Health and Physical Activity 20: 100381. | Sample <50% SMI or MH professionals |
| 422 | So, S. H., et al. (2013). Detecting improvements in acute psychotic symptoms using experience sampling methodology. Psychiatry Res 210(1): 82-88. | Digital tool not delivered via app, texts, website or wearable |
| 423 | Solis, A. A., et al. (2019). Mobile therapeutic attention for treatment-resistant schizophrenia: a prospective multicenter feasibility study in patients, caregivers and clinicians. European Neuropsychopharmacology 29: S104-S105. | Not published in peer reviewed journal |
| 424* | Spaniel, F., et al. (2012). Effectiveness of the information technology-aided program of relapse prevention in schizophrenia (ITAREPS): A randomized, controlled, double-blind study. Journal of Psychiatric Practice 18(4): 269-280. | No data on factors affecting user engagement |
| 425* | Spaniel, F., et al. (2015). Psychiatrist's adherence: a new factor in relapse prevention of schizophrenia. A randomized controlled study on relapse control through telemedicine system. Journal of psychiatric and mental health nursing 22(10): 811-820. | No data on factors affecting user engagement |
| 426 | Sperry, S. H., et al. (2020). Emotion dynamics concurrently and prospectively predict mood psychopathology. J Affect Disord 261: 67-75. | Sample <50% SMI or MH professionals |
| 428 | Stanislaus, S., et al. (2021). Automatically Generated Smartphone Data in Young Patients With Newly Diagnosed Bipolar Disorder and Healthy Controls. Frontiers in Psychiatry 12: 559954. | No data on factors affecting user engagement |
| 431 | Steinwachs, D. M., et al. (2011). A web-based program to empower patients who have schizophrenia to discuss quality of care with mental health providers. Psychiatric Services 62(11): 1296-1302. | Digital tool not for monitoring/improving physical or mental health of people with SMI |
| 432 | Stentzel, U., et al. (2021). Telemedical care and quality of life in patients with schizophrenia and bipolar disorder: results of a randomized controlled trial. BMC Psychiatry 21(1): 318. | No data on factors affecting user engagement |
| 433 | Stockton, M. A., et al. (2021). Technology and implementation science to forge the future of evidence-based psychotherapies: the PRIDE scale-up study. Evidence-based mental health 24(1): 19-24. | Sample <50% SMI or MH professionals |
| 435 | Stubbe, D. E. (2020). Enhancing adherence: Using mobile health technology to improve self-management for individuals with schizophrenia. Focus (United States) 18(4): 424-427. | Not an empirical study |
| 436 | Stubbs, B., et al. (2017). Relationship Between Objectively Measured Sedentary Behavior and Cognitive Performance in Patients With Schizophrenia Vs Controls. Schizophrenia Bulletin 43(3): 566-574. | Digital tool not delivered via app, texts, website or wearable |
| 437 | Suzuki, K. and T. Watabe (2015). Internet communication of outpatients with Asperger's disorder or schizophrenia in Japan. Asia-Pacific Psychiatry 7(1): 27-35. | Study not about digital health tools |
| 439 | Swendsen, J., et al. (2011). Real-time electronic ambulatory monitoring of substance use and symptom expression in schizophrenia. Am J Psychiatry 168(2): 202-209. | Digital tool not delivered via app, texts, website or wearable |
| 441 | Tanioka, T., et al. (2021). The experience of older persons with mental health conditions who interact with healthcare robots and nurse intermediaries: The qualitative case studies. BELITUNG NURSING JOURNAL 7(4): 346-353. | Digital tool not delivered via app, texts, website or wearable |
| 442 | Terp, M., et al. (2016). A room for design: Through participatory design young adults with schizophrenia become strong collaborators. International journal of mental health nursing 25(6): 496-506. | Study not about digital health tools |
| 448 | Thurman, W., et al. (2021). Without my medication, I'm a wreck: Photo-elicitation to explore medication use among people experiencing homelessness. Research in social & administrative pharmacy : RSAP. | Sample <50% SMI or MH professionals |
| 449 | Tobitt, S. and R. Percival (2019). Switched on or switched off? A survey of mobile, computer and Internet use in a community mental health rehabilitation sample. Journal of Mental Health 28(1): 4-10. | Sample <50% SMI or MH professionals |
| 451 | Todd, N. J., et al. (2014). A web-based self-management intervention for Bipolar Disorder 'living with bipolar': a feasibility randomised controlled trial. Journal of Affective Disorders 169: 21-29. | No data on factors affecting user engagement |
| 454 | Torous, J., et al. (2016). New Tools for New Research in Psychiatry: A Scalable and Customizable Platform to Empower Data Driven Smartphone Research. JMIR Mental Health 3(2). | Not an empirical study |
| 456 | Townley, G., et al. Community Mental Health Center Visits and Community Mobility of People with Serious Mental Illnesses: A Facilitator or Constraint? Community mental health journal. | Digital tool not for monitoring/improving physical or mental health of people with SMI |
| 457 | Townsend, L., et al. (2016). Technology and opportunity: People with serious mental illness and social connection. Journal of the Society for Social Work and Research 7(2): 371-393. | Digital tool not for monitoring/improving physical or mental health of people with SMI |
| 458 | Treisman, G. J., et al. (2016). Perspectives on the use of eHealth in the management of patients with schizophrenia. Journal of Nervous and Mental Disease 204(8): 620-629. | Not an empirical study |
| 459 | Triliva, S., et al. (2020). Healthcare professionals' perspectives on mental health service provision: A pilot focus group study in six European countries. International Journal of Mental Health Systems 14(1): 16. | Study not about digital health tools |
| 460 | Tully, L. M., et al. (2014). Using a Novel Mobile Health Application to Monitor Symptoms and Functioning in an Early Psychosis Program: Preliminary Data on Feasibility and Acceptability. Biological Psychiatry 75(9): 387S-387S. | Not published in peer reviewed journal |
| 461 | Ujike, S., et al. (2019). Encounter of pepper-CPGE for the elderly and patients with schizophrenia: An innovative strategy to improve patient's recreation, rehabilitation, and communication. Journal of Medical Investigation 66(1.2): 50-53. | Study not about digital health tools |
| 462 | Vadas, L., et al. (2018). Use of information and communication technologies among individuals with and without serious mental illness. Psychiatry Research 266: 160-167. | Sample <50% SMI or MH professionals |
| 465 | Vakhrusheva, J., et al. (2014). Emotional granularity and social functioning in individuals with schizophrenia: An experience sampling study. Journal of Psychiatric Research 53(1): 141-148. | Digital tool not for monitoring/improving physical or mental health of people with SMI |
| 466 | Vakhrusheva, J., et al. (2014). Use of mobile assessment technologies in inpatient psychiatric settings. Asian Journal of Psychiatry 10: 90-95. | Digital tool not delivered via app, texts, website or wearable |
| 467 | Vakhrusheva, J., et al. (2016). Use of active-play video games to enhance aerobic fitness in schizophrenia: Feasibility, safety, and adherence. Psychiatric Services 67(2): 240-243. | Digital tool is purely video/phone calls, appointment system, EHR, or used only in person |
| 470 | Valenza, G., et al. (2014). Maximal-radius multiscale entropy of cardiovascular variability: a promising biomarker of pathological mood states in bipolar disorders. Annu Int Conf IEEE Eng Med Biol Soc 2014: 6663-6666. | Sample <50% SMI or MH professionals |
| 471 | Valenza, G., et al. (2014). Wearable monitoring for mood recognition in bipolar disorder based on history-dependent long-term heart rate variability analysis. IEEE J Biomed Health Inform 18(5): 1625-1635. | No data on factors affecting user engagement |
| 473 | van Aubel, E., et al. (2020). Blended care in the treatment of subthreshold symptoms of depression and psychosis in emerging adults: A randomised controlled trial of Acceptance and Commitment Therapy in Daily-Life (ACT-DL). Behav Res Ther 128: 103592. | Sample <50% SMI or MH professionals |
| 476* | van der Krieke, L., et al. (2013). A web-based tool to support shared decision making for people with a psychotic disorder: randomized controlled trial and process evaluation. Journal of Medical Internet Research 15(10): e216. | No data on factors affecting user engagement |
| 477 | van Dijk, F., et al. (2018). The Personal Antipsychotic Choice Index: Introducing a Tool for Shared Decision-Making in Selecting Antipsychotic Medication. Pharmacopsychiatry 51(3): 89-99. | Study not about digital health tools |
| 479 | Varnfield, M., et al. (2019). Health-e Minds: a Participatory Personalised and Gamified mHealth Platform to Support Healthy Living Behaviours for People with Mental Illness. Annual International Conference of the IEEE Engineering in Medicine and Biology Society. IEEE Engineering in Medicine and Biology Society. Annual International Conference 2019: 6943-6947. | Not an empirical study |
| 480 | Ventura, J., et al. (2013). Cognitive training at home in schizophrenia is feasible. Schizophrenia Research 143(2-3): 397-398. | No data on factors affecting user engagement |
| 481 | Vermeulen, H., et al. (2021). A blended electronic illness management and recovery program for people with severe mental illness: qualitative process evaluation alongside a randomized controlled trial. JMIR Mental Health 8(1): e20860. | Sample <50% SMI or MH professionals |
| 482 | Vilardaga, R., et al. (2018). User-Centered Design of Learn to Quit, a Smoking Cessation Smartphone App for People With Serious Mental Illness. JMIR SERIOUS GAMES 6(1). | Sample <50% SMI or MH professionals |
| 484 | Villagonzalo, K. A., et al. (2018). Predictors of engagement with a digital intervention for promoting personal recovery in persisting psychosis. European Psychiatry 48: S225-S225. | Not published in peer reviewed journal |
| 485 | Villagonzalo, K.-A., et al. (2019). Predictors of overall and mental health-related internet use in adults with psychosis. Psychiatry Research 278: 12-18. | Digital tool not for monitoring/improving physical or mental health of people with SMI |
| 486 | Watson, A., et al. (2021). The digital divide: factors impacting on uptake of remote therapy in a South London psychological therapy service for people with psychosis. Journal of Mental Health. | Digital tool is purely video/phone calls, appointment system, EHR, or used only in person |
| 487 | Weiss-Lambrou, R., et al. (2018). Use of a mobile device in mental health rehabilitation: A clinical and comprehensive analysis of 11 cases. Neuropsychological rehabilitation 28(5): 832-863. | Study not about digital health tools |
| 488 | Wenze, S. J., et al. (2014). Feasibility and Acceptability of a Mobile Intervention to Improve Treatment Adherence in Bipolar Disorder: A Pilot Study. Behavior modification 38(4): 497-515. | Digital tool not delivered via app, texts, website or wearable |
| 490 | Whiteman, K. L., et al. (2017). A peer- and technology-supported self-management intervention. Psychiatric Services 68(4): 420. | Not an empirical study |
| 494 | Wong, K. T. G., et al. (2020). Smartphone and internet access and utilization by people with Schizophrenia in South Australia: Quantitative survey study. JMIR Mental Health 7(1): e11551. | Study not about digital health tools |
| 495 | Woolridge, S., et al. (2020). Patterns and perceptions of face-to-face and digital communication in the clinical high risk and early stages of psychosis. Psychiatry Research 284: 112667. | Sample <50% SMI or MH professionals |
| 496 | Wright, K., et al. (2020). Psychological therapy for mood instability within bipolar spectrum disorder: A single-arm feasibility study of a dialectical behaviour therapy-informed approach. Pilot and Feasibility Studies 6(1): 46. | No data on factors affecting user engagement |
| 499* | Xu, W., et al. (2020). The Centroid Cannot Hold: Comparing Sequential and Global Estimates of Coherence as Indicators of Formal Thought Disorder. AMIA Annu Symp Proc 2020: 1315-1324. | No data on factors affecting user engagement |
| 500 | Ybarra, M. L., et al. (2019). Developing Texting for Relapse Prevention: A Scalable mHealth Program for People With Schizophrenia and Schizoaffective Disorder. J Nerv Ment Dis 207(10): 854-862. | Duplicate |
| 501 | Zhang, T. H., et al. (2021). Calculating individualized risk components using a mobile app-based risk calculator for clinical high risk of psychosis: findings from ShangHai At Risk for Psychosis (SHARP) program. Psychological medicine 51(4): 653-660. | Sample <50% SMI or MH professionals |
| 502* | Zhu, X., et al. (2020). A mobile health application-based strategy for enhancing adherence to antipsychotic medication in schizophrenia. Archives of psychiatric nursing 34(6): 472-480. | No data on factors affecting user engagement |
| 503 | Zulueta, J., et al. (2018). Predicting Mood Disturbance Severity with Mobile Phone Keystroke Metadata: A BiAffect Digital Phenotyping Study. Journal of Medical Internet Research 20(7): e241. | No data on factors affecting user engagement |

## Supplementary Table S2. Study design and details of digital mental health interventions.

**Note:** numbers in the first column (“#”) are study codes used to identify studies throughout this multimedia appendix. See Table S8 for full reference details and for the corresponding citation number in main manuscript reference list (where applicable).

| **#** | **Study identifier: Primary paper lead author, year, #, paper number (if >1 paper per study)** | **Name of digital tool** | **Type(s) of technology** | **Blended vs purely digital delivery** | **Focus of digital tool** | **Informed by theory** | **Stakeholder co-production** | | **Study design** | **Digital tool used within study?** | **Duration of tool use in study (weeks)** |
| --- | --- | --- | --- | --- | --- | --- | --- | --- | --- | --- | --- |
|  |  |  |  |  |  |  | **with whom** | **description** |  |  |  |
| 4 | Chiauzzi2019 | n/a | smartphone app | unclear / not described | n/a | No | n/a | NR | case study(s) | Hypothetical | n/a |
| 8 | Bonet2018 | n/a | smartphone app | unclear / not described | n/a | No | n/a | NR | cross-sectional survey | Hypothetical | n/a |
| 11 | Bucci2019a#2 | Actissist | smartphone app | no | psychosis self management | CBT | lived experience, clinicians, researchers, digital health experts | Expert reference group, beta testing | Ix development study | Hypothetical and actual use | 12 |
| 14* | Ben-Zeev2014a#1 | none given | SMS | blended | brief clinician advice and monitoring | NR | no | NR | single-arm Ix study | Actual use | 12 |
| 17 | Naslund2019 | n/a | other tech | unclear / not described | physical and mental health promotion | No | n/a | NR | cross-sectional survey | Hypothetical | n/a |
| 19 | Brunette2016 | Let's Talk About Quitting Smoking | website | set-up / tech support only | smoking cessation | Theory of Planned Behaviour | NR | NR | Ix development study | Hypothetical and actual use | 3 |
| 20 | Michalak2019#1 | none given | website | minimally blended | psychoeducation | Knowledge Transfer (KT) and community-based participatory research (CBPR) | lived experience, clinicians, researchers | co-design of website, consultation events | single-arm Ix study | Actual use | n/a |
| 22 | Vilardaga2016 | QuitPal | smartphone app | set-up / tech support only | smoking cessation | No | general population |  | single-arm Ix study | Actual use | 0.4 |
| 23 | Achytes2019#1 | FOCUS | smartphone app | blended | psychosis self management | Cognitive model of psychosis and the stress–vulnerability model | NR | NR | single-arm Ix study | Actual use | 26 |
| 24 | Ryan2020 | Wondershare | smartphone app | set-up / tech support only | symptom monitoring | No | n/a | NR | single-arm Ix study | Actual use | 3.9 |
| 26 | Bucci2018a | Actissist; ClinTouch | smartphone app | set-up / tech support only | psychosis self management / symptom monitoring | CBT | lived experience, stakeholders | Ongoing co-design | RCT | Actual use | 12 |
| 27 | Fowler2021 | Digital Medicine System (DMS) | other tech | set-up / tech support only | medication adherence | NR | NR | NR | single-arm Ix study | Actual use | 8 |
| 28 | Ferreira-Correia2018 | Cogmed | other tech | minimally blended | working memory performance | Cognitive retraining | n/a | NR | qualitative | Actual use | 5 |
| 29 | Lobban2017#1 | ERPonline | website | minimally blended | EWS monitoring, relapse prevention | early warning signs of relapse | lived experience | Input on development of Ix, feedback, user testing | RCT | Actual use | 73.7 |
| 35 | Alvarez-Jimenez2021#2 | HORYZONS | website | blended | social functioning and relapse prevention | Strengths-based and positive psychology framework | lived experience, clinicians | Iteratively developed with consultation | qualitative | Actual use | 78 |
| 40* | Henson2020#2 | Jewels Pro game (modified Trails B) within the mindLAMP mobile app | smartphone app | no | cognitive impairment prediction | experiments from previous research | not applicable | NR | observational | Actual use | 13 |
| 42* | Minor2021#2 | EAR | smartphone app | no | social functioning | Ambulatory ecological assessment research, Metacognition Reflection and Insight Therapy (MERIT) | NA | NR | secondary analysis | Actual use | 0.28 |
| 44* | Adler2020 | CrossCheck | smartphone app | no | symptom monitoring | NR | no | NR | RCT | Actual use |  |
| 45 | Ainsworth2013#1 | not yet named (eventually named CareLoop/ClinTouch) | smartphone app; SMS | set-up / tech support only | symptom monitoring | No | NR | NR | RCT | Actual use | 1.7 |
| 47 | Allan2019 | EMPOWER | smartphone app | blended | relapse prevention | early warning signs of relapse | n/a | NR | Ix development study | Hypothetical | n/a |
| 50 | Alvarez-Jimenez2021#1 | HORYZONS | website | blended | social functioning and relapse prevention | moderated online social therapy; self-determination theory | lived experience | Iteratively developed with consultation | RCT | Actual use | 78 |
| 51* | Amiri2019 | PerPal | wearable; smartphone app | no | physical activity prediction | Barkers behaviour settings theory | no | NR | observational | Actual use | 1 |
| 55 | Hanssen2020#3 | Smart website | website | no | self management | CHIME (Connectedness, Hope, Identity, Meaning, and Empowerment) conceptual framework | lived experience | developed in consultation with mental health consumers | RCT | Actual use | 12 |
| 56 | Hanssen2020#4 | Smart website | website | no | self management | CHIME (Connectedness, Hope, Identity, Meaning, and Empowerment) conceptual framework | lived experience | developed in consultation with mental health consumers | qualitative | Actual use | 12 |
| 57 | Aschbrenner2016 | n/a | other tech | blended | peer support, lifestyle intervention | No | lived experience | Pilot testing | single-arm Ix study | Actual use | 24 |
| 58* | Ben-Zeev2014a#2 | none given | SMS | no | medication monitoring and support | NR | no | NR | qualitative | Actual use | 12 |
| 62 | Austin2021 | Improving Availability and Cost-effectiveness of mental Healthcare for Schizophrenia through mHealth (IMPACHS) | smartphone app; website | minimally blended | Self monitoring and CBTp | CBT | NR | NR | single-arm Ix study | Actual use | 26 |
| 63* | Bain2017 | AiCure | smartphone app | no | medication adherence | NR | no | NR | RCT | Actual use | 24 |
| 64 | Barnes2011 | beatingbipolar.org | website | no | psychoeducation in bipolar | psychoeducation | lived experience, clinicians | Focus groups to inform Ix development | Ix development study | Hypothetical | n/a |
| 66 | Ainsworth2013#2 | not yet named (eventually named CareLoop/ ClinTouch) | smartphone app; SMS | set-up / tech support only | symptom monitoring | No | NR | NR | qualitative | Actual use | 1.7 |
| 68 | Baumel2016a | 7Cups | smartphone app; other tech | blended | emotional support | NR | clinicians | Feedback to adapt Ix | single-arm Ix study | Actual use | n/a |
| 69 | Baumel2016b | 1) FOCUS; 2) the Daily Support Web site; 3) W-CBTSp | smartphone app; website | blended | relapse prevention | CBT | NR | NR | single-arm Ix study | Actual use | 26 |
| 71 | Bechtel2021 | SPIRIT mobile system | smartphone app; other tech | blended | n/a | Unified Theory of Adoption and Use of Technology 2 (UTAUT2) used to develop the interviews | lived experience, clinicians | survey and interview feedback during trial | qualitative | Hypothetical and actual use | 26 |
| 76 | Bell2018 | RealLife™ Exp | smartphone app | blended | coping with distressing voices | coping strategy enhancement (CSE) | NR | NR | case study(s) | Actual use | 3.7 |
| 77 | Bell2020 | MovisensXS | smartphone app | blended | coping with distressing voices | coping strategy enhancement (CSE) | NR | NR | RCT | Actual use | 3.7 |
| 78 | Ben-Zeev2013 | FOCUS | smartphone app | n/a | psychosis self management | Cognitive model of psychosis and the stress–vulnerability model | lived experience, clinicians | Needs assessment survey, input on development, feedback, focus groups, user testing | Ix development study | Hypothetical | n/a |
| 80 | Ben-Zeev2014b | FOCUS | smartphone app | no | psychosis self management | CBT, stress vulnerability model | lived experience, clinicians | Iterative cycles of development and feedback | single-arm Ix study | Actual use | 4 |
| 83 | Achytes2019#2 | FOCUS | smartphone app | blended | psychosis self management | Cognitive model of psychosis and the stress–vulnerability model | NR | NR | single-arm Ix study | Actual use | 26 |
| 84 | Ben-Zeev2016 | none given | smartphone app | no | behavioural monitoring | NR | NR | NR | single-arm Ix study | Actual use | 1.5 |
| 85 | Ben-Zeev2018b#1 | FOCUS | smartphone app | set-up / tech support only | psychosis self management | Cognitive model of psychosis and the stress–vulnerability model | lived experience, clinicians | Needs assessment survey, input on development, feedback, focus groups, user testing | RCT | Actual use | 12 |
| 86 | Ben-Zeev2018a | FOCUS-AV | smartphone app | set-up / tech support only | psychosis self management | Cognitive model of psychosis and the stress–vulnerability model | NR | NR | single-arm Ix study | Actual use | 4 |
| 89 | Ben-Zeev2021 | CORE | smartphone app | minimally blended | re-appraisal of dysfunctional beliefs | NR | NR | NR | RCT | Actual use | 8.6 |
| 91 | Berry2021 | ActiGraph wGT3X-BT accelerometer, SleepBot app | smartphone app; wearable | no | monitoring physical activity levels and sleep | NR | NR | NR | observational | Actual use | 1 |
| 92 | Berry2017 | n/a | smartphone app; website | n/a | self management | n/a | n/a | NR | qualitative | Hypothetical | n/a |
| 93 | Bucci2019b | Actissist | smartphone app | no | psychosis self management | CBT, self monitoring | lived experience, clinicians | Expert reference group | qualitative | Hypothetical | n/a |
| 94 | Berry2019 | n/a | n/a | n/a | n/a | n/a | n/a | NR | qualitative | Hypothetical | n/a |
| 95 | Biagianti2016 | CLIMB | other tech | blended | social functioning | Integrated psychological therapy and cognitive enhancement therapy | NR | NR | single-arm Ix study | Actual use | 6 |
| 102 | Bonet2020 | ReMindCare | smartphone app | blended | symptom monitoring | NR | NR | NR | non-randomised Ix study | Actual use | 82.3 |
| 104 | Hidalgo-Mazzei2016#3 | SIMPLE | smartphone app | unclear / not described | symptom monitoring, psychoeducation | symptom monitoring and peronalized psychoeducation content | lived experience | user centered design approach | secondary analysis | Actual use | 13 |
| 105 | Bopp2010 | Oxford University Symptom Monitoring System (SMS) | SMS; other tech | blended | symptom monitoring | NR | NR | NR | observational | Actual use | 36 |
| 106 | Bowden2021 | KIOS | smartphone app; other tech | unclear / not described | symptom monitoring | Non-linear (chaos) theory computational software predicting trajectory of disease | lived experience, clinicians | expert panel and usability testing by patients | Ix development study | Actual use | 12 |
| 107 | Nicholas2017 | n/a | other tech | n/a | bipolar self management | n/a | n/a | NR | cross-sectional survey | Hypothetical | n/a |
| 112 | Bos2020 | RoQua (https://www.roquan/al) | smartphone app; website | minimally blended | EWS monitoring | early warning signs of relapse | lived experience, clinicians | interviews to identify relevant constructs | qualitative | Actual use | 17.3 |
| 117 | Brunette2019 | Let's Talk About Quitting Smoking | SMS; website | minimally blended | smoking cessation | Theory of Planned Behaviour | lived experience | usability testing of prototype | single-arm Ix study | Actual use | 8 |
| 120 | Bucci2018b | n/a | smartphone app | n/a | psychosis self management | CBT | n/a | NR | qualitative | Hypothetical | n/a |
| 121 | Bucci2019a#1 | Actissist | smartphone app | set-up / tech support only | psychosis self management | CBT | lived experience, clinicians, software engineers, and clinical academics | Qualitative work, beta testing, expert reference group | Ix development study | Hypothetical and actual use | 12 |
| 122 | Ben-Zeev2018b#2 | FOCUS | smartphone app | set-up / tech support only | psychosis self management | CBT | lived experience, clinicians | Needs assessment survey, input on development, feedback, focus groups, user testing | secondary analysis | Actual use | 12 |
| 126 | Camacho2021 | n/a | other tech | n/a | n/a | n/a | n/a | NR | cross-sectional survey | Hypothetical and actual use | n/a |
| 128 | Ben-Zeev2018b#3 | FOCUS | smartphone app | set-up / tech support only | psychosis self management | Cognitive model of psychosis and the stress–vulnerability model | lived experience, clinicians | Needs assessment survey, input on development, feedback, focus groups, user testing | qualitative | Actual use | 12 |
| 130 | Depp2021 | METER | SMS; website | minimally blended | facial emotion recognition | NR | NR | NR | observational | Actual use | 1.4 |
| 131* | Cella2018 | none given | wearable | blended | autonomic arousal monitoring | NR | NR | NR | observational | Actual use | 0.85 |
| 132* | Cella2019 | none given | wearable; smartphone app | no | symptom monitoring | NR | NR | NR | observational | Actual use | 1.4 |
| 135 | Kreyenbuhl2019 | MedActive | smartphone app | set-up / tech support only | medication adherence | Information-Motivation-Behavioral (IMB) Skills Model | lived experience, clinicians | Iterative process of user centered design with feedback at every stage | single-arm Ix study | Actual use | 2 |
| 136* | Chen2016 | none given | SMS | blended | Baduanjin exercise adherence | Baduanjin exercise | NR | NR | single-arm Ix study | Actual use | 8 |
| 137 | Cho2020 | CRM | smartphone app; wearable | no | relapse prevention based on passive monitoring of circadian rhythm | Theories of circadian rhythm and psychiatric symptoms | NR | NR | single-arm Ix study | Actual use | 52 |
| 140* | Cohen2020 | none given | smartphone app | no | passive monitoring of negative symptoms | Facial and vocal analysis to understand psychological states | NR | NR | observational | Actual use | NR |
| 150* | Dabit2021 | CLIMB | smartphone app | blended | social functioning | Integrated psychological therapy and cognitive enhancement therapy | NR | NR | RCT | Actual use | 9 |
| 154 | Daus2018 | n/a | smartphone app; wearable | n/a | symptom monitoring | n/a | n/a | NR | qualitative | Hypothetical | n/a |
| 156 | deAlmeida2019 | weCOPE | smartphone app | blended | symptom monitoring, self management | CBT | lived experience, clinicians | designed with clinicians, modules chosen by people with lived experience and clinicians | Usability testing | Hypothetical and actual | 8 |
| 157* | deAlmeida2018 | weCOPE | smartphone app | no | symptom monitoring, self management | CBT | lived experience, clinicians | designed with clinicians, modules chosen by people with lived experience and clinicians | single-arm Ix study | Actual use | 8 |
| 160 | Depp2015#1 | Personalized Real-Time Intervention for Stabilizing Mood (PRISM). | smartphone app | blended | self management | NR | NR | NR | RCT | Actual use | 10 |
| 162 | Lobban2017#2 | ERPonline | website | set-up / tech support only | EWS monitoring, relapse prevention | early warning signs of relapse | NR | NR | qualitative | Actual use | 52 |
| 166* | Rotondi2010 | schizophrenia online access to resources (SOAR) | website | blended | psychoeducation | family psychoeducation | NR | NR | RCT | Actual use | NR |
| 168 | Eisner2019#2 | ExPRESS | smartphone app | minimally blended | EWS monitoring | early warning signs of relapse | lived experience | Beta testing | single-arm Ix study | Actual use | 26 |
| 169 | Eisner2019#1 | ExPRESS | smartphone app | minimally blended | EWS monitoring | early warning signs of relapse | NR | NR | single-arm Ix study | Actual use | 26 |
| 175 | Enrique2020 | Bipolar toolkit | website | no | bipolar self management | personal recovery approach | lived experience, clinicians | interviews with stakeholders to explore the needs the Ix could meet | single-arm Ix study | Actual use | 10 |
| 176 | Lopez-Morinigo2021 | eB2 | smartphone app | no | physical activity, sleep and social activity monitoring | NR | NR | NR | observational | Actual use | 14 |
| 181 | Faurholt-Jepsen2019 | Triloges-Monseso app + Nokia health accelerometer; Pulso app + FitBit accelerometer | smartphone app; wearable | unclear / not described | self monitoring of symptoms, sleep and activity | NR | NR | NR | RCT | Actual use | 4 |
| 182 | Faurholt-Jepsen2020 | Monsenso system | smartphone app | unclear / not described | medication adherence self monitoring | NR | NR | NR | secondary analysis | Actual use | 7.5 |
| 184 | Fellendorf2021 | Up! | smartphone app; wearable | unclear / not described | sleep monitoring | NR | NR | NR | measure validation | Actual use | 26 |
| 188 | Ferron2012 | none given | website | blended | smoking cessation | NR | NR | NR | Usability testing | Actual use | n/a |
| 191 | Fletcher2021 | n/a | smartphone app | no | bipolar self management | n/a | n/a | NR | cross-sectional survey | Hypothetical | n/a |
| 192 | Thomas2016#1 | SMART | website | blended | symptom monitoring, self management | CHIME (Connectedness, Hope, Identity, Meaning, and Empowerment) conceptual framework | lived experience | Website content was co-designed | single-arm Ix study | Actual use | 13 |
| 193 | Forchuk2015#1 | Lawson SMART Record | website | minimally blended | self management | n/a | lived experience, clinicians, researchers | helped design the record system and training | qualitative | Actual use | 34.7 |
| 194 | Forchuk2015#2 | Lawson SMART Record | website | minimally blended | self management | n/a | lived experience, clinicians, researchers | helped design the record system and training | secondary analysis | Actual use | 78 |
| 205 | Moura2019 | COGWEB | website | blended | cognitive remediation (cognitive performance) | Cognitive remediation | NR | NR | single-arm Ix study | Actual use | 26 |
| 206 | Fulford2020 | Motivation and Skills Support (MASS) app | smartphone app | minimally blended | social skills and social motivation | Social Skills Training (SST) and cognitive behavioral therapy for psychosis (CBTp) | lived experience, clinicians | expert panel views on social needs and technology | Usability testing | Actual use | 2 |
| 208* | Garety2021 | SlowMo | smartphone app | blended | reducing paranoia | CBT | NR | NR | RCT | Actual use | NR |
| 212 | Whiteman2017 | I-IMR adapted for smartphone | smartphone app | n/a | self management | stress vulnerability model | NR | NR | usability testing | Usability testing in session | n/a |
| 218* | Golas2015 | none given | other tech | blended | cognitive remediation (cognitive performance) | cognitive remediation (cognitive performance) | NR | NR | single-arm Ix study | Actual use | 8 |
| 220 | Gorczynski2014 | ActiGraph GT1M | wearable | set-up / tech support only | physical activity monitoring | NR | NR | NR | non-randomised Ix study | Actual use | 8.7 |
| 221 | Gordon-Smith2019 | True Colours | website | no | symptom monitoring | mood monitoring | NR | NR | single-arm Ix study | Actual use | 82.5 |
| 225 | Granholm2012 | MATS | SMS | set-up / tech support only | medication adherence, socialisation, symptom monitoring | CBT | lived experience, clinicians, service managers | focus group feedback | single-arm Ix study | Actual use | 12 |
| 233 | Hanssen2020#1 | SMARTapp | smartphone app | set-up / tech support only | symptom monitoring, self management | NR | NR | NR | RCT | Actual use | 3 |
| 234 | Rotondi2021 | n/a | website | no | n/a | n/a | n/a | NR | usability testing | Usability testing in session | n/a |
| 235 | Hardy2018 | SlowMo | smartphone app; website | blended | reducing paranoia | CBT | lived experience | developed in consultation with mental health consumers | case study(s) | Actual use | 8 |
| 236 | Harris2017 | CogRem | website | no | cognitive remediation (cognitive performance) | cognitive remediation, employment support | NR | NR | RCT | Actual use | 17.3 |
| 238 | Hatch2018 | n/a | n/a | n/a | n/a | n/a | n/a | NR | cross-sectional survey | Hypothetical | n/a |
| 240* | Henson2020#1 | mindLAMP | smartphone app | no | symptom monitoring, self management | theories of survey latency relationship with symptoms | NR | NR | observational | Actual use | 12 |
| 241* | Tessier2020 | Medication Event Monitoring System (MEMS®) | smartphone app | no | medication adherence | NR | NR | NR | RCT | Actual use | 24 |
| 243 | Hidalgo-Mazzei2016#1 | SIMPLe 1.0 | smartphone app | minimally blended | symptom monitoring, psychoeducation | Evidence-based group psychoeducation program for BD (Colom et al., 2009, Colom et al., 2003). | lived experience | user centered design approach | single-arm Ix study | Actual use | 13 |
| 244 | Hidalgo-Mazzei2016#2 | SIMPLe 1.0 | smartphone app | minimally blended | symptom monitoring, psychoeducation | Evidence-based group psychoeducation program for BD (Colom et al., 2009, Colom et al., 2003). | lived experience | user centered design approach | single-arm Ix study | Actual use | 13 |
| 245 | Hidalgo-Mazzei2018 | SIMPLe 1.5 | smartphone app | no | symptom monitoring, psychoeducation | Evidence-based group psychoeducation program for BD (Colom et al., 2009, Colom et al., 2003). | lived experience | user centered design approach | single-arm Ix study | Actual use | 26 |
| 250 | Huerta-Ramos2016#1 | M-RESIST | smartphone app; wearable | blended | n/a | integrative model; empowerment, personalization of treatment; prediction of treatment outcomes | n/a | NR | qualitative | Hypothetical | n/a |
| 251 | Huerta-Ramos2016#2 | M-RESIST | smartphone app; wearable | blended | n/a | integrative model; empowerment, personalization of treatment; prediction of treatment outcomes | NR | NR | qualitative | Hypothetical | n/a |
| 257 | Jonathan2021 | LiveWell | smartphone app; website | blended | EWS monitoring | Empirically supported psychotherapies for bipolar disorder, health psychology behavior change theories, and chronic disease self-management models | lived experience | field testing, design and usability testing, pilot study with user feedback | single-arm Ix study | Actual use | 8 |
| 258 | Ben-Zeev2018b#4 | FOCUS | smartphone app | set-up / tech support only | psychosis self management | Cognitive model of psychosis and the stress–vulnerability model | lived experience, clinicians | Needs assessment survey, input on development, feedback, focus groups, user testing | qualitative | Actual use | 12 |
| 262 | Depp2015#2 | Project 1. Personalized Real-Time Intervention for Stabilizing Mood (PRISM); Project 2. Mobile Assessment and Therapy for Schizophrenia (MATS); Project 3. n/a | SMS; other tech | minimally blended | symptom monitoring, self management | Project 1. psychoeducation Project 2. cognitive and behavioral therapy interventions Project 3. n/a | stakeholders (identify NR) | stakeholders involved in development of all aspects of the protocols and interface | secondary analysis | Actual use | 7 |
| 264 | Kane2013 | Digital Health Feedback System (DHFS) | other tech | blended | medication adherence | NR | NR | NR | single-arm Ix study | Actual use | 4 |
| 265 | Välimäki2017#2 | Mobilen/aet | SMS | no | medication adherence, appointment attendance | NR | NR | NR | RCT | Actual use | 52 |
| 271 | Kidd2019 | App4Independence | smartphone app | no | psychosis self management | NR | lived experience | Prompted by discussion with person with lived experience, feedback on Ix, beta testing | single-arm Ix study | Actual use | 3.5 |
| 273* | Kim2016 | Heal Your Mind [HYM] | smartphone app | blended | CBTp / EIP | CBT | clinicians | app developed with clinicians | Ix development study | Actual use | 48 |
| 284 | Kopelowicz2017 | Digital Medicine System | other tech | blended | medication adherence | No | NR | NR | single-arm Ix study | Actual use | 8 |
| 285* | Krzystanek2019 | MONEO | smartphone app | blended | medication adherence | cognitive training, evidence around telemedicine | NR | NR | RCT | Actual use | 48 |
| 289* | Lahti2021 | Garmin Vivofit Fitness Band, Philips Actiwatch Spectrum, Smartphone | wearable; smartphone app | no | physical activity monitoring | predictive modelling with ESM and passive data | NR | NR | observational | Actual use | 16 |
| 291 | Laine2019 | MentalNet | website | blended | psychoeducation | NR | NR | NR | non-randomised Ix study | Actual use | 8 |
| 292 | Lal2015 | n/a | other tech | n/a | n/a | no | n/a | NR | cross-sectional survey | Hypothetical | n/a |
| 298 | Lederman2014 | none given | website | blended | online social therapy | Supportive Accountability and Positive Psychology | lived experience, clinicians, usability experts | focus groups involving patients, clinicians, and usability experts | Ix development study | Hypothetical and actual | 6 |
| 302 | Lewandowski2021 | CR Programme | other tech | blended | cognitive remediation (cognitive performance) | Cognitive remediation theory, NEAR model and Cognitive Enhancement Therapy | NR | NR | single-arm Ix study | Actual use | 12 |
| 303* | Lewis2020 | ClinTouch | smartphone app | no | EWS monitoring | EWS | clinicians, service users and carers | focus groups and qualitative interviews informed design, plus advisory group to trial | RCT | Actual use | 12 |
| 306 | Xu2019#2 | LEAN | SMS | minimally blended | medication adherence | Health Belief Model | lived experience, clinicians, family members, policy-makers |  | RCT | Actual use | 52 |
| 311 | Lim2020 | +Connect | smartphone app | set-up / tech support only | loneliness reduction | Positive psychology interventions (strengths-based approach aiming to enhance functioning) | lived experience, healthy controls | Focus groups to inform Ix development | single-arm Ix study | Actual use | 6 |
| 315 | Ludwig2021 | HORYZONS (USA) | website | blended | social functioning and relapse prevention | CBT, positive psychology, mindfulness, psychoeducation | lived experience | Consultation informed design | single-arm Ix study | Actual use | 12 |
| 316 | Rotondi2012 | n/a | website | no | n/a | n/a | n/a | NR | usability testing | Usability testing in session | n/a |
| 317 | Luther2020 | Mobile Enhancement of Motivation in Schizophrenia (MEMS) | SMS | blended | motivational deficits | Theory of two impaired reward processing mechanisms underlying motivation deficits in schizophrenia: effort-cost computations, and maintenance of reward-value representations | NR | NR | RCT | Actual use | 8 |
| 322* | Vázquez-Campo2016 | e-Motional Training® (ET®) | website | blended | social cognition training | social cognition | NR | NR | single-arm Ix study | Actual use | NR |
| 323 | Matthews2017 | n/a | other tech | n/a | symptom monitoring | n/a | n/a | NR | qualitative | Hypothetical | 4 |
| 326 | McClelland2018 | n/a | smartphone app | unclear / not described | symptom monitoring | NR | lived experience, clinicians | early consultation to 'appraise the concept' and feedback on a prototype | Ix development study | Hypothetical | n/a |
| 328* | McEnery2021 | EMBRACE | website | blended | social anxiety | CBT for social anxiety | young people with experience of SAD, psychosis and SMI | focus groups informed user-centered design | single-arm Ix study | Actual use | 8 |
| 329 | McKnight2017 | True Colours | SMS; other tech | minimally blended | symptom monitoring | NR | NR | NR | single-arm Ix study | Actual use | 119.2 |
| 330 | Medalia2021 | Brain HQ, Lumosity, and Scientific Brain Training Pro | website | blended |  | Cognitive remediation theory - evidence-based behavioral training intervention improves neurocognitive processes and real-world functioning | NR | NR | RCT | Actual use | 15 |
| 331 | Medenblik2020 | Mobile Contingency Management (CM) / iCOMMIT | smartphone app | blended | smoking cessation | NR for the app. Other (non-digital) elements of the programme were behavioural/cognitive-behavioural | lived experience, clinicians | qualitative information used to inform design | RCT | Actual use | 6 |
| 333 | Merzenich2014 | SocialVille | website | minimally blended | social cognition | Neuroplasticity based learning i.e. that targeting brain systems underlying impaired social cognition will improve social behaviour | NR | NR | single-arm Ix study | Actual use | 8 |
| 335 | Meyer2018 | Sleepsight (app) | smartphone app; wearable | minimally blended | physical activity and sleep monitoring | That passive sensing of rest-activity rhythms support identification of early signs of deterioration in schizophrenia | lived experience | involved throughout the development and testing cycle. Consultation groups | single-arm Ix study | Actual use | 8 |
| 338 | Miller2015 | n/a | SMS; other tech | blended | n/a | No | n/a | NR | cross-sectional survey | Hypothetical | n/a |
| 339* | Minor2021#1 | Electronically Activated Recorder (EAR) | smartphone app | blended | social functioning | Ambulatory ecological assessment research, Metacognition Reflection and Insight Therapy (MERIT) | NR | NR | RCT | Actual use | 7 |
| 340 | Mistler2017 | Headspace | smartphone app | set-up / tech support only | anger and aggression | Mindfulness | NR | NR | single-arm Ix study | Actual use | 1 |
| 341 | Moitra2017 | MyExperience Tool | other tech | set-up / tech support only | treatment adherence | No | NR | NR | observational | Actual use | 4 |
| 342 | Moitra2021 | MACS | smartphone app | no | psychosis self management | CBT | NR | NR | single-arm Ix study | Actual use | 4 |
| 343* | Montes2012 | none given | SMS | no | medication adherence | previous evidence around SMS reminders | NR | NR | RCT | Actual use | 12 |
| 346* | Moritz2015 | Mybraintraining | website | blended | cognitive functioning | meta-cognition, cognitive remediation | NR | NR | single-arm Ix study | Actual use | 0.14 |
| 347* | Moritz2016 | HelpID | website | no | psychoeducation | CBT | NR | NR | RCT | Actual use | 12 |
| 348 | Michalak2019#2 | the QoL tool | website | no | improving quality of life | NR | lived experience, clinicians, researchers | Community-Based Participatory Research process, qualitative interviews | qualitative | Actual use | NR |
| 349 | Morton2021b | n/a | smartphone app | n/a | n/a | n/a | n/a | NR | cross-sectional survey | Hypothetical | n/a |
| 350 | Morton2021a | n/a | smartphone app | n/a | n/a | n/a | n/a | NR | cross-sectional survey | Hypothetical | n/a |
| 351 | Mueller2018 | WellWave | smartphone app | no | activities of daily living | NR | NR | NR | single-arm Ix study | Actual use | 12.8 |
| 353 | Murnane2016 | n/a | n/a | n/a | symptom monitoring | n/a | n/a | NR | cross-sectional survey | Hypothetical | n/a |
| 355 | Murray2015 | ORBIT | website | no | emotion regulation | Acceptance and Commitment Therapy, mindfulness | lived experience, clinicians, researchers | developed in collaboration | single-arm Ix study | Actual use | 3 |
| 358 | Naslund2016#2 | none given | other tech; other tech | blended | physical health promotion | NR | NR | NR | single-arm Ix study | Actual use | 26 |
| 361 | Naslund2016#1 | Fitbit | wearable; other tech | blended | physical health promotion | NR | NR | NR | single-arm Ix study | Actual use | 26 |
| 362 | Proudfoot2012#2 | Bipolar Education Program (BEP) | website | blended | psychoeducation | NR | lived experience | Co-delivery | RCT | Actual use | 8 |
| 364 | Niendam2018 | Ginger.io | smartphone app | minimally blended | symptom monitoring | NR | NR | NR | observational | Actual use | 60.7 |
| 366 | Aref-Adib2016 | n/a | smartphone app; website | n/a | n/a | n/a | n/a | NR | qualitative | Hypothetical | n/a |
| 369 | Röhricht2021 | Florence | SMS | minimally blended | treatment adherence | NR | lived experience, clinicians | Focus groups at various points | RCT | Actual use | 26 |
| 370 | Palmier-Claus2012 | ClinTouch | smartphone app | no | symptom monitoring | NR | NR | NR | single-arm Ix study | Actual use | 1 |
| 374 | Peters-Strickland2016 | Otsuka | other tech | set-up / tech support only | medication adherence | NR | NR | NR | Usability testing | Actual use | 8 |
| 375 | Ybarra2019 | Texting for Relapse Prevention (T4RP) | SMS | blended | EWS monitoring | NR | lived experience, clinicians |  | qualitative | Hypothetical | n/a |
| 377 | Poole2012 | Beating Bipolar | website; other tech | blended | psychoeducation in bipolar | NR | lived experience, clinicians, family members | Focus groups to inform Ix development | RCT | Actual use | 17.3 |
| 380 | Prociow2012 | none given | wearable; other tech | n/a | relapse prediction | NR | lived experience, healthy controls | testing prototype | observational | Actual use | 1 |
| 381 | Proudfoot2012#1 | Bipolar Education Program (BEP) | website | blended | psychoeducation | NR | lived experience | Co-delivery | RCT | Actual use | 8 |
| 382 | Puskar2011 | computerized virtual health counselor named “Laura” | other tech | blended | medication adherence and psychoeducation | Relational agent's script was based on the three phases of the nurse-patient relationship identified by Peplau (1952): orientation, working, and termination. | NR | NR | case study(s) | Actual use | 4.3 |
| 384 | Raugh2021 | mEMA application from Ilumivu (ilumivu.com) and the Alert app from Empatica | smartphone app; wearable | set-up / tech support only | symptom monitoring | NR | NR | NR | single-arm Ix study | Actual use | 0.9 |
| 386 | Raymaker2020 | n/a | website | unclear / not described | self management | self-determination theory: centers competence, autonomy, and relatedness as the basic psychological needs of self-determination | lived experience, clinicians, researchers | Co-production, service users on the design team | Ix development study | Hypothetical and usability testing in session | n/a |
| 387 | Realpe2020 | SCIT intervention on Second Life Platform | website | no | social cognition | Social cognition and interaction training (SCIT) | lived experience | co-design, iterative usability screening, beta testing | Ix development study | Hypothetical | n/a |
| 390 | Roberts2017 | Mary/Eddie/Bill-iPad (MEBi) | other tech | minimally blended | social cognition | Social Cognition and Interaction Training (SCIT) | NR | NR | non-randomised Ix study | Actual use | 4 |
| 393 | Rodriguez-Villa2021 | mindLamp | smartphone app | no | relapse prevention | n/a | lived experience, clinicians, family members | iterative feedback, focus groups and interviews about usability during co-design | Ix development study | Hypothetical | n/a |
| 397 | Hatch2017 | n/a | other tech | n/a | medication adherence | n/a | n/a | NR | Ix development study | Hypothetical | n/a |
| 398 | Rotondi2015 | n/a | website | no | n/a | n/a | n/a | NR | usability testing | Usability testing in session | n/a |
| 401 | Sablier2012 | Mobus | other tech | blended | activities of daily living | NR | NR | NR | single-arm Ix study | Actual use | 6 |
| 404 | Saunders2017 | True Colours + Mood Zoom + GeneActiv/Fitbit | smartphone app; wearable | no | symptom monitoring | NR | NR | NR | qualitative | Actual use | 12 |
| 406* | Achytes2019#3 | FOCUS | smartphone app | set-up / tech support only | psychosis self management | Cognitive model of psychosis and the stress–vulnerability model | NR | NR | secondary analysis | Actual use | <24 |
| 407 | Schlosser2016 | PRIME | smartphone app | blended | motivational deficits | self-determination theory | lived experience, clinicians, family members, researchers | User-centered design process, design workshops, 1:1 interviews | single-arm Ix study | Hypothetical and actual | 12 |
| 412 | Sedgwick2021 | GRASP app | smartphone app | blended | social cognition | That social cognition improves relationships and functioning, that app use between sessions will improve generalisation of learning to daily life | lived experience | Feedback on prototype | single-arm Ix study | Actual use | 4 |
| 414* | Shin2016 | Fitbit | wearable | no | physical activity monitoring | research regarding physical activity and psychiatric symptoms | NR | NR | observational | Actual use | 1 |
| 415* | Sibeko2017 | none given | SMS | blended | treatment adherence | adherence research on health literacy, problem solving, social support and telephone prompts | NR | NR | RCT | Actual use | 36 |
| 418 | Simon2011 | MyRecoveryPlan | website | blended | self management | NR | lived experience | People with lived experience contributed toward design | RCT | Actual use | 3 |
| 424* | Spaniel2012 | ITAREPS (original) | SMS | no | EWS monitoring | prodrome based targeted medication strategy | NR | NR | RCT | Actual use | 48 |
| 425* | Spaniel2015 | none given | SMS; other tech | minimally blended | relapse prevention | prodrome based targeted medication strategy | NR | NR | RCT | Actual use | 78 |
| 427 | Sreejith2019 | n/a | n/a | n/a | n/a | n/a | n/a | NR | cross-sectional survey | Hypothetical | n/a |
| 429 | Steare2020#1 | My Journey 3 | smartphone app | minimally blended | self management | NR | lived experience, clinicians, digital health experts | Designed in collaboration, feedback on prototype | RCT | Actual use | 52 |
| 430 | Steare2020#2 | My Journey 3 | smartphone app | blended | self management | NR | lived experience, clinicians, researchers, digital health experts |  | qualitative | Actual use | 52 |
| 434 | Storm2021 | PeerTECH | smartphone app; other tech | blended | self management, peer support | Integrated Illness Management and Recovery (I-IMR) | lived experience, clinicians | usability testing | usability testing | Hypothetical and usability testing in session | n/a |
| 438 | Swartz2021 | Rhythms and You (RAY) | website | blended | developing regular sleep, activity and social rhythms | Interpersonal and social rhythm therapy (IPSRT) | NR | NR | RCT | Actual use | 12 |
| 440 | Switsers2018 | n/a | smartphone app | n/a | n/a | n/a | lived experience | focus groups | qualitative | Hypothetical | n/a |
| 443 | Terp2018 | MindFrame | smartphone app | minimally blended | self management | NR | lived experience, clinicians | interviews informed topics, then co-designed | qualitative | Actual use | 26 |
| 444 | Lal2018 | n/a | smartphone app; website | n/a | n/a | n/a | n/a | NR | qualitative | Hypothetical | n/a |
| 445 | Thomas2017 | n/a | SMS; other tech | n/a | n/a | n/a | n/a | NR | cross-sectional survey | Hypothetical | n/a |
| 446 | Wilson2019 | mCM and Stay Quit Coach smartphone apps | smartphone app | blended | smoking cessation | CBT, contingency management (CM) | NR | NR | Ix development study | Actual use | 4 |
| 447 | Thonon2022 | MetricWire app; MiBand 3 activity band | smartphone app; wearable | blended | motivational deficits | Models of motivation and goal directed behaviours | NR | NR | single-arm Ix study | Actual use | 8.7 |
| 450 | Todd2013 | n/a | website | n/a | self-management | recovery informed and broadly based on the principles of Cognitive Behavioural Therapy and psycho-education | lived experience | systematically involved service users in intervention development | qualitative | Hypothetical | n/a |
| 452 | Tolley2015 | n/a | other tech | n/a | symptom monitoring | NR | NR | NR | usability testing | Usability testing in session | n/a |
| 453 | Torous2017 | none given | other tech | blended | symptom monitoring | No | lived experience | Person with lived experience originated idea, tested it, and co-authored paper | case study(s) | Actual use | 17.3 |
| 455 | Torous2018 | n/a | smartphone app; wearable | n/a | n/a | NR | NR | NR | cross-sectional survey | Hypothetical | n/a |
| 463 | Vaessen2019 | Acceptance and Commitment Therapy (ACT) in Daily Life (ACT-DL) | smartphone app; other tech | blended | adaptive stress responses | Acceptance and Commitment Therapy | NR | NR | RCT | Actual use | NR |
| 464 | Vaidyam2020 | none given | other tech | no | environmental pollution monitoring | no | lived experience | This was a service user's own idea, in discussion with their psychiatrist | case study(s) | Actual use | 1 |
| 468 | Valentine2020 | Horyzons | website | blended | social functioning and relapse prevention | NR | NR | NR | qualitative | Hypothetical | n/a |
| 469 | Alvarez-Jimenez2021#3 | HORYZONS | website | blended | social functioning and relapse prevention | Positive psychology framework focused on self-compassion, personal strengths, and increasing self-efficacy | lived experience, clinicians | Iteratively developed with consultation | qualitative | Actual use | 78 |
| 472 | Välimäki2017#1 | Mobilen/aet | SMS | set-up / tech support only | medication adherence, appointment attendance | self-determination theory | lived experience, clinicians | Co-design of text message content | RCT | Actual use | 52 |
| 474 | vandenHeuvel2018 | PHR-BD | website | blended | self management, clinician support | NR | lived experience, clinicians, informal caregivers | Developed in collaboration | single-arm Ix study | Actual use | 52 |
| 475 | vanderKrieke2012 | WEGWEIS, which is a Dutch abbreviation that stands for web environment for empowerment and individual advice | website | minimally blended | self management | evidence-based research (eg, the Dutch Multidisciplinary Guideline for Schizophrenia), clinical expertise, and service user experiences | lived experience, clinicians | Prototype based on interviews with service users, relatives, and clinicians, then developed by researchers in collaboration with a groups of service users | usability testing | Usability testing in session | n/a |
| 476* | vanderKrieke2013 | none given | website | no | shared decision making | evidence on shared decision making and electronic decision aids | NR | NR | RCT | Actual use | <6 |
| 478 | VanTil2020 | Fitbit Alta HR + unnamed smartphone app | smartphone app; wearable | minimally blended | self monitoring of symptoms, sleep and activity | NR | NR | NR | RCT | Actual use | 6 |
| 483 | Vilardaga2019 | Learn to Quit (LTQ); NCI QuitGuide | smartphone app | blended | smoking cessation | Acceptance and Commitment Therapy | lived experience |  | case study(s) | Actual use | 4.3 |
| 489 | Wenze2016 | My Experience | smartphone app | blended | treatment adherence, symptom monitoring | Psychoeducational and cognitive-behavioral principles, plus Health Beliefs Model | NR | NR | single-arm Ix study | Actual use | 12 |
| 491 | Williams2018 | n/a | n/a | n/a | n/a | n/a | n/a | NR | qualitative | Hypothetical | n/a |
| 492 | Thomas2016#2 | SMART | website | blended | symptom monitoring, self management | CHIME (Connectedness, Hope, Identity, Meaning, and Empowerment) conceptual framework | lived experience | Website content was co-designed | qualitative | Actual use | 19.5 |
| 493 | Hanssen2020#2 | SMART | website | blended | self management | CHIME (Connectedness, Hope, Identity, Meaning, and Empowerment) conceptual framework | lived experience | developed in consultation with mental health consumers | qualitative | Actual use | 19.5 |
| 497 | Brunette2020 | Let's Talk about Smoking; Computerized National Cancer Institute Patient Education | website | set-up / tech support only | smoking cessation | NR | lived experience | Website content and interface co-designed | RCT | Usability testing in session | n/a |
| 498 | Xu2019#1 | LEAN | SMS | minimally blended | medication adherence | Health Belief Model | lived experience, clinicians, family members, policy-makers |  | RCT | Actual use | 26 |
| 499* | Xu2020 | none given | smartphone app | no | measuring thought disorder | Latent Semantic Analysis | NR | NR | observational | Actual use | NR |
| 502* | Zhu2020 | WeChat | smartphone app | no | medication adherence | NR | NR | NR | RCT | Actual use | 24 |

*Excluded from main review; included only in supplementary review of safety reporting in studies testing actual DHI use (Supplementary Results SR1; Supplementary Table S5). See Table S1 for full paper reference.

## Supplementary Table S3. Sample characteristics of reviewed studies

**Note:** numbers in the first column (“#”) are study codes used to identify studies throughout this multimedia appendix. See Table S8 for full reference details and for the corresponding citation number in main manuscript reference list (where applicable).

| **#** | **Study identifier: Primary paper lead author, year, #, paper number (if >1 paper per study)** | **SU**  **sample size** | **HCP sample size** | **Service / recruitment source** | **SSD %** | **Bi-polar %** | **Other diag-nosis %** | **Age (SU sample): mean (SD), overall range, or % in age brackets** | **Race and/or Ethnicity (SU sample)** | **Gender**  **(% male)** | **Relevant data on barriers and facilitators** | **User engagement indicators***** | |
| --- | --- | --- | --- | --- | --- | --- | --- | --- | --- | --- | --- | --- | --- |
|  |  |  |  |  |  |  |  |  |  |  |  | **Subj-ective** | **Obj-ective** |
| 4 | Chiauzzi2019 | 1 | 0 | NR | 0 | 100 | 0 | 43 (0) | NR | 0 | qualitative | Yes | No |
| 8 | Bonet2018 | 80 | 0 | EIS; CMHT | 78 | 0 | 22 | 38.1 (13) | NR | 72.4 | quantitative | Yes | No |
| 11 | Bucci2019a#2 | 54 | 65 | Outpatient | NR | NR | NR | NR | NR | NR | qualitative | Yes | No |
| 14* | Ben-Zeev2014a#1 | 17 | 0 | CMHT | 100 | 0 | 0 | 40.47 (11.56) | African-American 53%; White 35%; Asian 6%; more than one race 6% | 59 | n/a (none) | NR | NR |
| 17 | Naslund2019 | 135 | 0 | Social media | 27 | 25 | 48 | 18-35 years: 46%; 36-65 years: 53% | White 83%; Asian 3.7%; Black/African American 5.9%; Caucasian/Non-Hispanic White 83.0%; Hispanic Or Latino 3.0%; Native 2.2% | 31.9 | quantitative | Yes | No |
| 19 | Brunette2016 | 11 | 0 | Outpatient | 100 | 0 | 0 | 49 (12) | White 36%; African American 46% | 45 | qualitative | Yes | No |
| 20 | Michalak2019#1 | 43 | 0 | Support/advocacy/peer groups/charities | 0 | 100 | 0 | modal age range 45-54years | NR | 30 | qualitative | Yes | No |
| 22 | Vilardaga2016 | 5 | 0 | Outpatient | 60 | 20 | 20 | 51.2 (4.27) | NR | 100 | qualitative | Yes | No |
| 23 | Achytes2019#1 | 347 | 0 | Community mental health services | 100 | 0 | 0 | <35 years: 57%; 35–45 years: 23%; >45 years: 20% | White 49%; African American 25%; Other/Multiple 14%; Latino 11% | 63 | quantitative | No | Yes |
| 24 | Ryan2020 | 26 | 0 | Recruitment from other study sample(s) | 0 | 100 | 0 | 46.46 (10.55) | NR | 27 | quantitative | No | Yes |
| 26 | Bucci2018a | 36 | 0 | EIS | 100 | 0 | 0 | NR | White 86%; Black 11%; Asian 3% | 50 | both | Yes | Yes |
| 27 | Fowler2021 | 44 | 0 | Community mental health services; Inpatient | 100 | 0 | 0 | 34.4 (10.7) | White 79.5%; Black 18.2%; Other 2.3% | 65.9 | quantitative | No | Yes |
| 28 | Ferreira-Correia2018 | 9 | 0 | Inpatients; Support/advocacy/peer groups/charities; | 56 | 0 | 45 | 33.4 (8.41) | NR | 44.5 | qualitative | Yes | No |
| 29 | Lobban2017#1 | 96 | 0 | Mental health services; Support/advocacy/peer groups/charities; Online; Advertisements | 0 | 100 | 0 | 42.9 (11.8) | White 93%; Carribean 1%; Asian British 1%; Indian 1%; Other 1%; Missing 2% | 38.5 | both | Yes | No |
| 35 | Alvarez-Jimenez2021#2 | 12 | 0 | EIS | NR | NR | NR | 23.2 (2.6) | NR | 41.6 | qualitative | Yes | No |
| 40* | Henson2020#2 | 45 | 0 | Community mental health services | 100 | 0 | 0 | NR | NR | NR | n/a (none) | NR | NR |
| 42* | Minor2021#2 | 36 | 0 | Community mental health services | 100 | 0 | 0 | 46.06 (10.6) | Black/African American 63.9%; Multi-racial 5.6%; Non hispanic 86.1% | 44.4 | n/a (none) | NR | NR |
| 44* | Adler2020 | 60 | 0 | Mental health services; Inpatient; Advertisements | 100 | 0 | 0 | 23-50 | not available | 58.3 | n/a (none) | NR | NR |
| 45 | Ainsworth2013#1 | 24 | 0 | Community mental health services; EIS; Supported living | 100 | 0 | 0 | 33 (9.5) | NR | 79 | quantitative | Yes | Yes |
| 47 | Allan2019 | 88 | 21 | Community mental health services | 100 | 0 | 0 | NR | NR | NR | qualitative | Yes | No |
| 50 | Alvarez-Jimenez2021#1 | 170 | 0 | EIS | 100 | 0 | 0 | 20.91 (2.88) | NR | 52.9 | quantitative | No | Yes |
| 51* | Amiri2019 | 10 | 0 | NR | 100 | 0 | 0 | 54.8 (5.3) | NR | 20 | n/a (none) | NR | NR |
| 55 | Hanssen2020#3 | 98 | 0 | Community mental health services; Social media | 59 | 30 | 11 | 42 (11.1) | NR | 45 | quantitative | No | Yes |
| 56 | Hanssen2020#4 | 17 | 0 | Community mental health services; Social media | 58 | 24 | 18 | 18-34 years: 41%; 35-50 years: 35%; 51-65 years: 24% | NR | 35 | qualitative | Yes | No |
| 57 | Aschbrenner2016 | 25 | 0 | CMHT | 24 | 36 | 40 | 48.6 (11.4) | White 100% | 44 | qualitative | Yes | No |
| 58* | Ben-Zeev2014a#2 | 17 | 0 | Psychiatric rehabilitation | 100 | 0 | 0 | 40.47 (11.56) | NR | NR | n/a (none) | NR | NR |
| 62 | Austin2021 | 16 | 0 | EIS | 100 | 0 | 0 | 24.06 (NR) | NR | 25 | both | Yes | Yes |
| 63* | Bain2017 | 75 | 0 | NR | 100 | 0 | 0 | 45.9 (10.86) | Asian 5%; Black 52%; Hawaiian 1%; White 41% | 55 | n/a (none) | NR | NR |
| 64 | Barnes2011 | 11 | 10 | CMHT; Outpatient; Inpatient; Support/advocacy/peer groups/charities | 0 | 72.7 | 27.3 | NR | NR | NR | qualitative | Yes | Yes |
| 66 | Ainsworth2013#2 | 24 | 0 | Community mental health services; EIS; Supported living | 100 | 0 | 0 | 33 (9.5) | NR | 79 | qualitative | Yes | No |
| 68 | Baumel2016a | 10 | 6 | Outpatient | 100 | 0 | 0 | 28.25 (8.31) | White 20%; African-American 60%; Hispanic 20% | 60 | qualitative | Yes | No |
| 69 | Baumel2016b | 200 | 0 | Inpatient; Recent inpatients | 100 | 0 | 0 | 34.6 (10.6) | White 48%; Black Or African American 31%; Asian 4%; Hispanic Or Latino 11%; American/Alaska Native 1%; Mixed race 6% | 59 | quantitative | No | Yes |
| 71 | Bechtel2021 | 0 | 23 | Outpatient | n. | n. | n. | n/a | NR | 13 | qualitative | Yes | No |
| 76 | Bell2018 | 1 | 0 | Outpatient | 100 | 0 | 0 | 38 (0) | NR | 100 | quantitative | Yes | No |
| 77 | Bell2020 | 34 | 0 | Outpatient; Mental health services; Support/advocacy/peer groups/charities; | 82.4 | 14.8 | 3 | 40.8 (10.6) | Australian 82%; New Zealander 3%; British Or Irish 6%; Greek 6%; Other 3% | 44 | quantitative | Yes | Yes |
| 78 | Ben-Zeev2013 | 0 | 8 | Community psychiatric rehabilitiation | n/a | n/a | n/a | n/a | NR | NR | both | Yes | No |
| 80 | Ben-Zeev2014b | 33 | 0 | Community mental health services | 100 | 0 | 0 | 45.9 (8.78) | RACE: White 21%; African American 76%; Mixed race 3%. ETHNICITY: Latino 6% | 61 | quantitative | Yes | Yes |
| 83 | Achytes2019#2 | 342 | 0 | Community mental health services | 100 | 0 | 0 | 18-29 years: 40.3%; 30-45 years: 39.5%; 46-60 years 20.2% | White 50%; African-American 25.1%; Hispanic 10.8%; Asian, American Indian, Native Hawaiian, or Mixed race 14.0% | 62.3 | quantitative | No | Yes |
| 84 | Ben-Zeev2016 | 20 | 0 | Outpatient; Inpatient | 100 | 0 | 0 | 38.41 (11.5) | White 55%; Black 15%; Mixed race 20%; Declined To Respond 10% | 80 | quantitative | Yes | Yes |
| 85 | Ben-Zeev2018b#1 | 163 | 0 | Mental health services | 49 | 28 | 23 | 49 (9.9) | White 27%; African American 65%; Other/Mixed race 7% | 59 | quantitative | No | Yes |
| 86 | Ben-Zeev2018a | 10 | 0 | NR | 100 | 0 | 0 | 45.5 (13.18) | NR | 60 | both | Yes | Yes |
| 89 | Ben-Zeev2021 | 315 | 0 | Online; Advertisements; | 22 | 35 | 43 | 37.9 (11.64) | White 77%; African American 10%; Asian 3%; Native American 0.6%; Other 9% | 12 | quantitative | No | Yes |
| 91 | Berry2021 | 30 | 0 | CMHT; Inpatient; Psychiatric rehabilitation | 100 | 0 | 0 | 40.4 (10.2) | White British 63.3%; Black British/African 13.3%; Black British/Caribbean 6.7%; Other Ethnic Groups 16.7% | 83.3 | qualitative | Yes | No |
| 92 | Berry2017 | 0 | 20 | Outpatient | n/a | n/a | n/a | n/a | White 95%; British Pakistani 5% | 20 | qualitative | Yes | No |
| 93 | Bucci2019b | 0 | 48 | EIS | n/a | n/a | n/a | n/a | White 85%; Mixed 8%; Missing 6% | 41.66 | qualitative | Yes | No |
| 94 | Berry2019 | 18 | 0 | Outpatient | 44 | 56 | 0 | 37.3 (11.5) | White 83.3%; Asian British 11.5%; Black British 6.6% | 39 | qualitative | Yes | No |
| 95 | Biagianti2016 | 27 | 0 | Online; Advertisements; Support/advocacy/peer groups/charities; | 88.8 | 11.1 | 0 | 28.1 (6.4) | NR | 63 | quantitative | Yes | Yes |
| 102 | Bonet2020 | 90 | 0 | EIS | 100 | 0 | 0 | 32.8 (9.4) | NR | 73 | quantitative | Yes | Yes |
| 104 | Hidalgo-Mazzei2016#3 | 51 | 0 | Outpatient | 0 | 100 | 0 | 40.37 (10.3) | NR | 63 | quantitative | No | Yes |
| 105 | Bopp2010 | 62 | 0 | Outpatient | 0 | 100 | 0 | 34.3 (10.66) | NR | 44 | quantitative | No | Yes |
| 106 | Bowden2021 | 20 | 0 | Outpatient | 0 | 100 | 0 | NR | NR | NR | both | Yes | No |
| 107 | Nicholas2017 | 89 | 0 | Online advertisements | 0 | 100 | 0 | 24.4 (3.9) | NR | 13 | both | Yes | No |
| 112 | Bos2020 | 20 | 6 | Mental health services | 0 | 100 | 0 | 35-30 years: 45%; 36-50 years: 40%, 51-65 years: 15% | NR | 20 | qualitative | Yes | No |
| 117 | Brunette2019 | 20 | 0 | Community mental health services | 60 | 15 | 25 | 46 (13.6) | White 85%; Other 15% | 70 | both | Yes | No |
| 120 | Bucci2018b | 21 | 0 | EIS | 100 | 0 | 0 | 26 (5.14) | NR | 48 | qualitative | Yes | No |
| 121 | Bucci2019a#1 | 54 | 65 | EIS | 100 | 0 | 0 | NR | NR | NR | qualitative | Yes | No |
| 122 | Ben-Zeev2018b#2 | 163 | 0 | Mental health services | 49 | 28 | 23 | 49 (9.9) | White 27%; African American 65%; Other/Mixed race 7% | 59 | quantitative | No | Yes |
| 126 | Camacho2021 | 0 | 42 | EIS | n. | n. | n. | n/a | NR | NR | quantitative | Yes | Yes |
| 128 | Ben-Zeev2018b#3 | 31 | 0 | Mental health services | 58 | 23 | 19 | NR | White 39%; Black 48%; Mixed race 3% | 65 | qualitative | Yes | No |
| 130 | Depp2021 | 86 | 0 | Community mental health services | 80.2 | 17.5 | 2.3 | 44.1 (11.4) | RACE: White 27%; Black/African American 49%; Asian 5%; Other 20%. ETHNICITY: Hispanic 23% | 45.3 | quantitative | No | Yes |
| 131* | Cella2018 | 30 | 0 | CMHT | 100 | 0 | 0 | 18 (10.8) | NR | 33.9 | n/a (none) | NR | NR |
| 132* | Cella2019 | 15 | 0 | Mental health services | 100 | 0 | 0 | 28.1 (3.8) | NR | 80 | n/a (none) | NR | NR |
| 135 | Kreyenbuhl2019 | 7 | 7 | Outpatient | 100 | 0 | 0 | 47.6 (10.4) | African American 100% | 100 | quantitative | Yes | No |
| 136* | Chen2016 | 11 | 0 | Psychiatric rehabilitation | 81.8 | 0 | 18.2 | 38.9 (9.6) | NR | 72.7 | n/a (none) | NR | NR |
| 137 | Cho2020 | 43 | 0 | Inpatient | 0 | 72 | 28 | 25.8 (6.3) | NR | 40 | quantitative | No | Yes |
| 140* | Cohen2020 | 25 | 0 | Outpatient; Advertisements | 100 | 0 | 0 | NR | NR | NR | n/a (none) | NR | NR |
| 150* | Dabit2021 | 30 | 0 | Online | 100 | 0 | 0 | 37.3 (10.7) | NR | 45 | n/a (none) | NR | NR |
| 154 | Daus2018 | 15 | 0 | Outpatient; Support/advocacy/peer groups/charities | 0 | 100 | 0 | 44.2 (13) | NR | 60 | qualitative | Yes | No |
| 156 | deAlmeida2019 | 111 | 0 | Psychiatric rehabilitation | NR | NR | NR | 41.7 (NR) | NR | 63 | both | Yes | No |
| 157* | deAlmeida2018 | 9 | 0 | Psychiatric rehabilitation | 100 | 0 | 0 | 38.11 (9.701) | NR | 77.8 | n/a (none) | NR | NR |
| 160 | Depp2015#1 | 104 | 0 | Outpatient; Support/advocacy/peer groups/charities; Advertisements; Online | 0 | 100 | 0 | 47.5 (12.8) | White 70%; African American 9%; Asian 2%; Latino/Hispanic 15%; Mixed race 5% | 42 | quantitative | No | Yes |
| 162 | Lobban2017#2 | 19 | 0 | Recruitment from other study sample(s) | 0 | 100 | 0 | 42 (NR) | NR | 47 | qualitative | Yes | No |
| 166* | Rotondi2010 | 31 | 0 | Community mental health services; Inpatient | 100 | 0 | 0 | 38 (10.8) | White 48%; African-American 44%, Other 7% | 32.68 | n/a (none) | NR | NR |
| 168 | Eisner2019#2 | 23 | 0 | Mental health services | 100 | 0 | 0 | 37.9 (9.9) | White British 83%; Asian Or Asian British 6%; Black Or Black British 11% | 67 | qualitative | Yes | No |
| 169 | Eisner2019#1 | 18 | 0 | Mental health services | 100 | 0 | 0 | 37.9 (9.9) | White British 83%; Asian Or Asian British 6%; Black Or Black British 11% | 67 | both | Yes | Yes |
| 175 | Enrique2020 | 15 | 3 | Outpatient | 0 | 73 | 27 | 40.23 (12.02) | NR | 23 | both | Yes | Yes |
| 176 | Lopez-Morinigo2021 | 77 | 0 | Recruitment from other study sample(s); Outpatients | 100 | 0 | 0 | 47.69 (9.76) | NR | 53 | quantitative | Yes | Yes |
| 181 | Faurholt-Jepsen2019 | 59 | 0 | Outpatient; Community mental health services | 0 | 100 | 0 | 18-67 | NR | 32 | quantitative | Yes | No |
| 182 | Faurholt-Jepsen2020 | 117 | 0 | Outpatient | 0 | 100 | 0 | 30.9 (9.9) | NR | 38 | quantitative | No | Yes |
| 184 | Fellendorf2021 | 22 | 0 | Outpatient; Inpatient | 0 | 100 | 0 | 43.36 (10.89) | NR | 54.5 | quantitative | Yes | No |
| 188 | Ferron2012 | 131 | 0 | Psychiatric rehabilitation; Advertisements | 70 | 0 | 30 | 46 (10) | White 36.3%; Black 47.41; Latino 14%; Other 16% | 71.85 | quantitative | No | Yes |
| 191 | Fletcher2021 | 55 | 0 | Social media; Support/advocacy/peer groups/charities | 0 | 100 | 0 | 40.1 (12) | NR | 25.5 | quantitative | Yes | No |
| 192 | Thomas2016#1 | 10 | 0 | Community mental health services; Psychiatric rehabilitation | 100 | 0 | 0 | 42.6 (12.47) | NR | 90 | qualitative | Yes | No |
| 193 | Forchuk2015#1 | 95 | 0 | Community mental health services | NR | NR | NR | NR | NR | 59 | qualitative | Yes | No |
| 194 | Forchuk2015#2 | 394 | 52 | Community mental health services | 59.4 | 0 | 40.6 | 37.6 (13.8) | NR | 60.7 | both | Yes | No |
| 205 | Moura2019 | 17 | 0 | Outpatient | 100 | 0 | 0 | 23.6 (3.9) | NR | 80 | both | Yes | Yes |
| 206 | Fulford2020 | 8 | 0 | Community mental health services | 100 | 0 | 0 | NR | NR | NR | qualitative | Yes | No |
| 208* | Garety2021 | 361 | 0 | Community mental health services | 100 | 0 | 0 | 42.6 (11.6) | White 69%; Black Caribbean 5%; Black African 6.1%; Black other 7.8%; Indian 0.8%; Pakistani 2.2%; Chinese 0.3%; Other 8.6%; missing 0.3% | 69.8 | n/a (none) | NR | NR |
| 212 | Whiteman2017 | 10 | 0 | Mental health services | 60 | 30 | 10 | 55.3 (49-66) | White 90%; Black/African American 10% | 50 | both | Yes | No |
| 218* | Golas2015 | 18 | 0 | Older adults outpatient | 100 | 0 | 0 | 69.8 (5.3) | White 62%; Other 48% | 44.4 | n/a (none) | NR | NR |
| 220 | Gorczynski2014 | 4 | 0 | Mental health services | 100 | 0 | 0 | 29.5 (4.7) | NR | 50 | both | Yes | Yes |
| 221 | Gordon-Smith2019 | 975 | 0 | Mental health services; Advertisements; Support/advocacy/peer groups/charities; | 1.3 | 99 | 10 | 55.5 (NR) | White 90%; Black/African American 10% | 50 | quantitative | No | Yes |
| 225 | Granholm2012 | 55 | 0 | Outpatient; Inpatient | 100 | 0 | 0 | 48.7 (9.1) | White 74%; African American 7%; Hispanic 10% | 69 | quantitative | Yes | Yes |
| 233 | Hanssen2020#1 | 50 | 0 | Assertive outreach teams; Inpatient; Support/advocacy/peer groups/charities; | 100 | 0 | 0 | 33.6 (11.5) | NR | 66 | quantitative | Yes | Yes |
| 234 | Rotondi2021 | 83 | 0 | Community mental health services; Outpatient; Veterans services | 100 | 0 | 0 | 53.1 (8.7) | White 58%; African American 42% | 84 | quantitative | No | Yes |
| 235 | Hardy2018 | 14 | 0 | Community mental health services | 100 | 0 | 0 | 43.83 (11.4) | White British 58%; Black British 17%; Black African 8%; Afro-Caribbean 8%; Black Caribbean & White 8% | 42 | both | Yes | No |
| 236 | Harris2017 | 86 | 0 | Supported employment services | 68.6 | 31 | 0 | 39.55 (11.14) | NR | 64 | quantitative | No | Yes |
| 238 | Hatch2018 | 0 | 40 | Experts in the field identified from their activity | 0 | 0 | 0 | n/a | NR | NR | quantitative | No | Yes |
| 240* | Henson2020#1 | 39 | 0 | Community mental health services | 100 | 0 | 0 | 37 (14.86) | American Indian or Alaskan native 10.5%; Asian 2.6%; Black or African-American 28.9%; Multi-racial or other 2.6%; White 55.3% | 55.6 | n/a (none) | NR | NR |
| 241* | Tessier2020 | 33 | 0 | Outpatient | 100 | 0 | 0 | 38.00 (11.4) | NR | NR | n/a (none) | NR | NR |
| 243 | Hidalgo-Mazzei2016#1 | 49 | 0 | Outpatient | 0 | 100 | 0 | 43.92 (11.36) | NR | 57.1 | quantitative | Yes | Yes |
| 244 | Hidalgo-Mazzei2016#2 | 50 | 0 | Outpatient | 0 | 100 | 0 | 43.92 (11.36) | NR | 57.1 | quantitative | No | Yes |
| 245 | Hidalgo-Mazzei2018 | 201 | 0 | Outpatient; Online; Advertisements | 0 | 100 | 0 | 36.59 (11) | White 38.5%; Latin-American 60.5%; African 0.5%; Asian 0.5% | 36.8 | quantitative | Yes | Yes |
| 250 | Huerta-Ramos2016#1 | 52 | 27 | Community mental health services | 100 | 0 | 0 | n/a | NR | 42 | qualitative | Yes | No |
| 251 | Huerta-Ramos2016#2 | 14 | 13 | Outpaient | 100 | 0 | 0 | 32.92 (NR) | NR | 61.5 | qualitative | Yes | No |
| 257 | Jonathan2021 | 12 | 0 | Outpatient | 0 | 100 | 0 | 38 (14) | White 100% | 33 | both | Yes | No |
| 258 | Ben-Zeev2018b#4 | 30 | 0 | Mental health services | 72.4 | 6.9 | 20.7 | 48 (12) | White 47%; Black 40%; American Indian 3%; Mixed race 10% | 60 | qualitative | Yes | No |
| 262 | Depp2015#2 | 21 | 0 | Outpatient | 0 | 100 | 0 | 41 (13.7) | NR | NR | qualitative | Yes | No |
| 264 | Kane2013 | 28 | 0 | Mental health services; Advertisements | 57.1 | 42.9 | 0 | 42.8 (12.7) | White 42.9%; Black 25.0%; Hispanic 14.3%; Asian 3.6%; Black And Native American 3.6%; White And Hispanic 3.6%; Other7.1% | 64 | quantitative | No | Yes |
| 265 | Välimäki2017#2 | 1123 | 0 | Inpatient | 37.1 | 28.9 | 34 | 38.3 (12.5) | NR | 49.17 | quantitative | Yes | Yes |
| 271 | Kidd2019 | 38 | 0 | EIS; Support/advocacy/peer groups/charities; Snowball sampling; Recruitment from other study sample(s) | 97.4 | 2.6 | 0 | 31.42 (8.6) | White 42.1%; Black Of African Or Caribbean Origin 23.7%; Mixed 23.7%; Other 10.5% | 71.1 | both | Yes | Yes |
| 273* | Kim2016 | 24 | 0 | EIS | 100 | 0 | 0 | 25.6 (4.1) | NR | 45.83 | n/a (none) | NR | NR |
| 284 | Kopelowicz2017 | 49 | 0 | Outpatient | 31 | 45 | 24 | 46.4 (13) | White 57%; Black 37%; Asian 4%; Other 2% | 36.7 | quantitative | No | Yes |
| 285* | Krzystanek2019 | 290 | 0 | NR | 100 | 0 | 0 | 32.07 (6.3) | NR | 60.37 | n/a (none) | NR | NR |
| 289* | Lahti2021 | 40 | 0 | Outpatients | 100 | 0 | 0 | 19-68, median 40.3 | Black or African-American 73%; White 28% | 63 | n/a (none) | NR | NR |
| 291 | Laine2019 | 90 | 0 | Inpatient | 100 | 0 | 0 | 41.6 (13.5) | NR | 58 | quantitative | Yes | Yes |
| 292 | Lal2015 | 67 | 0 | EIS | 100 | 0 | 0 | 25.6 (5.1) | White 64%; Asian 14.9%; Black 7.5%; Latin American 7.5%; Arab 1.5%; Mixed 4.5% | 76.1 | quantitative | Yes | No |
| 298 | Lederman2014 | 26 | 8 | EIS | NR | NR | NR | NR | NR | NR | qualitative | Yes | No |
| 302 | Lewandowski2021 | 19 | 0 | EIS | 100 | 0 | 0 | NR | NR | NR | quantitative | Yes | Yes |
| 303* | Lewis2020 | 80 | 0 | Mental health services | 100 | 0 | 0 | 34.5 (20-68) | Black or Black British 39.5%; Other 7.4%; White 53.1% | 66.21 | n/a (none) | NR | NR |
| 306 | Xu2019#2 | 277 | 0 | Community mental health services | 100 | 0 | 0 | 46 (12.7) | NR | 51.8 | quantitative | Yes | Yes |
| 311 | Lim2020 | 12 | 0 | EIS | 100 | 0 | 0 | 20.5 (2.55) | White 66.7%; African Australian 8.3%; Asian Austraian Or Asian 25% | 75 | qualitative | Yes | No |
| 315 | Ludwig2021 | 26 | 0 | EIS | 100 | 0 | 0 | 25.1 (3.5) | RACE: White 61.5%; African American 34.6%; Asian 3.8%. ETHNICITY: Hispanic/Latino 7.7%; Non-Hispanic/Latino 92.3% | 73.1 | quantitative | Yes | Yes |
| 316 | Rotondi2012 | 149 | 0 | Community mental health services; Outpatient | 50.3 | 14.8 | 35 | 52.9 (8.4) | White 48%; African American 48%; Asian 3% | 78.5 | quantitative | No | Yes |
| 317 | Luther2020 | 56 | 0 | Community mental health services | 100 | 0 | 0 | 46.2 (8.8) | White 25%; African American 69.7%; Other Or Mixed race 5.4% | 51.8 | quantitative | Yes | No |
| 322* | Vázquez-Campo2016 | 19 | 0 | Community mental health services | 100 | 0 | 0 | 39.90 (9.2) | NR | 63.15 | n/a (none) | NR | NR |
| 323 | Matthews2017 | 10 | 0 | University based bipolar prevention program | 0 | 100 | 0 | 25-34 years: 40%, 35-44 years: 20%, 45-54 years: 20%, 55-64 years: 20% | NR | 50 | qualitative | Yes | No |
| 326 | McClelland2018 | 6 | 8 | EIS | 100 | 0 | 0 | NR | NR | NR | qualitative | Yes | No |
| 328* | McEnery2021 | 10 | 0 | NR | NR | NR | NR | 23 (3.2) | NR | 40 | n/a (none) | NR | NR |
| 329 | McKnight2017 | 297 | 0 | Outpatient; Inpatients; Advertisements | 0 | 100 | 0 | 41 (13.7) | White 93.5%; Mixed Black 1.55%; Mixed Asian 0.31%; Other 5.64% | 33.3 | quantitative | No | Yes |
| 330 | Medalia2021 | 56 | 6 | Outpatient | 100 | 0 | 0 | 45.71 (12.73) | RACE: White 33%; American Indian Or Alaska Native 1.8%; Asian 3.6%; Black Or African American 58.2%; Mixed race: 1.8%; Unknown 1.8%. ETHNICITY: Hispanic/Latinx 30.9%; Not Hispanic/Latinx 69.1% | 70.9 | both | Yes | Yes |
| 331 | Medenblik2020 | 35 | 0 | Community mental health services | 100 | 0 | 0 | 48.2 (9.9) | White 12%; Black/African American 62%; Mixed race 24%; American Indian/Alaska Native 2% | 80 | quantitative | Yes | No |
| 333 | Merzenich2014 | 17 | 0 | EIS; Outpatient | 100 | 0 | 0 | 23.8 (3.2) | NR | 76 | quantitative | Yes | Yes |
| 335 | Meyer2018 | 15 | 0 | Outpatient | 100 | 0 | 0 | 44.1 (30-54) | NR | 60 | both | Yes | Yes |
| 338 | Miller2015 | 80 | 0 | Inpatient, outpatient | 100 | 0 | 0 | 41 (13) | White 31%; African 56%; Other 11% | 51 | quantitative | Yes | No |
| 339* | Minor2021#1 | 20 | 0 | Community mental health services; Online | 100 | 0 | 0 | 44.37 (10.9) | African american 75%; Caucasian 25% | 45.39 | n/a (none) | NR | NR |
| 340 | Mistler2017 | 12 | 0 | Inpatient | 75 | 25 | 0 | 33.4 (10.7) | NR | 83 | qualitative | Yes | No |
| 341 | Moitra2017 | 65 | 0 | Inpatient; Partial hospital settings | 100 | 0 | 0 | 37.2 (13.4) | White 79%; African American 5%; Latino 9%; Other Ethno-Racial Origins 4% | 45 | both | Yes | Yes |
| 342 | Moitra2021 | 10 | 0 | Inpatient | 100 | 0 | 0 | 44.4 (13.9) | RACE: White 50%; African American/Black 20%; Mixed race: 30%. ETHNICITY: Latinx 10% | 40 | both | Yes | Yes |
| 343* | Montes2012 | 254 | 0 | Outpatient | 100 | 0 | 0 | 39.81 (11.0) | NR | 66.52 | n/a (none) | NR | NR |
| 346* | Moritz2015 | 30 | 0 | Mental health services; Support/advocacy/peer groups/charities | 100 | 0 | 0 | 18-65 | NR | 37.07 | n/a (none) | NR | NR |
| 347* | Moritz2016 | 58 | 0 | Mental health services; Support/advocacy/peer groups/charities | 100 | 0 | 0 | 40.62 (10.6) | NR | 46.3 | n/a (none) | NR | NR |
| 348 | Michalak2019#2 | 43 | 0 | Recruitment from other study sample(s); Online; Advertisements; Mental health services | 0 | 100 | 0 | 45-54 | NR | 31 | qualitative | Yes | No |
| 349 | Morton2021b | 919 | 0 | Emails; Social media | 0 | 100 | 0 | 36.9 (12) | White 61%; Black/African 4.4%; South Asian 11.3%; East Asian 2.7%; Southeast Asian 2.5%; Middle Eastern 2.4%; Latin American 5.2%; Other Or Multiple Ethnicities 10.5% | 22.1 | both | Yes | No |
| 350 | Morton2021a | 0 | 80 | Online | 0 | 0 | 0 | n/a | White 11%; South Asian 8%; East Asian 4%; Middle Eastern 8%; Latin American 14% | 46.2 | quantitative | Yes | No |
| 351 | Mueller2018 | 13 | 0 | Psychiatric rehabilitation | 38.5 | 30.8 | 30.8 | 29-63, <50: 46% | White 100% | 24 | both | Yes | Yes |
| 353 | Murnane2016 | 552 | 0 | Online; Support/advocacy/peer groups/charities | 0 | 100 | 0 | 40 (18-35) | NR | 25 | both | Yes | No |
| 355 | Murray2015 | 26 | 0 | Online; Advertisements; Social media | 0 | 100 | 0 | 46.6 (12.9) | NR | 25 | both | Yes | Yes |
| 358 | Naslund2016#2 | 11 | 0 | Community mental health services | 27 | 27 | 46 | 48.2 (11.2) | White 100% | 27 | both | Yes | No |
| 361 | Naslund2016#1 | 11 | 0 | Community mental health services | 27 | 27 | 46 | 48.2 (11.2) | White 100% | 27 | both | Yes | Yes |
| 362 | Proudfoot2012#2 | 358 | 0 | Outpatient; CMHT; GPs; Printed advertisements | 0 | 100 | 0 | 18-29 years: 29%; 30-39 years: 35%; 40-49 years: 24%; 50-59 years: 10%, >60 years: 2% | NR | 30.2 | both | Yes | Yes |
| 364 | Niendam2018 | 76 | 0 | EIS | 64 | 0 | 36 | 18.8 (3.7) | White 54%; African American 28%; Asian American 13%; Caucasian 54%; Native American 1%; Multiple 4%; Hispanic 20% | 66 | quantitative | Yes | Yes |
| 366 | Aref-Adib2016 | 22 | 0 | Community mental health services | 78 | 14 | 9 | 21-57 | White 41%; Black British 18%; African/Caribbean 14%; Asian 5%; Other 23% | 45 | qualitative | Yes | No |
| 369 | Röhricht2021 | 66 | 21 | Community mental health services; EIS | 76 | 21 | 3 | 35.2 (11.16) | White 19.4%; Black African 17.9%; Bangladeshi 16.4%; Pakistani 13.4%; Black Caribbean 10.4%; Indian 7.5%; Black Other 6% | 47.7 | qualitative | Yes | No |
| 370 | Palmier-Claus2012 | 24 | 0 | EIS; CMHT; Inpatient; Assertive outreach; Psychiatric rehabilitation | 100 | 0 | 0 | 36.15 (8.88) | White British 79%; Black British 17%; Asian Pakistani 4% | 75 | quantitative | No | Yes |
| 374 | Peters-Strickland2016 | 67 | 0 | Outpatient | 100 | 0 | 0 | 46.6 (9.7) | RACE: White 17.9%; Black Or African American 76.1%; Other 1.5%. ETHNICITY: Hispanic 4.5% | 74.6 | quantitative | Yes | Yes |
| 375 | Ybarra2019 | 25 | 19 | Community mental health services; Outpatients | 100 | 0 | 0 | <30 years: 12%; 31-40 years: 24%; 41-50 years: 28%; 51-60: years 16%; >61 years: 20% | RACE: White/Caucasian 16%; Black/African-American 80%; Other 4%. ETHNICITY: Hispanic 8% | 40 | qualitative | Yes | No |
| 377 | Poole2012 | 20 | 0 | Recruitment from other study sample(s) | 0 | 100 | 0 | 20-65 | NR | 65 | qualitative | Yes | Yes |
| 380 | Prociow2012 | 1 | 0 | Support/advocacy/peer groups/charities | 0 | 100 | 0 | NR | NR | 0 | qualitative | Yes | No |
| 381 | Proudfoot2012#1 | 419 | 0 | Outpatient; CMHT; GPs; Printed advertisements | 0 | 100 | 0 | 18-29 years: 29%; 30-39 years: 37%; 40-49 years: 23%; 50-59 years: 9%; 60+ years 2% | NR | 30 | quantitative | No | Yes |
| 382 | Puskar2011 | 2 | 0 | Mental health services | 100 | 0 | 0 | 54 (0) | White 50%; African American 50% | 50 | qualitative | Yes | No |
| 384 | Raugh2021 | 54 | 0 | Outpatient | 100 | 0 | 0 | 39.17 (12.41) | White 59%; African American 32%; Biracial 6%; Hispanic/Latino 4% | 35.2 | quantitative | No | Yes |
| 386 | Raymaker2020 | 11 | 0 | EIS | 100 | 0 | 0 | 19-24 | White 50%; Multiracial 50% | 50 | qualitative | Yes | No |
| 387 | Realpe2020 | 20 | 0 | Mental health services; Support/advocacy/peer groups/charities; Email | 100 | 0 | 0 | NR | NR | NR | qualitative | Yes | No |
| 390 | Roberts2017 | 24 | 0 | Community mental health services | 95.8 | 0 | 4.2 | 44.6 (10.7) | White 37.5%; Black 20.83%; Hispanic 41.67% | 58.3 | quantitative | Yes | No |
| 393 | Rodriguez-Villa2021 | 25 | 20 | Inpatient | 100 | 0 | 0 | 33.3 (16-65) | NR | NR | qualitative | Yes | No |
| 397 | Hatch2017 | 0 | 58 | Experts in the field identified from their activity | n/a | n/a | n/a | 59.9 (NR) | NR | NR | quantitative | Yes | No |
| 398 | Rotondi2015 | 38 | 0 | Community mental health services; Psychiatric rehabilitation | 100 | 0 | 0 | 42.7 (6.62) | White 58%; African American 40%; Asian 3% | 50 | quantitative | Yes | Yes |
| 401 | Sablier2012 | 9 | 0 | NR | 100 | 0 | 0 | 36.9 (8.5) | NR | 33 | quantitative | Yes | Yes |
| 404 | Saunders2017 | 21 | 0 | Mental health services; Advertisements | 0 | 100 | 0 | 44.38 (2.49) | NR | 33.3 | both | Yes | No |
| 406* | Achytes2019#3 | 342 | 0 | Community mental health services; Outpatients | 100 | 0 | 0 | 35 (11) | African American 25.2%; Hispanic 10.8%; Asian, American Indian, Native Hawaiian, or more than one race 14.0% | 62.3 | n/a (none) | n/a | n/a |
| 407 | Schlosser2016 | 20 | 0 | EIS; Community mental health services | 100 | 0 | 0 | 23.35 (3.11) | White 30%; African American 20%; Asian 30%; Other 20% | 85 | both | Yes | Yes |
| 412 | Sedgwick2021 | 14 | 0 | Outpatient | 86 | 7 | 7 | 45.1 (9.78) | White 36%; Black, Asian Or Minority Ethnic 64% | 43 | qualitative | Yes | No |
| 414* | Shin2016 | 61 | 0 | Inpatient | 100 | 0 | 0 | 46.59 (8.4) | NR | 57.4 | n/a (none) | NR | NR |
| 415* | Sibeko2017 | 77 | 0 | Outpatient | 80.5 | 14.3 | 5.2 | 35.5 (10.2) | Coloured 66.2%; Black: 25.3%, Other: 8.5% | 71.4 | n/a (none) | NR | NR |
| 418 | Simon2011 | 118 | 0 | Mental health services; Support/advocacy/peer groups/charities; Advertisements; Snowball sampling | 0 | 100 | 0 | NR | White 81%; "Minority Race or Ethnicity" 19% | 28 | quantitative | Yes | Yes |
| 424* | Spaniel2012 | 146 | 0 | NR | 100 | 0 | 0 | 33.03 (10.0) | NR | 56.17 | n/a (none) | NR | NR |
| 425* | Spaniel2015 | 146 | 0 | Outpatient | 100 | 0 | 0 | 36.45 (9.5) | NR | 56.12 | n/a (none) | NR | NR |
| 427 | Sreejith2019 | 75 | 0 | Outpatients | 50.7 | 49.3 | 0 | 38.1 (11.3) | NR | 49.3 | quantitative | Yes | No |
| 429 | Steare2020#1 | 80 | 0 | EIS | 80.6 | 0 | 19.4 | 29.7 (9.78) | White British 35%; Any Other White/Mixed White 7.5%; Black African 20%; Black Caribbean 5%; Black Other 2.5%; Asian Indian 2.5%; Asian Other 7.5%; Other/Mixed Other 15% | 70 | both | Yes | Yes |
| 430 | Steare2020#2 | 21 | 13 | EIS | 66.7 | 0 | 33.3 | 29.8 (18-52) | White British 42.3%; Other White/Mixed White 14.3%; Black 19.1%; Asuab 9.5%; Other/Mixed Other 14.3% | 76.2 | qualitative | Yes | No |
| 434 | Storm2021 | 5 | 6 | Community mental health services | NR | NR | NR | 20-60 | NR | 60 | qualitative | Yes | Yes |
| 438 | Swartz2021 | 47 | 0 | Outpatients; Advertisements | 0 | 100 | 0 | 41.8 (13.5) | White 66%; African American 27.7%; Other 6.3% | 26 | quantitative | Yes | Yes |
| 440 | Switsers2018 | 16 | 0 | Support/advocacy/peer groups/charities | 0 | 100 | 0 | 42 (14) | NR | 43.75 | qualitative | Yes | No |
| 443 | Terp2018 | 13 | 0 | EIS | 100 | 0 | 0 | 24.8 (18-36) | NR | 31 | qualitative | Yes | No |
| 444 | Lal2018 | 17 | 0 | Community mental health services | NR | NR | NR | 28.5 (4.8) | White 82.3%; Asian 11.8%; Black 5.9% | 64.7 | qualitative | Yes | No |
| 445 | Thomas2017 | 100 | 0 | Mental health services | NR | NR | NR | 18–24 years: 1%; 25–34 years: 27%; 35–44 years: 33%; 45–54 years: 22%; 55–64 years: 16 % | NR | 57 | quantitative | Yes | No |
| 446 | Wilson2019 | 13 | 0 | Outpatient; Support/advocacy/peer groups/charities; Advertisements | 100 | 0 | 0 | 47.8 (11) | White 8%; Black Or African American 77%; Multiracial 15% | 62 | qualitative | Yes | No |
| 447 | Thonon2022 | 8 | 0 | Inpatient; Community mental health services | 87.5 | 0 | 12.5 | 33.63 (9.2) | NR | NR | quantitative | No | Yes |
| 450 | Todd2013 | 12 | 0 | Websites, support/advocacy/peer groups/charities | 0 | 100 | 0 | 41.5 (8.8) | White British 92%; Other ethnicity 8.3% | 58.3 | qualitative | Yes | No |
| 452 | Tolley2015 | 12 | 0 | Referrals from private physicians | 100 | 0 | 0 | 47 (23-83) | White 25%; Black/African American 75% | 50 | qualitative | Yes | No |
| 453 | Torous2017 | 1 | 0 | n/a | 100 | 0 | 0 | 28 (0) | NR | 100 | qualitative | Yes | No |
| 455 | Torous2018 | 72 | 0 | Outpatients | 100 | 0 | 0 | 35.4 (NR) | NR | 45.8 | quantitative | Yes | Yes |
| 463 | Vaessen2019 | 16 | 0 | NR | NR | NR | NR | NR | NR | NR | quantitative | Yes | No |
| 464 | Vaidyam2020 | 1 | 0 | NR | 100 | 0 | 0 | 30-39 | NR | 100 | qualitative | Yes | No |
| 468 | Valentine2020 | 10 | 0 | EIS | 100 | 0 | 0 | 23.4 (2.6) | NR | 30 | qualitative | Yes | No |
| 469 | Alvarez-Jimenez2021#3 | 12 | 0 | EIS | NR | NR | NR | 23.2 (2.6) | NR | 42 | qualitative | Yes | No |
| 472 | Välimäki2017#1 | 1139 | 0 | Inpatient | 39.9 | 29.4 | 30.7 | 38.2 (12.5) | NR | 49.2 | quantitative | Yes | Yes |
| 474 | vandenHeuvel2018 | 66 | 11 | Outpatient | 0 | 100 | 0 | 45.17 (10.67) | NR | 33.3 | quantitative | Yes | Yes |
| 475 | vanderKrieke2012 | 15 | 0 | Outpatients | 100 | 0 | 0 | 42 (23-61) | NR | 66.6 | qualitative | Yes | No |
| 476* | vanderKrieke2013 | 73 | 0 | Outpatient | NR | NR | NR | 38.51 (13.0) | NR | 59.44 | n/a (none) | NR | NR |
| 478 | VanTil2020 | 47 | 0 | Recruitment from other study sample(s) | 0 | 100 | 0 | 41.9 (10.8) | White 85.1%; Black Or African American 6.3%; Asian 4.3; American Indian Or Alaskan Native 2.2%; Mixed race 2.2% [Hispanic Vs. Non-Hispanic ETHNICITY Also Reported But Inconsistently] | 46.9 | quantitative | Yes | Yes |
| 483 | Vilardaga2019 | 7 | 0 | Mental health services | 42.8 | 57.1 | 0 | 45 (9.5) | White 71.4%; Mixed race 28.6% | 42.8 | both | Yes | Yes |
| 489 | Wenze2016 | 8 | 0 | Inpatient; Outpatient | 0 | 100 | 0 | 44 (11.58) | White 87.5; White And Hispanic 12.5% | 37.5 | both | Yes | Yes |
| 491 | Williams2018 | 0 | 37 | Mental health services | n/a | n/a | n/a | n/a | NR | 13 | qualitative | Yes | No |
| 492 | Thomas2016#2 | 36 | 0 | Community mental health services; Psychiatric rehabilitation | NR | NR | NR | 41 (19-64) | NR | 33 | qualitative | Yes | No |
| 493 | Hanssen2020#2 | 37 | 15 | Mental health services | NR | NR | NR | 18-64 | NR | 35.1 | qualitative | Yes | No |
| 497 | Brunette2020 | 162 | 0 | Outpatient | 100 | 0 | 0 | 45.9 (11.3) | RACE: White 29.0%; Black 53.1%; Mixed And Other 17.9%. ETHNICITY: Hispanic 13% | 66.7 | quantitative | Yes | No |
| 498 | Xu2019#1 | 278 | 0 | Community mental health services | 100 | 0 | 0 | 46 (12.7) | NR | 51.8 | quantitative | No | Yes |
| 499* | Xu2020 | 142 | 0 | Online; In person | NR | NR | NR | 19-29: 17%, 30-39: 37%, 40-49: 26%, >50: 20% | NR | 39.4 | n/a (none) | NR | NR |
| 502* | Zhu2020 | 84 | 0 | Previous inpatients | 100 | 0 | 0 | 31.51 (7.1) | NR | 49.32 | n/a (none) | NR | NR |

*Excluded from main review; included only in supplementary review of safety reporting in studies testing actual DHI use (Supplementary Results SR1; Supplementary Table S5). See Table S1 for full paper reference.

**Type of user engagement indicator described in the paper in relation to factors affecting engagement. Example subjective user indicators: qualitative interviews, survey data, expert consensus data, reasons for dropout. Example objective user indicators: number of logins to a website, percentage of digital symptom monitoring prompts completed, number of participants stopping DHI, smartwatch wear time.

Abbreviations: SU, service user; HCP, Health Care Professional; SSD, schizophrenia spectrum disorder

## Supplementary Table S4. Mixed Methods Appraisal Tool (MMAT) Quality checks

|  | | | | **Criteria from the Mixed Methods Appraisal Tool*** | | | | | | | | | | | | | | | | | | | | | | | | | | | | | | | | | | | | | | | | | | | | |  |  |  |
| --- | --- | --- | --- | --- | --- | --- | --- | --- | --- | --- | --- | --- | --- | --- | --- | --- | --- | --- | --- | --- | --- | --- | --- | --- | --- | --- | --- | --- | --- | --- | --- | --- | --- | --- | --- | --- | --- | --- | --- | --- | --- | --- | --- | --- | --- | --- | --- | --- | --- | --- | --- |
| **Study identifier:**  **Lead author, year** | | | | **1.1** | | **1.2** | | **1.3** | | **1.4** | **1.5** | | **2.1** | | **2.2** | | **2.3** | | **2.4** | **2.5** | | **3.1** | | **3.2** | | **3.3** | | **3.4** | **3.5** | | **4.1** | | **4.2** | | **4.3** | | **4.4** | **4.5** | | **5.1** | | **5.2** | | **5.3** | | **5.4** | **5.5** | |  |  |  |
| Achytes2019#1 | | | |  | |  | |  | |  |  | |  | |  | |  | |  |  | | 1 | | 1 | | 1 | | 1 | 1 | |  | |  | |  | |  |  | |  | |  | |  | |  |  | |  |  |  |
| Achytes2019#2 | | | |  | |  | |  | |  |  | |  | |  | |  | |  |  | | 1 | | 1 | | 1 | | 1 | 1 | |  | |  | |  | |  |  | |  | |  | |  | |  |  | |  |  |  |
| Ainsworth2013#1 | | | |  | |  | |  | |  |  | | 1 | | 1 | | 1 | | 0 | 1 | |  | |  | |  | |  |  | |  | |  | |  | |  |  | |  | |  | |  | |  |  | |  |  |  |
| Ainsworth2013#2 | | | | 1 | | 1 | | 1 | | 1 | 1 | |  | |  | |  | |  |  | |  | |  | |  | |  |  | |  | |  | |  | |  |  | |  | |  | |  | |  |  | |  |  |  |
| Allan2019 | | | | 1 | | 1 | | 1 | | 1 | 1 | |  | |  | |  | |  |  | |  | |  | |  | |  |  | |  | |  | |  | |  |  | |  | |  | |  | |  |  | |  |  |  |
| Alvarez-Jimenez2021#1 | | | |  | |  | |  | |  |  | | 1 | | 1 | | 1 | | 1 | 1 | |  | |  | |  | |  |  | |  | |  | |  | |  |  | |  | |  | |  | |  |  | |  |  |  |
| Alvarez-Jimenez2021#2 | | | | 1 | | 1 | | 1 | | 1 | 1 | |  | |  | |  | |  |  | |  | |  | |  | |  |  | |  | |  | |  | |  |  | |  | |  | |  | |  |  | |  |  |  |
| Alvarez-Jimenez2021#3 | | | | 1 | | 1 | | 1 | | 1 | 1 | |  | |  | |  | |  |  | |  | |  | |  | |  |  | |  | |  | |  | |  |  | |  | |  | |  | |  |  | |  |  |  |
| Aref-Adib2016 | | | | 1 | | 1 | | 1 | | 1 | 1 | |  | |  | |  | |  |  | |  | |  | |  | |  |  | |  | |  | |  | |  |  | |  | |  | |  | |  |  | |  |  |  |
| Aschbrenner2016 | | | | 1 | | 1 | | 1 | | 1 | 1 | |  | |  | |  | |  |  | |  | |  | |  | |  |  | | 1 | | 1 | | 1 | | 1 | 1 | | 1 | | 1 | | 1 | | 1 | 1 | |  |  |  |
| Austin2021 | | | | 1 | | 1 | | 1 | | 1 | 1 | |  | |  | |  | |  |  | |  | |  | |  | |  |  | | 1 | | 1 | | 1 | | 0 | 1 | | 1 | | 1 | | 1 | | 1 | 1 | |  |  |  |
| Barnes2011 | | | | 1 | | 1 | | 1 | | 1 | 1 | |  | |  | |  | |  |  | |  | |  | |  | |  |  | |  | |  | |  | |  |  | |  | |  | |  | |  |  | |  |  |  |
| Baumel2016a | | | | 1 | | 1 | | u | | u | 1 | |  | |  | |  | |  |  | | 1 | | 1 | | 1 | | u | 1 | |  | |  | |  | |  |  | | 0 | | 0 | | u | | u | 1 | |  |  |  |
| Baumel2016b | | | |  | |  | |  | |  |  | |  | |  | |  | |  |  | |  | |  | |  | |  |  | | 1 | | 1 | | 1 | | 1 | 1 | |  | |  | |  | |  |  | |  |  |  |
| Bechtel2021 | | | | 1 | | 1 | | 1 | | 1 | 1 | |  | |  | |  | |  |  | |  | |  | |  | |  |  | | 1 | | 0 | | 1 | | 0 | 1 | | 1 | | 1 | | 0 | | 1 | 1 | |  |  |  |
| Bell2018 | | | | 1 | | u | | u | | 0 | 0 | |  | |  | |  | |  |  | |  | |  | |  | |  |  | |  | |  | |  | |  |  | |  | |  | |  | |  |  | |  |  |  |
| Bell2020 | | | | 1 | | 1 | | 1 | | 1 | 1 | | 1 | | 1 | | 1 | | 1 | 1 | |  | |  | |  | |  |  | |  | |  | |  | |  |  | | 1 | | 1 | | 1 | | 1 | 1 | |  |  |  |
| Ben-Zeev2013 | | | | 0 | | 0 | | 0 | | 0 | u | |  | |  | |  | |  |  | |  | |  | |  | |  |  | | 1 | | 1 | | 0 | | 1 | 1 | | 0 | | 0 | | 0 | | 0 | 0 | |  |  |  |
| Ben-Zeev2014b | | | |  | |  | |  | |  |  | |  | |  | |  | |  |  | | 1 | | 1 | | 1 | | 1 | 1 | |  | |  | |  | |  |  | |  | |  | |  | |  |  | |  |  |  |
| Ben-Zeev2016 | | | |  | |  | |  | |  |  | |  | |  | |  | |  |  | | 0 | | 0 | | 0 | | 0 | 1 | |  | |  | |  | |  |  | |  | |  | |  | |  |  | |  |  |  |
| Ben-Zeev2018a | | | | 1 | | 1 | | 1 | | 1 | 1 | |  | |  | |  | |  |  | |  | |  | |  | |  |  | | 1 | | 0 | | 1 | | 0 | 1 | | 1 | | 1 | | 1 | | 1 | 1 | |  |  |  |
| Ben-Zeev2018b#1 | | | |  | |  | |  | |  |  | | 1 | | 1 | | 0 | | 1 | 1 | |  | |  | |  | |  |  | |  | |  | |  | |  |  | |  | |  | |  | |  |  | |  |  |  |
| Ben-Zeev2018b#2 | | | |  | |  | |  | |  |  | | 1 | | 1 | | 1 | | 1 | 1 | | 1 | | 1 | | 0 | | 0 | 1 | |  | |  | |  | |  |  | |  | |  | |  | |  |  | |  |  |  |
| Ben-Zeev2018b#3 | | | | 1 | | 1 | | 1 | | 1 | 1 | |  | |  | |  | |  |  | |  | |  | |  | |  |  | |  | |  | |  | |  |  | |  | |  | |  | |  |  | |  |  |  |
| Ben-Zeev2018b#4 | | | | 1 | | 1 | | 1 | | 1 | 1 | |  | |  | |  | |  |  | |  | |  | |  | |  |  | |  | |  | |  | |  |  | |  | |  | |  | |  |  | |  |  |  |
| Ben-Zeev2021 | | | |  | |  | |  | |  |  | | 1 | | 1 | | 0 | | u | 0 | |  | |  | |  | |  |  | |  | |  | |  | |  |  | |  | |  | |  | |  |  | |  |  |  |
| Berry2017 | | | | 1 | | 1 | | 1 | | 1 | 1 | |  | |  | |  | |  |  | |  | |  | |  | |  |  | |  | |  | |  | |  |  | |  | |  | |  | |  |  | |  |  |  |
| Berry2019 | | | | 1 | | 1 | | 1 | | 1 | 1 | |  | |  | |  | |  |  | |  | |  | |  | |  |  | |  | |  | |  | |  |  | |  | |  | |  | |  |  | |  |  |  |
| Berry2021 | | | | 1 | | 1 | | 1 | | 1 | 1 | |  | |  | |  | |  |  | | 1 | | 1 | | 1 | | 1 | 1 | |  | |  | |  | |  |  | | 1 | | 1 | | 1 | | 1 | 1 | |  |  |  |
| Biagianti2016 | | | |  | |  | |  | |  |  | |  | |  | |  | |  |  | | 0 | | 1 | | 1 | | 1 | 1 | |  | |  | |  | |  |  | |  | |  | |  | |  |  | |  |  |  |
| Bonet2018 | | | |  | |  | |  | |  |  | |  | |  | |  | |  |  | |  | |  | |  | |  |  | | 1 | | 0 | | 1 | | 1 | 1 | |  | |  | |  | |  |  | |  |  |  |
| Bonet2020 | | | |  | |  | |  | |  |  | |  | |  | |  | |  |  | |  | |  | |  | |  |  | | 1 | | 1 | | 1 | | 1 | 1 | |  | |  | |  | |  |  | |  |  |  |
| Bopp2010 | | | |  | |  | |  | |  |  | |  | |  | |  | |  |  | | 1 | | 1 | | 1 | | 1 | 1 | |  | |  | |  | |  |  | |  | |  | |  | |  |  | |  |  |  |
| Bos2020 | | | | 1 | | 1 | | 1 | | 1 | 1 | |  | |  | |  | |  |  | |  | |  | |  | |  |  | |  | |  | |  | |  |  | |  | |  | |  | |  |  | |  |  |  |
| Bowden2021 | | | | 1 | | 1 | | 1 | | 0 | 0 | |  | |  | |  | |  |  | |  | |  | |  | |  |  | | 1 | | 0 | | 1 | | 1 | 1 | | 0 | | 0 | | 0 | | 0 | 1 | |  |  |  |
| Brunette2016 | | | | u | | u | | u | | u | u | |  | |  | |  | |  |  | |  | |  | |  | |  |  | | 1 | | 1 | | 1 | | 1 | 1 | | 1 | | u | | 0 | | 0 | 0 | |  |  |  |
| Brunette2019 | | | | 1 | | 1 | | 1 | | 1 | u | |  | |  | |  | |  |  | |  | |  | |  | |  |  | | 1 | | 0 | | 1 | | 1 | 1 | | 1 | | 1 | | u | | 1 | u | |  |  |  |
| Brunette2020 | | | |  | |  | |  | |  |  | | 1 | | 1 | | 1 | | 1 | 1 | |  | |  | |  | |  |  | |  | |  | |  | |  |  | |  | |  | |  | |  |  | |  |  |  |
| Bucci2018a | | | | 1 | | 1 | | 1 | | 1 | 1 | | 1 | | 1 | | 0 | | 1 | 1 | |  | |  | |  | |  |  | |  | |  | |  | |  |  | | 0 | | 0 | | 0 | | 0 | 1 | |  |  |  |
| Bucci2018b | | | | 1 | | 1 | | 1 | | 1 | 1 | |  | |  | |  | |  |  | |  | |  | |  | |  |  | |  | |  | |  | |  |  | |  | |  | |  | |  |  | |  |  |  |
| Bucci2019a#1 | | | | u | | u | | u | | u | u | |  | |  | |  | |  |  | | 1 | | 1 | | 1 | | 1 | 1 | |  | |  | |  | |  |  | | 0 | | 0 | | 0 | | 0 | 0 | |  |  |  |
| Bucci2019a#2 | | | | 1 | | 1 | | u | | 0 | u | |  | |  | |  | |  |  | |  | |  | |  | |  |  | |  | |  | |  | |  |  | |  | |  | |  | |  |  | |  |  |  |
| Bucci2019b | | | | 1 | | 1 | | 1 | | 1 | 1 | |  | |  | |  | |  |  | |  | |  | |  | |  |  | |  | |  | |  | |  |  | |  | |  | |  | |  |  | |  |  |  |
| Camacho2021 | | | |  | |  | |  | |  |  | |  | |  | |  | |  |  | |  | |  | |  | |  |  | | 1 | | 1 | | 1 | | 1 | 1 | |  | |  | |  | |  |  | |  |  |  |
| Chiauzzi2019 | | | | 1 | | 1 | | 1 | | 1 | 1 | |  | |  | |  | |  |  | |  | |  | |  | |  |  | |  | |  | |  | |  |  | |  | |  | |  | |  |  | |  |  |  |
| Cho2020 | | | |  | |  | |  | |  |  | |  | |  | |  | |  |  | | 1 | | 1 | | 1 | | 1 | 1 | |  | |  | |  | |  |  | |  | |  | |  | |  |  | |  |  |  |
| Daus2018 | | | | 1 | | 1 | | 1 | | 1 | 0 | |  | |  | |  | |  |  | |  | |  | |  | |  |  | | 1 | | 1 | | 1 | | 0 | 1 | | 1 | | 1 | | 1 | | 1 | 1 | |  |  |  |
| deAlmeida2019 | | | | 1 | | 1 | | 0 | | 0 | 0 | |  | |  | |  | |  |  | | 0 | | 1 | | 1 | | 0 | 1 | | 1 | | 1 | | 1 | | u | 1 | | 1 | | 1 | | 1 | | 0 | u | |  |  |  |
| Depp2021 | | | |  | |  | |  | |  |  | |  | |  | |  | |  |  | | 1 | | 1 | | 0 | | 1 | 1 | |  | |  | |  | |  |  | |  | |  | |  | |  |  | |  |  |  |
| Depp2015#1 | | | |  | |  | |  | |  |  | | 1 | | 1 | | 1 | | 1 | 0 | |  | |  | |  | |  |  | |  | |  | |  | |  |  | |  | |  | |  | |  |  | |  |  |  |
| Depp2015#2 | | | | 0 | | u | | u | | 0 | 1 | |  | |  | |  | |  |  | | 1 | | 1 | | 1 | | 1 | 1 | |  | |  | |  | |  |  | | 0 | | 0 | | 1 | | 0 | 0 | |  |  |  |
| Eisner2019#1 | | | | 1 | | 1 | | 1 | | 1 | 1 | |  | |  | |  | |  |  | | 1 | | 1 | | 1 | | 1 | 1 | |  | |  | |  | |  |  | | 0 | | 0 | | u | | u | 1 | |  |  |  |
| Eisner2019#2 | | | | 1 | | 1 | | 1 | | 1 | 1 | |  | |  | |  | |  |  | |  | |  | |  | |  |  | | 1 | | 1 | | 1 | | 1 | 1 | | 1 | | 1 | | 1 | | 1 | 1 | |  |  |  |
| Enrique2020 | | | | 1 | | 1 | | 1 | | 1 | 0 | |  | |  | |  | |  |  | | 0 | | 1 | | 0 | | 0 | 1 | |  | |  | |  | |  |  | | 1 | | 1 | | 1 | | 1 | 1 | |  |  |  |
| Faurholt-Jepsen2019 | | | |  | |  | |  | |  |  | | 1 | | 0 | | 1 | | 1 | 1 | |  | |  | |  | |  |  | |  | |  | |  | |  |  | |  | |  | |  | |  |  | |  |  |  |
| Faurholt-Jepsen2020 | | | |  | |  | |  | |  |  | | 1 | | 1 | | 1 | | 1 | 1 | |  | |  | |  | |  |  | |  | |  | |  | |  |  | |  | |  | |  | |  |  | |  |  |  |
| Fellendorf2021 | | | |  | |  | |  | |  |  | |  | |  | |  | |  |  | | 1 | | 1 | | 0 | | 0 | 0 | |  | |  | |  | |  |  | |  | |  | |  | |  |  | |  |  |  |
| Ferreira-Correia2018 | | | | 1 | | 1 | | 1 | | 1 | 1 | |  | |  | |  | |  |  | |  | |  | |  | |  |  | |  | |  | |  | |  |  | |  | |  | |  | |  |  | |  |  |  |
| Ferron2012 | | | |  | |  | |  | |  |  | |  | |  | |  | |  |  | | 1 | | 1 | | 1 | | 1 | 1 | |  | |  | |  | |  |  | |  | |  | |  | |  |  | |  |  |  |
| Fletcher2021 | | | | 1 | | 1 | | 0 | | 1 | 0 | |  | |  | |  | |  |  | |  | |  | |  | |  |  | | 1 | | 0 | | 1 | | 0 | 1 | | 1 | | 1 | | 1 | | 1 | 1 | |  |  |  |
| Forchuk2015#1 | | | | 1 | | 1 | | 1 | | 1 | 1 | |  | |  | |  | |  |  | |  | |  | |  | |  |  | |  | |  | |  | |  |  | |  | |  | |  | |  |  | |  |  |  |
| Forchuk2015#2 | | | | 1 | | 1 | | 1 | | 1 | 1 | |  | |  | |  | |  |  | | u | | 1 | | 1 | | 1 | 1 | |  | |  | |  | |  |  | | 1 | | 1 | | 1 | | 1 | 1 | |  |  |  |
| Fowler2021 | | | |  | |  | |  | |  |  | |  | |  | |  | |  |  | |  | |  | |  | |  |  | | 1 | | 1 | | 1 | | 0 | 1 | |  | |  | |  | |  |  | |  |  |  |
| Fulford2020 | | | | 0 | | 1 | | u | | 0 | u | |  | |  | |  | |  |  | |  | |  | |  | |  |  | | 0 | | 0 | | 1 | | 0 | 1 | | 0 | | 1 | | 1 | | u | 0 | |  |  |  |
| Gorczynski2014 | | | |  | |  | |  | |  |  | |  | |  | |  | |  |  | | 0 | | 1 | | 1 | | 0 | 1 | |  | |  | |  | |  |  | |  | |  | |  | |  |  | |  |  |  |
| Gordon-Smith2019 | | | | 1 | | 1 | | u | | u | 0 | |  | |  | |  | |  |  | | 0 | | 1 | | 0 | | 0 | 1 | |  | |  | |  | |  |  | | 1 | | 0 | | 0 | | 0 | 0 | |  |  |  |
| Granholm2012 | | | |  | |  | |  | |  |  | |  | |  | |  | |  |  | | 1 | | 1 | | 1 | | 1 | 1 | |  | |  | |  | |  |  | |  | |  | |  | |  |  | |  |  |  |
| Hanssen2020#1 | | | |  | |  | |  | |  |  | | u | | 1 | | 1 | | u | 1 | |  | |  | |  | |  |  | |  | |  | |  | |  |  | |  | |  | |  | |  |  | |  |  |  |
| Hanssen2020#2 | | | | 1 | | 1 | | 1 | | 1 | 1 | |  | |  | |  | |  |  | |  | |  | |  | |  |  | |  | |  | |  | |  |  | |  | |  | |  | |  |  | |  |  |  |
| Hanssen2020#3 | | | |  | |  | |  | |  |  | | 1 | | 1 | | 1 | | u | 1 | |  | |  | |  | |  |  | |  | |  | |  | |  |  | |  | |  | |  | |  |  | |  |  |  |
| Hanssen2020#4 | | | | 1 | | 1 | | 1 | | 1 | 1 | |  | |  | |  | |  |  | |  | |  | |  | |  |  | |  | |  | |  | |  |  | |  | |  | |  | |  |  | |  |  |  |
| Hardy2018 | | | | 1 | | 1 | | 1 | | 1 | 1 | |  | |  | |  | |  |  | | 1 | | 1 | | 1 | | u | 1 | |  | |  | |  | |  |  | | 1 | | 1 | | 1 | | u | 1 | |  |  |  |
| Harris2017 | | | |  | |  | |  | |  |  | | 1 | | 1 | | 0 | | 1 | 0 | |  | |  | |  | |  |  | |  | |  | |  | |  |  | |  | |  | |  | |  |  | |  |  |  |
| Hatch2017 | | | |  | |  | |  | |  |  | |  | |  | |  | |  |  | |  | |  | |  | |  |  | | 1 | | 0 | | 1 | | 1 | 1 | |  | |  | |  | |  |  | |  |  |  |
| Hatch2018 | | | |  | |  | |  | |  |  | |  | |  | |  | |  |  | |  | |  | |  | |  |  | | 1 | | 0 | | 1 | | 0 | 1 | |  | |  | |  | |  |  | |  |  |  |
| Hidalgo-Mazzei2016#1 | | | |  | |  | |  | |  |  | |  | |  | |  | |  |  | | 1 | | 1 | | 1 | | 1 | 1 | |  | |  | |  | |  |  | |  | |  | |  | |  |  | |  |  |  |
| Hidalgo-Mazzei2016#2 | | | |  | |  | |  | |  |  | |  | |  | |  | |  |  | | 1 | | 1 | | 0 | | 1 | 1 | |  | |  | |  | |  |  | |  | |  | |  | |  |  | |  |  |  |
| Hidalgo-Mazzei2016#3 | | | |  | |  | |  | |  |  | |  | |  | |  | |  |  | | 0 | | 1 | | 1 | | 0 | 1 | |  | |  | |  | |  |  | |  | |  | |  | |  |  | |  |  |  |
| Hidalgo-Mazzei2018 | | | |  | |  | |  | |  |  | |  | |  | |  | |  |  | | 1 | | 1 | | 0 | | 1 | 1 | |  | |  | |  | |  |  | |  | |  | |  | |  |  | |  |  |  |
| Huerta-Ramos2016#1 | | | | 1 | | 1 | | 1 | | 1 | 1 | |  | |  | |  | |  |  | |  | |  | |  | |  |  | |  | |  | |  | |  |  | |  | |  | |  | |  |  | |  |  |  |
| Huerta-Ramos2016#2 | | | | 1 | | 1 | | 1 | | 1 | 1 | |  | |  | |  | |  |  | |  | |  | |  | |  |  | |  | |  | |  | |  |  | |  | |  | |  | |  |  | |  |  |  |
| Jonathan2021 | | | | 1 | | 1 | | 1 | | 1 | 1 | |  | |  | |  | |  |  | | 1 | | 1 | | 1 | | 1 | 1 | |  | |  | |  | |  |  | | 1 | | 1 | | 1 | | 1 | 1 | |  |  |  |
| Kane2013 | | | |  | |  | |  | |  |  | |  | |  | |  | |  |  | | 1 | | 1 | | 1 | | 1 | 1 | |  | |  | |  | |  |  | |  | |  | |  | |  |  | |  |  |  |
| Kidd2019 | | | | 1 | | 1 | | 1 | | 1 | 1 | |  | |  | |  | |  |  | | 0 | | 1 | | 1 | | 1 | 1 | |  | |  | |  | |  |  | | 0 | | 0 | | u | | 0 | 1 | |  |  |  |
| Kopelowicz2017 | | | |  | |  | |  | |  |  | |  | |  | |  | |  |  | | 0 | | 1 | | 0 | | 1 | 1 | |  | |  | |  | |  |  | |  | |  | |  | |  |  | |  |  |  |
| Kreyenbuhl2019 | | | |  | |  | |  | |  |  | |  | |  | |  | |  |  | |  | |  | |  | |  |  | | 1 | | 0 | | 1 | | 1 | 1 | |  | |  | |  | |  |  | |  |  |  |
| Laine2019 | | | |  | |  | |  | |  |  | |  | |  | |  | |  |  | | 1 | | 1 | | 0 | | 0 | 1 | |  | |  | |  | |  |  | |  | |  | |  | |  |  | |  |  |  |
| Lal2015 | | | |  | |  | |  | |  |  | |  | |  | |  | |  |  | |  | |  | |  | |  |  | | 1 | | 0 | | 1 | | 1 | 1 | |  | |  | |  | |  |  | |  |  |  |
| Lal2018 | | | | 1 | | 1 | | 1 | | 1 | 1 | |  | |  | |  | |  |  | |  | |  | |  | |  |  | |  | |  | |  | |  |  | |  | |  | |  | |  |  | |  |  |  |
| Lederman2014 | | | | 0 | | 0 | | u | | 1 | 1 | |  | |  | |  | |  |  | |  | |  | |  | |  |  | |  | |  | |  | |  |  | |  | |  | |  | |  |  | |  |  |  |
| Lewandowski2021 | | | | 1 | | 1 | | 1 | | 0 | 1 | |  | |  | |  | |  |  | |  | |  | |  | |  |  | | 1 | | 1 | | 1 | | 1 | 1 | | 0 | | 0 | | 0 | | 0 | 1 | |  |  |  |
| Lim2020 | | | | 1 | | 1 | | u | | 1 | 0 | |  | |  | |  | |  |  | | 1 | | 1 | | 1 | | 0 | 1 | |  | |  | |  | |  |  | | 1 | | 0 | | 1 | | 0 | 0 | |  |  |  |
| Lobban2017#1 | | | | 1 | | 1 | | 1 | | 1 | 1 | | 1 | | 1 | | 1 | | 1 | 1 | |  | |  | |  | |  |  | |  | |  | |  | |  |  | | 1 | | 1 | | 1 | | 1 | 1 | |  |  |  |
| Lobban2017#2 | | | | 1 | | 1 | | 1 | | 1 | 1 | |  | |  | |  | |  |  | |  | |  | |  | |  |  | |  | |  | |  | |  |  | |  | |  | |  | |  |  | |  |  |  |
| Lopez-Morinigo2021 | | | |  | |  | |  | |  |  | |  | |  | |  | |  |  | | 1 | | 1 | | 0 | | 1 | 1 | |  | |  | |  | |  |  | |  | |  | |  | |  |  | |  |  |  |
| Ludwig2021 | | | |  | |  | |  | |  |  | |  | |  | |  | |  |  | | 0 | | 1 | | 1 | | 0 | 1 | |  | |  | |  | |  |  | |  | |  | |  | |  |  | |  |  |  |
| Luther2020 | | | |  | |  | |  | |  |  | | 1 | | 1 | | 1 | | 1 | 1 | |  | |  | |  | |  |  | |  | |  | |  | |  |  | |  | |  | |  | |  |  | |  |  |  |
| Matthews2017 | | | | 1 | | 1 | | 1 | | 1 | 1 | |  | |  | |  | |  |  | |  | |  | |  | |  |  | |  | |  | |  | |  |  | |  | |  | |  | |  |  | |  |  |  |
| McClelland2018 | | | | 1 | | 1 | | 1 | | 1 | 1 | |  | |  | |  | |  |  | |  | |  | |  | |  |  | |  | |  | |  | |  |  | |  | |  | |  | |  |  | |  |  |  |
| McKnight2017 | | | |  | |  | |  | |  |  | |  | |  | |  | |  |  | | 0 | | 1 | | 1 | | 1 | 1 | |  | |  | |  | |  |  | |  | |  | |  | |  |  | |  |  |  |
| Medalia2021 | | | | 1 | | 1 | | 1 | | 1 | 1 | | u | | 1 | | 0 | | 1 | u | |  | |  | |  | |  |  | |  | |  | |  | |  |  | | 1 | | 1 | | 1 | | 1 | 1 | |  |  |  |
| Medenblik2020 | | | |  | |  | |  | |  |  | | 1 | | 0 | | u | | 1 | u | |  | |  | |  | |  |  | |  | |  | |  | |  |  | |  | |  | |  | |  |  | |  |  |  |
| Merzenich2014 | | | |  | |  | |  | |  |  | |  | |  | |  | |  |  | | 1 | | 1 | | 1 | | 0 | 1 | |  | |  | |  | |  |  | |  | |  | |  | |  |  | |  |  |  |
| Meyer2018 | | | |  | |  | |  | |  |  | |  | |  | |  | |  |  | |  | |  | |  | |  |  | | 1 | | 1 | | 1 | | 1 | 1 | |  | |  | |  | |  |  | |  |  |  |
| Michalak2019#1 | | | | 1 | | 1 | | 1 | | 1 | 1 | |  | |  | |  | |  |  | | 0 | | 1 | | 1 | | 0 | 1 | |  | |  | |  | |  |  | | 1 | | 1 | | 1 | | 1 | 1 | |  |  |  |
| Michalak2019#2 | | | | 1 | | 1 | | 1 | | 1 | 1 | |  | |  | |  | |  |  | |  | |  | |  | |  |  | |  | |  | |  | |  |  | |  | |  | |  | |  |  | |  |  |  |
| Miller2015 | | | |  | |  | |  | |  |  | |  | |  | |  | |  |  | |  | |  | |  | |  |  | | 1 | | 0 | | 1 | | 1 | 1 | |  | |  | |  | |  |  | |  |  |  |
| Mistler2017 | | | | 1 | | 1 | | 1 | | 1 | 1 | |  | |  | |  | |  |  | |  | |  | |  | |  |  | | 1 | | 0 | | 1 | | 0 | 1 | | 1 | | 1 | | 1 | | 0 | 1 | |  |  |  |
| Moitra2017 | | | | 1 | | u | | u | | 1 | 1 | |  | |  | |  | |  |  | | 1 | | 1 | | 1 | | 1 | 1 | |  | |  | |  | |  |  | | 0 | | 0 | | 0 | | 0 | 0 | |  |  |  |
| Moitra2021 | | | | 0 | | u | | u | | 1 | u | |  | |  | |  | |  |  | | 1 | | 1 | | 0 | | 0 | 1 | |  | |  | |  | |  |  | | 0 | | 0 | | u | | 0 | 0 | |  |  |  |
| Morton2021a | | | |  | |  | |  | |  |  | |  | |  | |  | |  |  | |  | |  | |  | |  |  | | 1 | | u | | 1 | | 0 | 1 | |  | |  | |  | |  |  | |  |  |  |
| Morton2021b | | | | 1 | | 1 | | 1 | | 1 | 1 | |  | |  | |  | |  |  | |  | |  | |  | |  |  | | 1 | | 1 | | 1 | | 1 | 1 | | 1 | | 1 | | 1 | | 1 | 0 | |  |  |  |
| Moura2019 | | | |  | |  | |  | |  |  | |  | |  | |  | |  |  | | 1 | | 1 | | 0 | | 1 | 1 | |  | |  | |  | |  |  | |  | |  | |  | |  |  | |  |  |  |
| Mueller2018 | | | | 0 | | u | | u | | u | 1 | |  | |  | |  | |  |  | | 1 | | 1 | | 0 | | u | 1 | |  | |  | |  | |  |  | | 0 | | 1 | | 1 | | 0 | 0 | |  |  |  |
| Murnane2016 | | | | 1 | | 1 | | 1 | | 0 | 1 | |  | |  | |  | |  |  | |  | |  | |  | |  |  | | 1 | | 1 | | 1 | | 1 | 1 | | 1 | | 1 | | 1 | | 1 | u | |  |  |  |
| Murray2015 | | | | 1 | | 1 | | 1 | | u | 1 | |  | |  | |  | |  |  | | 0 | | 1 | | 0 | | 1 | 1 | |  | |  | |  | |  |  | | 1 | | 1 | | u | | 1 | 1 | |  |  |  |
| Naslund2016#1 | | | | 1 | | 1 | | 1 | | 1 | 1 | |  | |  | |  | |  |  | | 1 | | 1 | | 0 | | 1 | 1 | |  | |  | |  | |  |  | | 1 | | 1 | | 1 | | 1 | 1 | |  |  |  |
| Naslund2016#2 | | | | 1 | | 1 | | 1 | | 1 | 1 | |  | |  | |  | |  |  | |  | |  | |  | |  |  | | 1 | | 1 | | 1 | | 1 | 1 | | 1 | | 1 | | 1 | | 1 | 1 | |  |  |  |
| Naslund2019 | | | |  | |  | |  | |  |  | |  | |  | |  | |  |  | |  | |  | |  | |  |  | | 1 | | 0 | | 1 | | 1 | 1 | |  | |  | |  | |  |  | |  |  |  |
| Nicholas2017 | | | | 1 | | 1 | | 1 | | 1 | 1 | |  | |  | |  | |  |  | |  | |  | |  | |  |  | | 1 | | 0 | | 1 | | 1 | 1 | | 1 | | 1 | | 1 | | 1 | 1 | |  |  |  |
| Niendam2018 | | | |  | |  | |  | |  |  | |  | |  | |  | |  |  | | 1 | | 1 | | 0 | | 1 | 1 | |  | |  | |  | |  |  | |  | |  | |  | |  |  | |  |  |  |
| Palmier-Claus2012 | | | |  | |  | |  | |  |  | |  | |  | |  | |  |  | |  | |  | |  | |  |  | | 1 | | 1 | | 1 | | 1 | 1 | |  | |  | |  | |  |  | |  |  |  |
| Peters-Strickland2016 | | | |  | |  | |  | |  |  | |  | |  | |  | |  |  | |  | |  | |  | |  |  | | 1 | | 1 | | 1 | | 1 | 0 | |  | |  | |  | |  |  | |  |  |  |
| Poole2012 | | | | 1 | | 1 | | 1 | | 1 | 1 | |  | |  | |  | |  |  | |  | |  | |  | |  |  | |  | |  | |  | |  |  | |  | |  | |  | |  |  | |  |  |  |
| Prociow2012 | | | |  | |  | |  | |  |  | |  | |  | |  | |  |  | |  | |  | |  | |  |  | | u | | 0 | | 1 | | 0 | 1 | |  | |  | |  | |  |  | |  |  |  |
| Proudfoot2012#1 | | | |  | |  | |  | |  |  | | 1 | | 1 | | 0 | | 1 | 1 | |  | |  | |  | |  |  | |  | |  | |  | |  |  | |  | |  | |  | |  |  | |  |  |  |
| Proudfoot2012#2 | | | | 1 | | 1 | | 1 | | 1 | 1 | | 1 | | 1 | | 0 | | 1 | 0 | |  | |  | |  | |  |  | |  | |  | |  | |  |  | | 1 | | 1 | | 1 | | 1 | 1 | |  |  |  |
| Puskar2011 | | | | 1 | | 0 | | 0 | | 0 | u | |  | |  | |  | |  |  | |  | |  | |  | |  |  | |  | |  | |  | |  |  | |  | |  | |  | |  |  | |  |  |  |
| Raugh2021 | | | |  | |  | |  | |  |  | |  | |  | |  | |  |  | |  | |  | |  | |  |  | | 1 | | 1 | | 1 | | 1 | 1 | |  | |  | |  | |  |  | |  |  |  |
| Raymaker2020 | | | | 1 | | 1 | | 1 | | 1 | 1 | |  | |  | |  | |  |  | |  | |  | |  | |  |  | |  | |  | |  | |  |  | |  | |  | |  | |  |  | |  |  |  |
| Realpe2020 | | | | 1 | | 1 | | 1 | | 1 | 1 | |  | |  | |  | |  |  | |  | |  | |  | |  |  | |  | |  | |  | |  |  | |  | |  | |  | |  |  | |  |  |  |
| Roberts2017 | | | |  | |  | |  | |  |  | |  | |  | |  | |  |  | | 1 | | 1 | | 1 | | u | 1 | |  | |  | |  | |  |  | |  | |  | |  | |  |  | |  |  |  |
| Rodriguez-Villa2021 | | | | 1 | | 1 | | 1 | | 1 | 1 | |  | |  | |  | |  |  | |  | |  | |  | |  |  | |  | |  | |  | |  |  | |  | |  | |  | |  |  | |  |  |  |
| Röhricht2021 | | | | 1 | | 1 | | 1 | | 1 | 1 | | 1 | | 1 | | 1 | | 0 | 1 | |  | |  | |  | |  |  | |  | |  | |  | |  |  | | 0 | | 0 | | u | | u | 1 | |  |  |  |
| Rotondi2012 | | | |  | |  | |  | |  |  | |  | |  | |  | |  |  | | 0 | | 1 | | 1 | | 1 | 1 | |  | |  | |  | |  |  | |  | |  | |  | |  |  | |  |  |  |
| Rotondi2015 | | | |  | |  | |  | |  |  | |  | |  | |  | |  |  | | 1 | | 1 | | 1 | | 1 | 1 | |  | |  | |  | |  |  | |  | |  | |  | |  |  | |  |  |  |
| Rotondi2021 | | | |  | |  | |  | |  |  | |  | |  | |  | |  |  | | 1 | | 1 | | 1 | | 1 | 1 | |  | |  | |  | |  |  | |  | |  | |  | |  |  | |  |  |  |
| Ryan2020 | | | |  | |  | |  | |  |  | |  | |  | |  | |  |  | | 0 | | 1 | | 1 | | 1 | 1 | |  | |  | |  | |  |  | |  | |  | |  | |  |  | |  |  |  |
| Sablier2012 | | | |  | |  | |  | |  |  | |  | |  | |  | |  |  | |  | |  | |  | |  |  | | u | | u | | 1 | | 0 | 1 | |  | |  | |  | |  |  | |  |  |  |
| Saunders2017 | | | | 1 | | 1 | | 1 | | 1 | 1 | |  | |  | |  | |  |  | |  | |  | |  | |  |  | |  | |  | |  | |  |  | |  | |  | |  | |  |  | |  |  |  |
| Schlosser2016 | | | | 0 | | u | | u | | 0 | u | |  | |  | |  | |  |  | |  | |  | |  | |  |  | | u | | u | | 1 | | 0 | 0 | | 0 | | 0 | | 0 | | 0 | 0 | |  |  |  |
| Sedgwick2021 | | | | 1 | | 1 | | 1 | | 1 | 1 | |  | |  | |  | |  |  | | 1 | | 1 | | 1 | | u | 1 | |  | |  | |  | |  |  | | 1 | | 1 | | 1 | | 0 | 0 | |  |  |  |
| Simon2011 | | | |  | |  | |  | |  |  | | u | | u | | 1 | | u | 1 | |  | |  | |  | |  |  | |  | |  | |  | |  |  | |  | |  | |  | |  |  | |  |  |  |
| Sreejith2019 | | | |  | |  | |  | |  |  | |  | |  | |  | |  |  | |  | |  | |  | |  |  | | 1 | | 1 | | 1 | | 1 | 1 | |  | |  | |  | |  |  | |  |  |  |
| Steare2020#1 | | | |  | |  | |  | |  |  | | 1 | | 1 | | 1 | | 0 | 0 | |  | |  | |  | |  |  | |  | |  | |  | |  |  | |  | |  | |  | |  |  | |  |  |  |
| Steare2020#2 | | | | 1 | | 1 | | 1 | | 1 | 1 | |  | |  | |  | |  |  | |  | |  | |  | |  |  | |  | |  | |  | |  |  | |  | |  | |  | |  |  | |  |  |  |
| Storm2021 | | | | 1 | | 1 | | 1 | | 1 | 1 | |  | |  | |  | |  |  | |  | |  | |  | |  |  | | 1 | | 1 | | 1 | | 1 | 1 | | 1 | | 1 | | 1 | | 1 | 1 | |  |  |  |
| Swartz2021 | | | |  | |  | |  | |  |  | | 1 | | 1 | | 1 | | 1 | 1 | |  | |  | |  | |  |  | |  | |  | |  | |  |  | |  | |  | |  | |  |  | |  |  |  |
| Switsers2018 | | | | 1 | | 1 | | 1 | | 1 | 1 | |  | |  | |  | |  |  | |  | |  | |  | |  |  | |  | |  | |  | |  |  | |  | |  | |  | |  |  | |  |  |  |
| Terp2018 | | | | 1 | | 1 | | 1 | | 1 | 1 | |  | |  | |  | |  |  | |  | |  | |  | |  |  | |  | |  | |  | |  |  | |  | |  | |  | |  |  | |  |  |  |
| Thomas2016#1 | | | | 1 | | 1 | | 1 | | 1 | 1 | |  | |  | |  | |  |  | | 1 | | 1 | | 1 | | 1 | 1 | |  | |  | |  | |  |  | | 1 | | 1 | | 1 | | 1 | 1 | |  |  |  |
| Thomas2016#2 | | | | 1 | | 1 | | 1 | | 1 | 1 | |  | |  | |  | |  |  | |  | |  | |  | |  |  | |  | |  | |  | |  |  | |  | |  | |  | |  |  | |  |  |  |
| Thomas2017 | | | |  | |  | |  | |  |  | |  | |  | |  | |  |  | |  | |  | |  | |  |  | | 1 | | 1 | | 1 | | 1 | 1 | |  | |  | |  | |  |  | |  |  |  |
| Tho0n2022 | | | |  | |  | |  | |  |  | |  | |  | |  | |  |  | | 1 | | 1 | | 1 | | 1 | 1 | |  | |  | |  | |  |  | |  | |  | |  | |  |  | |  |  |  |
| Todd2013 | | | | 1 | | 1 | | 1 | | 1 | 1 | |  | |  | |  | |  |  | |  | |  | |  | |  |  | |  | |  | |  | |  |  | |  | |  | |  | |  |  | |  |  |  |
| Tolley2015 | | | | 1 | | 1 | | 1 | | 1 | 1 | |  | |  | |  | |  |  | |  | |  | |  | |  |  | |  | |  | |  | |  |  | |  | |  | |  | |  |  | |  |  |  |
| Torous2017 | | | |  | |  | |  | |  |  | |  | |  | |  | |  |  | |  | |  | |  | |  |  | | 1 | | 1 | | 1 | | 1 | 1 | |  | |  | |  | |  |  | |  |  |  |
| Torous2018 | | | |  | |  | |  | |  |  | |  | |  | |  | |  |  | |  | |  | |  | |  |  | | 1 | | 1 | | 1 | | 1 | 1 | |  | |  | |  | |  |  | |  |  |  |
| Vaessen2019 | | | |  | |  | |  | |  |  | |  | |  | |  | |  |  | |  | |  | |  | |  |  | | 0 | | u | | 1 | | 1 | u | |  | |  | |  | |  |  | |  |  |  |
| Vaidyam2020 | | | | 1 | | 1 | | 1 | | 1 | 1 | |  | |  | |  | |  |  | |  | |  | |  | |  |  | |  | |  | |  | |  |  | |  | |  | |  | |  |  | |  |  |  |
| Valentine2020 | | | | 1 | | 1 | | 1 | | 1 | 1 | |  | |  | |  | |  |  | |  | |  | |  | |  |  | |  | |  | |  | |  |  | |  | |  | |  | |  |  | |  |  |  |
| Välimäki2017#1 | | | |  | |  | |  | |  |  | | 1 | | 1 | | 1 | | 1 | 1 | |  | |  | |  | |  |  | |  | |  | |  | |  |  | |  | |  | |  | |  |  | |  |  |  |
| Välimäki2017#2 | | | |  | |  | |  | |  |  | | 1 | | u | | 1 | | 1 | 1 | |  | |  | |  | |  |  | |  | |  | |  | |  |  | |  | |  | |  | |  |  | |  |  |  |
| vandenHeuvel2018 | | | | 1 | | 1 | | 1 | | 1 | 1 | |  | |  | |  | |  |  | | 1 | | 1 | | 0 | | 1 | 1 | |  | |  | |  | |  |  | | 1 | | 1 | | 1 | | 1 | 1 | |  |  |  |
| vanderKrieke2012 | | | | 0 | | 0 | | u | | 0 | u | |  | |  | |  | |  |  | |  | |  | |  | |  |  | | 0 | | 0 | | 1 | | 0 | 1 | | 0 | | 0 | | u | | u | 0 | |  |  |  |
| VanTil2020 | | | |  | |  | |  | |  |  | | 1 | | 1 | | 1 | | 0 | 1 | |  | |  | |  | |  |  | |  | |  | |  | |  |  | |  | |  | |  | |  |  | |  |  |  |
| Vilardaga2016 | | | | 1 | | 1 | | 1 | | 1 | 1 | |  | |  | |  | |  |  | | 0 | | 1 | | 1 | | 0 | 1 | |  | |  | |  | |  |  | | 1 | | 1 | | 1 | | 1 | 1 | |  |  |  |
| Vilardaga2019 | | | | 1 | | 1 | | 1 | | 1 | 1 | |  | |  | |  | |  |  | | 1 | | 1 | | 1 | | u | 1 | |  | |  | |  | |  |  | | 1 | | 1 | | 0 | | u | 1 | |  |  |  |
| Wenze2016 | | | | 0 | | 0 | | u | | 1 | u | |  | |  | |  | |  |  | | 1 | | 1 | | 1 | | u | 1 | |  | |  | |  | |  |  | | 0 | | 0 | | 0 | | 0 | 0 | |  |  |  |
| Whiteman2017 | | | | 1 | | 1 | | u | | u | u | |  | |  | |  | |  |  | |  | |  | |  | |  |  | | 1 | | 0 | | 1 | | 1 | 1 | | 1 | | 1 | | 1 | | 1 | 0 | |  |  |  |
| Williams2018 | | | | 1 | | 1 | | 1 | | 1 | 1 | |  | |  | |  | |  |  | |  | |  | |  | |  |  | |  | |  | |  | |  |  | |  | |  | |  | |  |  | |  |  |  |
| Wilson2019 | | | |  | |  | |  | |  |  | |  | |  | |  | |  |  | |  | |  | |  | |  |  | |  | |  | |  | |  |  | | 1 | | 0 | | 0 | | 0 | 0 | |  |  |  |
| Xu2019#1 | | | |  | |  | |  | |  |  | | 1 | | 1 | | 0 | | 1 | 1 | |  | |  | |  | |  |  | |  | |  | |  | |  |  | |  | |  | |  | |  |  | |  |  |  |
| Xu2019#2 | | | |  | |  | |  | |  |  | |  | |  | |  | |  |  | | 1 | | 1 | | 0 | | 1 | 1 | |  | |  | |  | |  |  | |  | |  | |  | |  |  | |  |  |  |
| Ybarra2019 | | | | 1 | | 1 | | u | | 0 | u | |  | |  | |  | |  |  | |  | |  | |  | |  |  | |  | |  | |  | |  |  | |  | |  | |  | |  |  | |  |  |  |
| * u=unclear |  |  |  | |  | |  | |  | | |  | |  | |  | |  | | |  | |  | |  | |  | | |  | |  | |  | |  | | |  | |  | |  | |  | | |  | |  |  |

## Supplementary Table S5. CONSORT harms checklist

| **Study identifier: Lead author, year*** | **1- Adverse events (AEs) mentioned in the title or abstract** | **2- Information on AEs mentioned in introduction** | **3a- If article mentions use of a validated instrument to report AEs severity** | **3b- If article mentions definition of AE** | **4a- Description of how harms data were collected (e.g. diaries, phone, interviews)** | **4b- Description of when AE data were collected** | **4c- Whether or not AEs were attributed to trial interventions is reported** | **5- Description of methods for presenting and/or analysing AEs** | **6a- If article reports the number of withdrawals due to AEs in each arm** | **6b- Description of AEs leading to withdrawals** | **6c- If article report the number of deaths due to AEs in each arms** | | **7a- Provide denominators for AEs** (participants from which to analyse harms) | | **7b- Provide definitions used for analysis set (ITT, per protocol, safety data available)** | | **8a- Results presented separately for each arm** | | **8b- Separate reporting of severe adverse events (e.g. grade >2 or serious AEs)** | | **9. Describe any subgroup analyses and exploratory analyses for harms.** | | **10- Is the discussion is balanced with regards to efficacy and AEs** |
| --- | --- | --- | --- | --- | --- | --- | --- | --- | --- | --- | --- | --- | --- | --- | --- | --- | --- | --- | --- | --- | --- | --- | --- |
| Achytes2019#1 | n | n | n | n | n | n | n | n | n | n | n | | n | | n | | n | | n | | n | | n |
| Achytes2019#2 | n | n | n | n | n | n | n | n | n | n | n | | n | | n | | n | | n | | n | | n |
| Achytes2019#3 | n | n | n | n | n | n | n | n | n | n | n | | n | | n | | n | | n | | n | | n |
| Adler2020 | n | n | n | n | n | n | n | n | n | n | n | | n | | n | | n | | n | | n | | n |
| Ainsworth2013#1 | n | **yes** | n | n | n | n | n | n | n | n | n | | n | | n | | n | | n | | n | | n |
| Ainsworth2013#2 | n | **yes** | n | n | **yes** | **yes** | n | **yes** | n | n | n | | n | | n | | n/a | | n | | n | | **yes** |
| Alvarez-Jimenez2021#1 | n | n | n | n | n | n | n | n | n | n | n | | n | | n | | n | | n | | **yes** | | n |
| Alvarez-Jimenez2021#2 | n | **yes** | n | n | **yes** | n | **yes** | n | n | n | n | | n | | n | | n/a | | n | | n | | n |
| Alvarez-Jimenez2021#3 | n | n | n | n | **yes** | **yes** | n | **yes** | n | n | n | | n | | n | | n/a | | n | | n | | **yes** |
| Amiri2019 | n | n | n | n | n | n | n | n | n | n | n | | n | | n | | n | | n | | n | | n |
| Aschbrenner2016 | n | n | n | n | n | n | n | n | n | n | n | | n | | n | | n | | n | | n | | n |
| Austin2021 | n | n | n | n | **yes** | **yes** | **yes** | **yes** | **yes** | n/a | n | | **yes** | | **yes** | | n/a | | n | | n | | **yes** |
| Bain2017 | n | n | n | n | n | n | n | n | n | n | n | | n | | n | | n | | n | | n | | n |
| Baumel2016a | n | n | n | n | n | n | n | n | n | n | n | | n | | n | | n | | n | | n | | n |
| Baumel2016b | n | n | n | n | n | n | n | n | n | n | n | | n | | n | | n | | n | | n | | n |
| Bechtel2021 | n | n | n | n | n | n | n | n | n | n | n | | n | | n | | n | | n | | n | | n |
| Bell2018 | n | n | n | n | n | n | n | n | n | n | n | | n | | n | | n | | n | | n | | n |
| Bell2020 | n | n | n | n | n | n | n | n | n | n | n | | n | | **yes** | | **yes** | | n | | n | | **yes** |
| Ben-Zeev2014a#1 | n | n | n | n | n | n | n | n | n | n | n | | n | | n | | n | | n | | n | | n |
| Ben-Zeev2014a#2 | n | n | n | n | n | n | n | n | n | n | n | | n | | n | | n | | n | | n | | n |
| Ben-Zeev2014b | n | n | n | n | n | n | n | n | n | n | n | | n | | n | | n | | n | | n | | n |
| Ben-Zeev2016 | n | n | n | n | n | n | n | n | n | n | n | | n | | n | | n | | n | | n | | n |
| Ben-Zeev2018a | n | n | n | n | n | n | n | n | n | n | n | | n | | n | | n | | n | | n | | n |
| Ben-Zeev2018b#1 | n | n | n | n | n | n | n | n | n | n | n | | n | | n | | n | | n | | n | | n |
| Ben-Zeev2018b#2 | n | n | n | n | n | n | n | n | n | n | n | | n | | n | | n | | n | | n | | n |
| Ben-Zeev2018b#3 | n | n | n | n | n | n | n | n | n | n | n | | n | | n | | **yes** | | n | | n | | n |
| Ben-Zeev2018b#4 | n | n | n | n | **yes** | **yes** | n | **yes** | n | n | n | | n | | n | | n | | n | | n | | n |
| Ben-Zeev2021 | n | n | n | n | n | n | n | n | n | n | n | | n | | n | | n | | n | | n | | n |
| Berry2021 | n | n | n | n | n | n | n | n | n | n | n | | n | | n | | n | | n | | n | | n |
| Biagianti2016 | n | n | n | n | n | n | n | n | n | n | n | | n | | n | | n | | n | | n | | n |
| Bonet2020 | n | n | n | n | n | n | n | n | n | **yes** | n | | n | | **yes** | | **yes** | | n | | n | | **yes** |
| Bopp2010 | n | n | n | n | n | n | n | n | n | n | n | | n | | n | | n | | n | | n | | n |
| Bos2020 | **yes** | n | n | n | **yes** | n | n | **yes** | n | n | n | | n | | n | | n | | n | | n | | **yes** |
| Bowden2021 | n | n | n | n | n | n | n | n | n | n | n | | n | | n | | n | | n | | n | | n |
| Brunette2016 | n | n | n | n | n | n | n | n | n | n | n | | n | | n | | n | | n | | n | | n |
| Brunette2019 | n | n | n | n | n | n | n | n | n | n | n | | n | | n | | n | | n | | n | | n |
| Bucci2018a | n | **yes** | n | n | n | n | n | n | n | n | n/a | | n | | n | | n/a | | n | | n | | n |
| Bucci2019a#1 | n | n | n | n | n | n | n | n | **yes** | n/a | n/a | | n/a | | n/a | | n | | n | | n | | n |
| Bucci2019a#2 | n | n | n | n | **yes** | n | n | n | n | n | n | | n | | n | | n | | n | | n | | n |
| Camacho2021 | n | n | n | n | n | n | n | n | n | n | n | | n | | n | | n | | n | | n | | n |
| Cella2018 | n | n | n | n | n | n | n | n | n | n | n | | n | | n | | n | | n | | n | | n |
| Cella2019 | n | n | n | n | n | n | n | n | n | n | n | | n | | n | | n | | n | | n | | n |
| Chen2016 | n | n | n | n | n | n | n | n | n | n | n | | n | | n | | n | | n | | n | | n |
| Cho2020 | n | n | n | n | n | n | n | n | n | n | n | | n | | n | | n | | n | | n | | n |
| Cohen2020 | n | n | n | n | n | n | n | n | n | n | n | | n | | n | | n | | n | | n | | n |
| Dabit2021 | n | n | n | n | n | n | n | n | n | n | n | | n | | n | | n | | n | | n | | n |
| deAlmeida2018 | n | n | n | n | n | n | n | n | n | n | n | | n | | n | | n | | n | | n | | n |
| deAlmeida2019 | n | n | n | n | n | n | n | n | n | n | n | | n | | n | | n | | n | | n | | n |
| Depp2015#1 | n | n | n | n | **yes** | n | n | n | n | n | n | | n | | n | | n | | n | | n | | n |
| Depp2015#2 | n | n | n | n | n | n | n | n | n | n | n | | n | | n | | n | | n | | n | | n |
| Depp2021 | n | n | n | n | n | n | n | n | n | n | n | | n | | n | | n | | n | | n | | n |
| Eisner2019#1 | n | n | n | n | n | n | n | n | n | n | n | | n | | n | | n | | n | | n | | n |
| Eisner2019#2 | n | n | n | n | n | n | n | n | n | n | n | | n | | n | | n | | n | | n | | n |
| Enrique2020 | n | n | n | n | **yes** | **yes** | n | n | **yes** | **yes** | n | | n | | n | | n | | n | | n | | **yes** |
| Faurholt-Jepsen2019 | n | n | n | n | n | n | n | n | n | n | n | | n | | n | | n | | n | | n | | n |
| Faurholt-Jepsen2020 | n | n | n | n | n | n | n | n | n | n | n | | n | | n | | n | | n | | n | | n |
| Fellendorf2021 | n | n | n | n | n | n | n | n | n | n | n | | n | | n | | n | | n | | n | | **yes** |
| Ferreira-Correia2018 | n | n | n | n | n | n | n | n | n | n | n | | n | | n | | n | | n | | n | | n |
| Ferron2012 | n | n | n | n | n | n | n | n | n | n | n | | n | | n | | n | | n | | n | | n |
| Forchuk2015#1 | n | n | n | n | n | n | n | n | n | n | n | | n | | n | | n | | n | | n | | **yes** |
| Forchuk2015#2 | n | n | n | n | n | n | n | n | n | n | n | | n | | n | | n | | n | | n | | n |
| Fowler2021 | **yes** | **yes** | **yes** | N | n | n | **yes** | **yes** | n/a | **yes** | **yes** | | **yes** | | **yes** | | n/a | | **yes** | | n | | n |
| Fulford2020 | n | n | n | n | n | n | n | n | n | n | n | | n | | n | | n | | n | | n | | n |
| Garety2021 | n | n | n | **yes** | n | **yes** | **yes** | n | **yes** | **yes** | **yes** | | **yes** | | **yes** | | **yes** | | **yes** | | n | | **yes** |
| Golas2015 | n | n | n | n | n | n | n | n | n | n | n | | n | | n | | n | | n | | n | | n |
| Gorczynski2014 | n | n | n | n | n | n | n | n | n | n | n | | n | | n | | n | | n | | n | | n |
| Gordon-Smith2019 | n | n | n | n | n | n | n | n | n | n | n | | n | | n | | n | | n | | n | | n |
| Granholm2012 | n | n | n | n | n | n | n | n | n | n | n | | n | | n | | n | | n | | n | | n |
| Hanssen2020#1 | n | n | n | n | n | n | n | n | n | **yes** | n/a | | n/a | | n/a | | n | | n | | n | | n |
| Hanssen2020#2 | n | **yes** | n | n | n | n | n | n | n | n | n | | n | | n | | n | | n | | n | | n |
| Hanssen2020#3 | n | n | n | n | n | n | n | n | n | n | n | | n | | n | | n | | n | | n | | n |
| Hanssen2020#4 | n | n | n | n | n | **yes** | n | **yes** | n | n | n | | n | | n | | n | | n | | n | | **yes** |
| Hardy2018 | n | n | n | n | **yes** | n | n | n | **yes** | n | n | | n | | n | | n | | n | | n | | n |
| Harris2017 | n | n | n | n | n | n | n | n | n | n | n | | n | | n | | n | | n | | n | | n |
| Henson2020#1 | n | n | n | n | n | n | n | n | n | n | n | | n | | n | | n | | n | | n | | n |
| Henson2020#2 | n | n | n | n | n | n | n | n | n | n | n | n | | n | | n | | n | | n | | n | |
| Hidalgo-Mazzei2016#1 | n | **yes** | n | n | **yes** | **yes** | n | n | n | n | n | n | | n | | n | | n | | n | | n | |
| Hidalgo-Mazzei2016#2 | n | n | n | n | n | n | n | n | n | n | n | n | | n | | n | | n | | n | | n | |
| Hidalgo-Mazzei2016#3 | n | n | n | n | n | n | n | n | n | n | n | n | | n | | n | | n | | n | | n | |
| Hidalgo-Mazzei2018 | n | **yes** | n | n | **yes** | **yes** | n | n | n | n | n | **yes** | | **yes** | | **yes** | | n | | n | | n | |
| Jonathan2021 | n | n | n | n | n | n | n | n | n | n | n | n | | n | | n | | n | | n | | n | |
| Kane2013 | **yes** | n | n | n | **yes** | **yes** | **yes** | **yes** | n | **yes** | n | **yes** | | **yes** | | n/a | | n | | n | | **yes** | |
| Kidd2019 | n | y | n | n | **yes** | **yes** | **yes** | n | n | **yes** | n | n | | n | | n/a | | n | | n | | **yes** | |
| Kim2016 | n | n | n | n | n | n | n | n | n | n | n | n | | n | | n | | n | | n | | n | |
| Kopelowicz2017 | n | n | n | n | **yes** | **yes** | **yes** | **yes** | **yes** | **yes** | n | **yes** | | **yes** | | **yes** | | **yes** | | n | | **yes** | |
| Kreyenbuhl2019 | n | n | n | n | n | n | n | n | n | n | n | n | | n | | n | | n | | n | | n | |
| Krzystanek2019 | **yes** | n | n | n | **yes** | **yes** | **yes** | **yes** | n | n | **yes** | **yes** | | n | | **yes** | | **yes** | | n | | **yes** | |
| Lahti2021 | n | n | n | n | n | **yes** | n | n | n | n | n | n | | n | | n | | n | | n | | **yes** | |
| Laine2019 | n | n | n | n | n | n | n | n | n | n | n | n | | n | | n | | n | | n | | n | |
| Lederman2014 | n | n | n | n | n | n | n | n | n | n | n | n | | n | | n | | n | | n | | n | |
| Lewandowski2021 | n | n | n | n | **yes** | **yes** | n | n | n | n | n | n | | n | | n | | n | | n | | n | |
| Lewis2020 | n | n | n | n | **yes** | **yes** | n | n | n | n | n | **yes** | | n | | n | | n | | n | | n | |
| Lim2020 | n | n | n | n | n | n | n | n | n/a | n/a | **yes** | n/a | | n/a | | n/a | | n | | n/a | | n | |
| Lobban2017#1 | **yes** | **yes** | n | n | **yes** | n | **yes** | **yes** | n | n | **yes** | **yes** | | **yes** | | **yes** | | n | | n | | **yes** | |
| Lobban2017#2 | n | n | n | n | **yes** | n | n | **yes** | n | n | n | n | | n | | n | | n | | n | | n | |
| Lopez-Morinigo2021 | n | n | n | n | n | n | n | n | n | n | n | n | | n | | n | | n | | n | | n | |
| Ludwig2021 | n | n | n | n | n | n | n | n | n | n | n | n | | n | | n | | n | | n | | n | |
| Luther2020 | n | n | n | n | n | n | n | n | n | n | n | n | | n | | n | | n | | n | | n | |
| McEnery2021 | **yes** | n | n | **yes** | **yes** | **yes** | **yes** | n | n | n | n | n | | n | | n | | n | | n | | **yes** | |
| McKnight2017 | n | n | n | n | n | n | n | n | n | n | n | n | | n | | n | | n | | n | | n | |
| Medalia2021 | n | n | n | n | n | n | n | n | n | n | n | n | | n | | n | | n | | n | | n | |
| Medenblik2020 | n | n | n | n | n | n | n | n | n | n | n | n | | n | | n | | n | | n | | n | |
| Merzenich2014 | n | n | n | n | n | n | n | n | **yes** | **yes** | **yes** | n | | n | | n/a | | n | | n | | n | |
| Meyer2018 | n | n | n | n | n | n | n | n | n | n | n | n | | n | | n | | n | | n | | n | |
| Michalak2019#1 | n | n | n | n | **yes** | n | n | **yes** | n | n | n | n | | n | | n | | n | | n | | **yes** | |
| Michalak2019#2 | **yes** | **yes** | n | n | **yes** | **yes** | n | **yes** | n | n | n | | n | | n | | n | | n | | n | | **yes** |
| Minor2021#1 | n | n | n | n | n | n | n | n | n | n | n | | n | | n | | n | | n | | n | | n |
| Minor2021#2 | n | n | n | n | n | n | n | n | n | n | n | | n | | n | | n | | n | | n | | n |
| Mistler2017 | **yes** | n | n | n | **yes** | n | n | n | n | n | n | | n | | n | | n | | n | | n | | n |
| Moitra2017 | n | n | n | n | **yes** | **yes** | n | n | n | n | n | | n | | n | | n | | n | | n | | n |
| Moitra2021 | n | n | n | n | n | n | n | n | n | n | n | | n/a | | n/a | | n/a | | n | | n | | n |
| Montes2012 | n | n | n | n | n | n | n | n | n | n | n | | n | | n | | n | | n | | n | | n |
| Moritz2015 | n | n | n | n | n | n | n | n | n | n | n | | n | | n | | n | | n | | n | | n |
| Moritz2016 | n | n | n | n | n | n | n | n | n | n | n | | n | | n | | n | | n | | n | | n |
| Moura2019 | n | n | n | n | n | n | n | n | n | n | n | | n | | n | | n | | n | | n | | n |
| Mueller2018 | n | n | n | n | n | n | n | n | **yes** | **yes** | n | | n | | n | | n/a | | n | | n | | n |
| Murray2015 | n | **yes** | n | n | **yes** | **yes** | **yes** | **yes** | n | n | n | | n | | n | | n | | n | | n | | **yes** |
| Naslund2016#1 | n | n | n | n | n | n | n | n | n | n | n | | n | | n | | n | | n | | n | | n |
| Naslund2016#2 | n | n | n | n | **yes** | **yes** | n | n | n | **yes** | n | | n | | n | | n | | n | | n | | **yes** |
| Niendam2018 | n | n | n | n | n | n | n | n | n | n | n | | n | | n | | n | | n | | n | | n |
| Palmier-Claus2012 | n | n | n | n | n | n | n | **yes** | n | n | n | | n | | n | | n/a | | n | | **yes** | | **yes** |
| Peters-Strickland2016 | n | **yes** | **yes** | **yes** | **yes** | n | **yes** | **yes** | n/a | **yes** | N | | **yes** | | **yes** | | n/a | | **yes** | | N | | **yes** |
| Poole2012 | n | n | n | n | n | n | n | **yes** | n | n | n | | n | | n | | n | | n | | n | | n |
| Prociow2012 | n | n | n | n | n | n | n | n | n | n | n | | n | | n | | n | | n | | n | | n |
| Proudfoot2012#1 | n | n | n | n | n | n | n | n | n | n | n | | n | | n | | n | | n | | n | | n |
| Proudfoot2012#2 | n | **yes** | n | n | **yes** | **yes** | **yes** | **yes** | n | n | n | | n | | n | | n | | n | | n | | n |
| Puskar2011 | n | n | n | n | n | n | n | n | n | n | n | | n | | n | | n | | n | | n | | **yes** |
| Raugh2021 | n | n | n | n | **yes** | **yes** | n | n | n | n | n | | n | | n | | n/a | | n | | n | | **yes** |
| Roberts2017 | n | n | n | n | n | n | n | n | n | n | n | | n | | n | | n | | n | | n | | n |
| Röhricht2021 | n | n | n | n | n | n | n | n | n | n | n | | n | | n | | n | | n | | n | | n |
| Rotondi2010 | n | n | n | n | n | n | n | n | n | n | n | | n | | n | | n | | n | | n | | n |
| Ryan2020 | n | n | n | n | n | n | n | n | n | n | n | | n | | n | | n | | n | | n | | n |
| Sablier2012 | n | n | n | n | n | n | n | n | n | n | n | | n | | n | | n | | n | | n | | n |
| Saunders2017 | n | n | n | n | **yes** | n | n | **yes** | n | n | n | | n | | n | | n | | n | | n | | n |
| Schlosser2016 | n | n | n | n | n | n | n | n | n | n | n | | n | | n | | n | | n | | n | | n |
| Sedgwick2021 | n | n | n | n | **yes** | **yes** | n | n | n | n | n | | n | | n | | n | | n | | n | | n |
| Shin2016 | n | n | n | n | n | n | n | n | n | n | n | | n | | n | | n | | n | | n | | n |
| Sibeko2017 | n | n | n | n | n | n | n | n | n | n | n | | n | | n | | n | | n | | n | | n |
| Simon2011 | n | n | n | n | n | n | n | n | n | n | n | | n | | n | | n | | n | | n | | n |
| Spaniel2012 | n | n | n | n | n | n | n | n | n | n | n | | n | | n | | n | | n | | n | | n |
| Spaniel2015 | n | n | n | n | n | n | n | n | n | n | n | | n | | n | | n | | n | | n | | n |
| Steare2020#1 | n | n | n | n | n | n | **yes** | n | n | n/a | n | | n | | n | | n | | n | | n | | **yes** |
| Steare2020#2 | n | n | n | n | **yes** | n | n | n | n | n | n | | n | | n | | n | | n | | n | | n |
| Swartz2021 | n | n | n | n | n | n | n | n | n | n | n | | n | | n | | n | | n | | n | | n |
| Terp2018 | n | **yes** | n | n | **yes** | **yes** | n | **yes** | n | n | n | | **yes** | | **yes** | | n/a | | n | | n | | **yes** |
| Tessier2020 | No | No | n | n | n | n | n | n | n | n | n | | n | | n | | n | | n | | n | | n |
| Thomas2016#1 | n | n | n | n | n | n | n | n | n | n | n | | n | | n | | n | | n | | n | | n |
| Thomas2016#2 | n | n | n | n | n | n | n | n | n | n | n | | n | | n | | n/a | | n | | n | | n |
| Thonon2022 | n | n | n | n | n | n | n | n | **yes** | **yes** | n | | **yes** | | **yes** | | n/a | | n | | n | | **yes** |
| Torous2017 | n | n | n | n | n | n | n | n | n | n | n | | n | | n | | n | | n | | n | | n |
| Vaessen2019 | n | n | n | n | n | n | n | n | n | n | n | | n | | n | | n | | n | | n | | n |
| Vaidyam2020 | n | n | n | n | n | n | n | n | n | n | n | | n | | n | | n | | n | | n | | **yes** |
| Välimäki2017#1 | **yes** | **yes** | **yes** | **yes** | **yes** | **yes** | **yes** | **yes** | n | n | **yes** | | **yes** | | **yes** | | **yes** | | **yes** | | **yes** | | **yes** |
| Välimäki2017#2 | n | n | n | n | n | n | n | n | n | n | n | | n | | n | | n | | n | | n | | n |
| vandenHeuvel2018 | n | n | n | n | n | n | n | n | n | n | n | | n | | n | | n | | n | | n | | n |
| vanderKrieke2013 | n | n | n | n | n | n | n | n | n | n | n | | n | | n | | n | | n | | n | | n |
| VanTil2020 | n | n | n | n | n | n | n | n | n | n | n | | n | | n | | n | | n | | n | | n |
| Vázquez-Campo2016 | n | n | n | n | n | n | n | n | n | n | n | | n | | n | | n | | n | | n | | n |
| Vilardaga2016 | n | n | n | n | y | n | n | y | n | n | n | | n | | n | | n | | n | | n | | n |
| Vilardaga2019 | **yes** | **yes** | n | n | n | **yes** | **yes** | **yes** | **yes** | **yes** | n | | n | | n | | n | | n | | n | | n |
| Wenze2016 | n | n | n | n | n | **yes** | n | n | n | n | n | | n | | n | | n | | n | | n | | n |
| Wilson2019 | n | n | n | n | n | n | n | n | **yes** | n | n | | n | | n | | n/a | | n | | n | | n |
| Xu2019#1 | **yes** | n | n | n | **yes** | **yes** | n | **yes** | n | n/a | **yes** | | **yes** | | **yes** | | **yes** | | n | | n | | **yes** |
| Xu2019#2 | n | n | n | n | **yes** | n | n | n | n | n | n | | n | | **yes** | | **yes** | | n | | n | | n |
| Xu2020 | n | n | n | n | n | n | n | n | n | n | n | | n | | n | | n | | n | | n | | n |
| Zhu2020 | n | n | n | n | n | n | n | n | n | n | n | | n | | n | | n | | n | | n | | n |
| **Total studies where ‘yes’** | 11 | 16 | 3 | 4 | 37 | 29 | 16 | 24 | 11 | 14 | 8 | | 14 | | 15 | | 11 | | 6 | | 3 | | 32 |

## Supplementary Table S6. Quantitative associations between demographic variables and digital health intervention engagement in reviewed studies

**Note:** numbers preceded by “#” are study codes used to identify studies throughout this multimedia appendix. See Table S8 for full reference details and for the corresponding citation number in main manuscript reference list (where applicable). Some studies may appear in both significant and non-significant columns if more than one user engagement index was used.

| **Variable** | **Papers with no significant effect on engagement** | **Papers with significant effect on engagement** | **Details of significant effects** |
| --- | --- | --- | --- |
| Age | #008, #017, #024, #050, #077, #089, #122, #130, #169, #182, #221, #225, #236, #243, #262, #265, #291, #317, #335, #338, #341, #342, #355, #370, #384, #398, #445, #455 | #023, #055, #083, #156, #176, #188, #245, #265, #362, #381, #478 | Higher age, higher engagement   - #023: Higher use in people 35 and older compared to those younger than 35 (IRR 1.9, p < 0.01 for 35–45 years and IRR 2.4, p < 0.001 for 45 years or older). - #055: On average the activity rate increased by 2% for each year of age (IRR = 1.02) - #188: When controlling for potential predictors, older age [and other variables] predicted spending more time in the motivation section of the program and the decision support section. - #245: older age significantly increased the odds of retention (OR = 1.021, Coefficient = 0.021, SE = 0.00613, z = 3.47, p < 0.001, CI 95% = 0.0092–0.033). - #362: Participants over 30 years of age were estimated to complete an average of 1.04 more workbooks than those under 30 years of age - #381: participants over 30 years completed an average of 1.09 more workbooks than their younger counterparts   Younger age, higher engagement   - #156: the older the individuals, more difficulties in using these mobile applications [r(100) = .209, p < 0.05]. - #176: Univariate models: users were younger than nonusers (42.33 [SD 10.75] vs 47.69 [SD 9.76] years, respectively; t75=–3.46; P=.001). Multivariate model: Age (OR 0.93, 95% CI 0.86-0.99; P=.048) … remained significant. - #265: Patients who refused to participate were older than consenting, randomized patients (P=.001) - #478: Adherence was not significantly influenced by age or sex at either study start or study end (P>0.3), but there was a significant interaction between method, time, and age whereby Fitbit adherence decreased a significant 23.4% for participants at 50 years of age (P<0.001, F[1,3916]=24.1) compared to 10.2% for participants at 30 years of age (P=0.06, F[1,3916]=3.4). Further, app adherence decreased a nonsignificant 5.5% for participants at 50 years of age (P=0.25, F[1,3916]=1.3) and a similar 7.0% for participants at 30 years of age (P=0.21, F[1,3916]=1.6).   Both (for different engagement indices)   - #083: Older participants (46-60) were significantly more engaged in Days of On-Demand Use (0.48 days more weekly) and Daily On-Demand Use (1.78 uses more per day) when compared to those 18-29. However, they were significantly less engaged in Days Responding to Prompts (0.41 days fewer). |
| Gender or sex | #050, #055, #077, #089, #122, #176, #182, #221, #236, #291, #317, #329, #338, #342, #355, #370, #398, #445, | #023, #083, #265, #341, #362, #381, #472 | Higher engagement among females   - #023: Significantly higher rates of use of FOCUS modules among females compared to males (IRR 1.6, p < 0.05 for self-initiated off-hours use, and total use, IRR 1.4, p < 0.05) - #083: Females were significantly more engaged as measured by Days of mHealth Use, Days Responding to Prompts, and Daily On-Demand Use. On average, females used FOCUS on 0.42 days more per week than males and responded to prompts on 0.18 days more than males. On average, females also used on-demand features 1.61 times more per day than males. - #341: In univariate analysis, being a female was significantly associated with completing more random and end-of-day EMA samples (r = .29, p = .031; r = .35, p = .009, respectively). However, this did not remain significant in multivariate analysis. - #362: Male gender was a significant predictor of attrition; males were estimated to complete an average of 0.98 fewer workbooks than females, holding all other variables constant. - #381: females completed an average of 1.27 more workbooks   Higher engagement among males   - #265: intervention dropouts were more often women than men (χ 2 1=7.2, P=.009)   More perceived harm among women   - #472: Among patient participants, more women than men perceived harm from the SMS intervention (35/223, 15.7% vs 16/178, 9.0%, P=.05). More female than male nurses thought that the SMS messages caused harm to patients (8/41, 19.5% vs 0/17, 0%, P=.05). |
| Education | #024, #077, #089, #130, #169, #225, #236, #262, #265, #317, #341, #342, #362, #384, #398, #445 | #023, #055, #176,  #384 | Higher education level, higher engagement   - #023: Significantly higher rates of use of the different FOCUS modules in patients with higher levels of education compared to those without a high school diploma (IRR 1.7, p < 0.05 for any self-initiated off-hours FOCUS use), - #055: Mann-Whitney U tests demonstrated that depth of use was significantly greater amongst individuals with a tertiary education than those without (U = 853.0, P = .036) but breadth of use was not associated with education (U = 896, P = .076). - #176: In univariate analysis, the proportion of less educated people agreeing to use the DHI was lower than that among nonusers (1/24, 8% vs 8/53, 23%, respectively; χ21=4.0; P=.045), but this effect was not significant in multivariate analysis. - #384: higher education predicted higher wearable adherence |
| Race, ethnicity, or country of origin | #023, #089, #122, #169, #317, #338, #341, #342, #398 | #083, #102 | - #083: White participants had significantly more Days of mHealth Use (0.69 more per week), Days Responding to Prompts (0.72 more per week), and Days of On-Demand Use (0.17 more per week) than African-American participants. White participants had significantly more Days Responding to Prompts (0.74 more per week) and Days of On-Demand Use (0.33 more per week) but less Daily On-Demand Use (1.32 less uses per day) than Hispanic participants. - #102: A higher proportion of immigrant participants (10/11, 90.9%) than native Spanish participants (48/78, 61.5%) agreed to use the ReMindCare app (χ2=4.2, P=.04). |
| IQ | #176 | #024, #225 | Higher IQ, higher engagement   - #024: Bivariate correlations showed that full study adherence was positively related to IQ (r=0.45; P=.008), with those with higher IQ completing more app question prompts. - #225: Noncompleters had lower estimated premorbid verbal IQ (ANART) than completers. |
| Reading level | #080 | -- | -- |
| Employ-ment | #089, #265, #342, #355, #384 | -- | -- |
| Marital status | #077, #265 | -- | -- |

## Supplementary Table S7. Associations between clinical variables and digital health intervention engagement in reviewed studies

**Note:** numbers preceded by “#” are study codes used to identify studies throughout this multimedia appendix. See Table S8 for full reference details and for the corresponding citation number in main manuscript reference list (where applicable). Some studies may appear in both significant and non-significant columns if more than one user engagement index was used.

| **Variable** | **Papers with no significant effect on engagement** | **Papers with significant effect on engagement** | **Details of significant effects** | **Papers with qualitative feedback** |
| --- | --- | --- | --- | --- |
| Mood symptoms | #130, #225, #362, #364, #370, #381, #489 | #122, #169, #271, #315 | Higher depression, higher engagement   - #271: A4i users were at baseline more depressed (x̄ = 8.33 (SD = 5.81) vs 4.80 (SD = 4.95); (t = 2.02, df = 36, p = .05)   Higher depression, lower engagement   - #169: Percentage app completion was significantly and inversely correlated with baseline depression.   Other significant effects related to mood   - #122: significant interactions in the models involving depressive symptom severity (B = 0.77, SE = 0.37, P = .04) such that higher depression corresponded with reduced use in the non-DHI condition, but a relative sustainment of engagement in the DHI condition. - #315: At baseline, active participants endorsed higher positive (d = 0.48) and negative affect (d = 0.36) than inactive participants. | #162, #168, #350, #375, #377 |
| Positive symptoms | #062, #176, #225, #262, #341, #364, #370, #384 | #335, #370, #474 | Higher positive symptoms, lower engagement   - #335: Spearman correlation demonstrated a significant negative relationship between PANSS positive score and sleep diary completion (ρ=−.49, P<.05) and symptom diary completion (ρ=−.40, P<.01). - #370: Positive symptom subscale severity was the only significant predictor of compliance with the methodology (OR = 0.68, p = .033, CI: 0.48 – 0.97). - #474: Remaining in the study was significantly associated with fewer past psychotic events (τ = −0.297, p = 0.004) | #035, #047, #057, #066, #083, #086, #093, #117, #154, #168, #375, #404, #412, #430, #472 |
| Negative symptoms | #080, #262, #317, #341, #364, #370, #384, | #102, #225, #335, #370, | Higher negative symptoms, lower engagement   - #102: Participants choosing not to use the DHI showed higher scores on the PANSS N5 and N6 negative subscales (χ2=12.8, P=.03; χ2=12.9, P=.02, respectively). - #225: Non-completers had more severe negative symptoms (PANSS negative) than completers. - #335: PANSS negative score was negatively correlated with wearable adherence (ρ=−.49, P<.05), sleep diary completion (ρ=−.75, P=.001), and symptom diary completion (ρ=−.53, P<.05).   Negative symptoms predict reactivity to the method   - #370: Regression analysis showed that negative symptoms (PANSS) significantly predicted reactivity to answering symptom questions on a DHI (β =.54, p = .001). | #056, #093, #095, #160, #375, #443 |
| Cognitive difficulties | #080, #104, #262, #341, #384 | #176, #188 | - #176: Those agreeing to use a DHI had better executive function performance, self-reflectiveness and metacognitive performance than non-users, but none of the associations were significant in multivariate analysis. - #188: Greater cognitive impairment predicted spending more time in the motivation section of the program. | #062, #064, #068, #330, #351, #377, #450, #491 |
| General symptoms, previous hospital admission, or past suicide attempts | #077, #102, #169, #364, #407 | #083, #342 | Higher symptoms, higher engagement   - #342: baseline BPRS score was significantly correlated with DHI completion rates, such that increased psychiatric severity was associated with increased completion rates (r=.73, p=.025). - #474: Remaining in the study was significantly associated a higher number of past suicide attempts (τ= 0.306, p = 0.001)   More previous hospital admissions, lower engagement   - #083: Greater number of psychiatric hospitalizations was also significantly associated with likelihood of discontinuing use, with a discontinuation hazard ratio of 1.4 (95% CI 1.1-1.8; P=.004) for 7+ hospitalizations compared to fewer hospitalizations. | #238, #340, #375, #412, #443 |
| Functioning | #355, #407 | #225, #243, #384 | Higher functioning, higher engagement   - #225: Non-completers had lower self-reported living skills than completers. - #384: Higher functioning predicted greater event survey adherence   Higher functioning, lower engagement   - #243: Worse functioning (higher total Functional Assessment Short Test score) was weakly but significantly associated with continued ap use (b=1.12, p=0.02) - #384: Higher functioning predicted lower VOX data | #047, #238 |
| Diagnosis | #027, #077, #089, #169, #176, #236, #265, #284, #329, #455 | #122, #188, #211, #474 | - #122: Among those with bipolar disorder, engagement with DHI was significantly higher than with a non-DHI (4.27 more weeks engaged in FOCUS, 95% CI [1.61, 6.96], d = .97, P = .002) whereas the difference was not significant for those with schizophrenia/schizoaffective disorder or major depressive disorder. - #188: People with a diagnosis of schizophrenia or schizoaffective disorder spent significantly more time in the motivation and decision support sections of the program. - #221: There was a significantly higher proportion of individuals with a DSM-IV diagnosis of bipolar II disorder who registered compared to individuals who did not register (37% vs 29% respectively, p < 0.001). - #474: Remaining in the study was significantly associated with having rapid cycling bipolar (τ = 0.28, p = 0.009) | #094 |

## Supplementary Table S8. Reference list of studies included in the main review.

**Note:** Numbers in the first column correspond to the reference number in the main manuscript reference list (n/a indicates “not applicable”); numbers in the second column (“#”) are study codes used to identify studies throughout this multimedia appendix.

| **Number in main manuscript reference list** | **#** | **Study identifier: Primary paper lead author, year, #, paper number (if >1 paper per study)** | **Study details** |
| --- | --- | --- | --- |
| 46 | 4 | Chiauzzi2019 | Chiauzzi E, Newell A. Mental Health Apps in Psychiatric Treatment: A Patient Perspective on Real World Technology Usage. JMIR MENTAL HEALTH. 2019;6(4). |
| n/a | 8 | Bonet2018 | Bonet L, Llacer B, Hernandez-Viadel M, Arce D, Blanquer I, Canete C, et al. Differences in the Use and Opinions About New eHealth Technologies Among Patients With Psychosis: Structured Questionnaire. JMIR MENTAL HEALTH. 2018;5(3). |
| 107 | 11 | Bucci2019a#2 | Berry N (2020) Developing a Theory-Informed Smartphone App for Early Psychosis: Learning Points From a Multidisciplinary Collaboration., Edge D, Lewis S, Morris R, Machin M, Ainsworth J, et al. Frontiers in Psychiatry. 2020;11:602861. |
| n/a | 17 | Naslund2019 | Naslund JA, Aschbrenner KA, McHugo GJ, Marsch LA, Unutzer J, Bartels SJ. Exploring opportunities to support mental health care using social media: A survey of social media users with mental illness. Early Intervention in Psychiatry. 2019;13(3):405-13. |
| 100 | 19 | Brunette2016 | Brunette MF (2016) Development and usability testing of a web-based smoking cessation treatment for smokers with schizophrenia, Ferron JC, Gottlieb J, Devitt T, Rotondi A.. Internet Interventions.;4:113-9. |
| 50 | 20 | Michalak2019#1 | Michalak EE, Morton E, Barnes SJ, Hole R, Murray G. Supporting Self-Management in Bipolar Disorder: Mixed-Methods Knowledge Translation Study. JMIR MENTAL HEALTH. 2019;6(4). |
| 123 | 22 | Vilardaga2016 | Vilardaga, R., et al. (2016). User experience evaluation of a smoking cessation app in people With serious mental illness. Nicotine and Tobacco Research 18(5): 1032-1038. |
| 75 | 23 | Achytes2019#1 | Achytes et al. (2019). Off-hours use of a smartphone intervention to extend support for individuals with schizophrenia spectrum disorders recently discharged from a psychiatric hospital. Schizophrenia Research 206: 200-208. |
| 38 | 24 | Ryan2020 | Ryan, K. A., et al. (2020). A smartphone app to monitor mood symptoms in bipolar disorder: Development and usability study. JMIR Mental Health 7(9): e19476. |
| 71 | 26 | Bucci2018a | Bucci, S., et al. (2018). Actissist: Proof-of-concept trial of a theory-driven digital intervention for psychosis. Schizophrenia Bulletin 44(5): 1070-1080. |
| n/a | 27 | Fowler2021 | Fowler, J. C., et al. (2021). Hummingbird study: Results from an exploratory trial assessing the performance and acceptance of a digital medicine system in adults with schizophrenia, schizoaffective disorder, or first-episode psychosis. Neuropsychiatric Disease and Treatment 17: 483-492. |
| 132 | 28 | Ferreira-Correia2018 | Ferreira-Correia, A., et al. (2018). Barriers to the implementation of a computer-based rehabilitation programme in two public psychiatric settings. South African Journal of Psychiatry 24(1): a1163. |
| 42 | 29 | Lobban2017#1 | Lobban, F., et al. (2017). Assessing Feasibility and Acceptability of Web-Based Enhanced Relapse Prevention for Bipolar Disorder (ERPonline): A Randomized Controlled Trial. J Med Internet Res 19(3): e85. |
| 54 | 35 | Alvarez-Jimenez2021#2 | Valentine, L., et al. (2020). Young people's experience of a long-term social media-based intervention for first-episode psychosis: Qualitative analysis. Journal of Medical Internet Research 22(6): e17570. |
| 102 | 45 | Ainsworth2013#1 | Ainsworth, J., et al. (2013). A comparison of two delivery modalities of a mobile phone-based assessment for serious mental illness: native smartphone application vs text-messaging only implementations. J Med Internet Res 15(4): e60. |
| 31 | 47 | Allan2019 | Allan, S., et al. (2019). Developing a hypothetical implementation framework of expectations for monitoring early signs of psychosis relapse using a mobile app: Qualitative study. Journal of Medical Internet Research 21(10). |
| n/a | 50 | Alvarez-Jimenez2021#1 | Alvarez-Jimenez, M., et al. (2021). The Horyzons project: a randomized controlled trial of a novel online social therapy to maintain treatment effects from specialist first-episode psychosis services. World Psychiatry 20(2): 233-243. |
| 133 | 55 | Hanssen2020#3 | Arnold, C., et al. (2019). Predicting engagement with an online psychosocial intervention for psychosis: Exploring individual- and intervention-level predictors. Internet Interventions 18: 100266. |
| 51 | 56 | Hanssen2020#4 | Arnold, C., et al. (2020). Engaging with a web-based psychosocial intervention for psychosis: Qualitative study of user experiences. JMIR Mental Health 7(6): e16730. |
| 56 | 57 | Aschbrenner2016 | Aschbrenner, K. A., et al. (2016). A mixed methods study of peer-to-peer support in a group-based lifestyle intervention for adults with serious mental illness. Psychiatric rehabilitation journal 39(4): 328-334. |
| 86 | 62 | Austin2021 | Austin, S. F., et al. (2021). Service User Experiences of Integrating a Mobile Solution (IMPACHS) Into Clinical Treatment for Psychosis. Qualitative health research 31(5): 942-954. |
| 147 | 64 | Barnes2011 | Barnes, E., et al. (2011). Developing an online psychoeducation package for bipolar disorder. Journal of Mental Health 20(1): 21-31. |
| 85 | 66 | Ainsworth2013#2 | Palmier-Claus, et al. (2013). Integrating mobile-phone based assessment for psychosis into people's everyday lives and clinical care: A qualitative study. BMC Psychiatry 13: 34. |
| 32 | 68 | Baumel2016a | Baumel, A., et al. (2016). Adaptation of a peer based online emotional support program as an adjunct to treatment for people with schizophrenia-spectrum disorders. Internet Interventions 4: 35-42. |
| n/a | 69 | Baumel2016b | Baumel, A., et al. (2016). Health technology intervention after hospitalization for schizophrenia: Service utilization and user satisfaction. Psychiatric Services 67(9): 1035-1038. |
| 76 | 71 | Bechtel2021 | Bechtel, J. M., et al. (2021). Care manager perspectives on integrating an mHealth app system into clinical workflows: A mixed methods study. General Hospital Psychiatry 68: 38-45. |
| 95 | 76 | Bell2018 | Bell, I. H., et al. (2018). Smartphone-based ecological momentary assessment and intervention in a blended coping-focused therapy for distressing voices: Development and case illustration. INTERNET INTERVENTIONS-THE APPLICATION OF INFORMATION TECHNOLOGY IN MENTAL AND BEHAVIOURAL HEALTH 14: 18-25. |
| 96 | 77 | Bell2020 | Bell, I. H., et al. (2020). Pilot randomised controlled trial of a brief coping-focused intervention for hearing voices blended with smartphone-based ecological momentary assessment and intervention (SAVVy): Feasibility, acceptability and preliminary clinical outcomes. Schizophrenia Research 216: 479-487. |
| 151 | 78 | Ben-Zeev2013 | Ben-Zeev, D., et al. (2013). Development and usability testing of FOCUS: A smartphone system for self-management of schizophrenia. Special Issue: Illness/Wellness Management for Individuals With Serious Mental Illnesses 36(4): 289-296. |
| 140 | 80 | Ben-Zeev2014b | Ben-Zeev, D., et al. (2014). Feasibility, acceptability, and preliminary efficacy of a smartphone intervention for schizophrenia. Schizophrenia Bulletin 40(6): 1244-1253. |
| 36 | 83 | Achytes2019#2 | Ben-Zeev, D., et al. (2016). mHealth for Schizophrenia: Patient Engagement With a Mobile Phone Intervention Following Hospital Discharge. JMIR Mental Health 3(3). |
| 154 | 84 | Ben-Zeev2016 | Ben-Zeev, D., et al. (2016). Mobile Behavioral Sensing for Outpatients and Inpatients With Schizophrenia. Psychiatr Serv 67(5): 558-561. |
| n/a | 85 | Ben-Zeev2018b#1 | Ben-Zeev, D., et al. (2018). Mobile Health (mHealth) Versus Clinic-Based Group Intervention for People With Serious Mental Illness: A Randomized Controlled Trial. Psychiatr Serv 69(9): 978-985. |
| 43 | 86 | Ben-Zeev2018a | Ben-Zeev, D., et al. (2018). Video-based mobile health interventions for people with schizophrenia: Bringing the pocket therapist to life. Psychiatric rehabilitation journal 41(1): 39-45. |
| n/a | 89 | Ben-Zeev2021 | Ben-Zeev, D., et al. (2021). A Smartphone Intervention for People With Serious Mental Illness: Fully Remote Randomized Controlled Trial of CORE. J Med Internet Res 23(11): e29201. |
| 104 | 91 | Berry2021 | Berry, A., et al. (2021). Examining the feasibility, acceptability, validity and reliability of physical activity, sedentary behaviour and sleep measures in people with schizophrenia. Mental Health and Physical Activity 21: 100415. |
| 84 | 92 | Berry2017 | Berry, N., et al. (2017). Use of the Internet and Mobile Phones for Self-Management of Severe Mental Health Problems: Qualitative Study of Staff Views. JMIR Mental Health 4(4). |
| 33 | 93 | Bucci2019b | Bucci, S., et al. (2019). They are not hard-to-reach clients. We have just got hard-to-reach services. Staff views of digital health tools in specialist mental health services. Frontiers in Psychiatry 10(MAY): 344. |
| 52 | 94 | Berry2019 | Berry, N., et al. (2019). A qualitative exploration of service user views about using digital health interventions for self-management in severe mental health problems. BMC Psychiatry 19(1): 35. |
| n/a | 95 | Biagianti2016 | Biagianti, B., et al. (2016). Creating Live Interactions to Mitigate Barriers (CLIMB): A Mobile Intervention to Improve Social Functioning in People With Chronic Psychotic Disorders. JMIR Mental Health 3(4). |
| 37 | 102 | Bonet2020 | Bonet, L., et al. (2020). ReMindCare App for Early Psychosis: Pragmatic Real World Intervention and Usability Study. JMIR mHealth and uHealth 8(11): e22997. |
| n/a | 104 | Hidalgo-Mazzei2016#3 | Bonnin, C. D., et al. (2021). Does cognitive impairment in bipolar disorder impact on a SIMPLe app use? Journal of Affective Disorders 282: 488-494. |
| 148 | 105 | Bopp2010 | Bopp, J. M., et al. (2010). The longitudinal course of bipolar disorder as revealed through weekly text messaging: a feasibility study. Bipolar Disord 12(3): 327-334. |
| 153 | 106 | Bowden2021 | Bowden, C. L., et al. (2021). Development of a Patient-Centered Software System to Facilitate Effective Management of Bipolar Disorder. Psychopharmacology bulletin 51(2): 8-19. |
| 48 | 107 | Nicholas2017 | Nicholas, J., et al. (2017). Beyond symptom monitoring: Consumer needs for bipolar disorder self-management using smartphones. European Psychiatry 44: 210-216. |
| 92 | 112 | Bos2020 | Bos, et al. (2020). Recommendations for the use of long-term experience sampling in bipolar disorder care: a qualitative study of patient and clinician experiences. International Journal of Bipolar Disorders 8(1): 38. |
| 124 | 117 | Brunette2019 | Brunette, M. F., et al. (2019). Pilot study of a mobile smoking cessation intervention for low-income smokers with serious mental illness. Journal of Smoking Cessation 14(4): 203-210. |
| 41 | 120 | Bucci2018b | Bucci, S., et al. (2018). Early Psychosis Service User Views on Digital Technology: Qualitative Analysis. JMIR Mental Health 5(4). |
| 139 | 121 | Bucci2019a#1 | Bucci, S., et al. (2019). A Theory-Informed Digital Health Intervention in People with Severe Mental Health Problems. Studies in health technology and informatics 264: 526-530. |
| n/a | 122 | Ben-Zeev2018b#2 | Buck, B., et al. (2020). Clinical and demographic predictors of engagement in mobile health vs. clinic-based interventions for serious mental illness. Journal of Behavioral and Cognitive Therapy 30(1): 3-11. |
| 114 | 126 | Camacho2021 | Camacho, E. and J. Torous (2021). Interest and readiness for digital mental health in coordinate specialty care for early course psychosis: A survey study of 42 programs in 30 states. Early Intervention in Psychiatry 15(5): 1243-1255. |
| 97 | 128 | Ben-Zeev2018b#3 | Carpenter-Song, E., et al. (2020). Perspectives on mobile health versus clinic-based group interventions for people with serious mental illnesses: A qualitative study. Psychiatric Services 71(1): 49-56. |
| n/a | 130 | Depp2015#1 | Depp, C. A. et al. (2015). Augmenting psychoeducation with a mobile intervention for bipolar disorder: A randomized controlled trial. Journal of Affective Disorders 174: 23-30. |
| 118 | 135 | Kreyenbuhl2019 | Kreyenbuhl, et al. (2019). Development and Feasibility Testing of a Smartphone Intervention to Improve Adherence to Antipsychotic Medications. Clinical Schizophrenia and Related Psychoses 13(1): 152-167. |
| 143 | 137 | Cho2020 | Cho, C.-H., et al. (2020). Effectiveness of a smartphone app with a wearable activity tracker in preventing the recurrence of mood disorders: Prospective case-control study. JMIR Mental Health 7(8): e21283. |
| 81 | 154 | Daus2018 | Daus, H., et al. (2018). Disease management apps and technical assistance systems for bipolar disorder: Investigating the patients' point of view. Journal of Affective Disorders 229: 351-357. |
| 53 | 156 | deAlmeida2019 | de Almeida, R. F. S., et al. (2019). Development of weCope, a mobile app for illness self-management in schizophrenia. ARCHIVES OF CLINICAL PSYCHIATRY 46(1): 1-4. |
| 67 | 160 | Depp2015#2 | Depp, C. A., et al. (2010). Mobile interventions for severe mental illness: design and preliminary data from three approaches. J Nerv Ment Dis 198(10): 715-721. |
| 44 | 162 | Lobban2017#2 | Dodd, A. L., et al. (2017). Users' experiences of an online intervention for bipolar disorder: important lessons for design and evaluation. Evidence-based mental health 20(4): 133-139. |
| 64 | 168 | Eisner2019#2 | Eisner, E., et al. (2019). Development and Long-Term Acceptability of ExPRESS, a Mobile Phone App to Monitor Basic Symptoms and Early Signs of Psychosis Relapse. JMIR mHealth and uHealth 7(3): e11568. |
| 126 | 169 | Eisner2019#1 | Eisner, E., et al. (2019). Feasibility of using a smartphone app to assess early signs, basic symptoms and psychotic symptoms over six months: A preliminary report. Schizophrenia Research 208: 105-113. |
| 119 | 175 | Enrique2020 | Enrique, A., et al. (2020). An internet-delivered self-management programme for bipolar disorder in mental health services in Ireland: Results and learnings from a feasibility trial. Clinical psychology & psychotherapy 27(6): 925-939. |
| 128 | 176 | Lopez-Morinigo2021 | Lopez-Morinigo J, et al. (2021). Use of ecological momentary assessment through a passive smartphone-based app (eB2) by patients with schizophrenia: Acceptability study. Journal of Medical Internet Research 23(7): e26548. |
| n/a | 181 | Faurholt-Jepsen2019 | Faurholt-Jepsen, M., et al. (2019). Smartphone-based self-monitoring in bipolar disorder: evaluation of usability and feasibility of two systems. International Journal of Bipolar Disorders 7(1): 1. |
| 127 | 182 | Faurholt-Jepsen2020 | Faurholt-Jepsen, M., et al. (2020). Validity and characteristics of patient-evaluated adherence to medication via smartphones in patients with bipolar disorder: exploratory reanalyses on pooled data from the MONARCA I and II trials. Evidence-based mental health 23(1): 2-7. |
| n/a | 184 | Fellendorf2021 | Fellendorf, F. T., et al. (2021). Monitoring Sleep Changes via a Smartphone App in Bipolar Disorder: Practical Issues and Validation of a Potential Diagnostic Tool. Frontiers in Psychiatry 12: 641241. |
| 152 | 188 | Ferron2012 | Ferron, J. C., et al. (2012). Do symptoms and cognitive problems affect the use and efficacy of a web-based decision support system for smokers with serious mental illness? Journal of Dual Diagnosis 8(4): 315-325. |
| 73 | 191 | Fletcher2021 | Fletcher, K. and G. Murray (2021). Towards tailored psychosocial intervention for BD-II: Lived experience perspectives on current and future management options. Journal of Affective Disorders 289: 110-116. |
| 87 | 192 | Thomas2016#1 | Thomas, et al. (2016). Promoting personal recovery in people with persisting psychotic disorders: Development and pilot study of a novel digital intervention. Frontiers in Psychiatry 7(DEC): 196. |
| 101 | 193 | Forchuk2015#1 | Forchuk, C., et al. (2015). Client perceptions of the mental health engagement network: a qualitative analysis of an electronic personal health record. BMC Psychiatry 15: 250. |
| 120 | 194 | Forchuk2015#2 | Forchuk, C., et al. (2015). Client Perceptions of the Mental Health Engagement Network: A Secondary Analysis of an Intervention Using Smartphones and Desktop Devices for Individuals Experiencing Mood or Psychotic Disorders in Canada. JMIR Mental Health 2(1). |
| 98 | 205 | Moura2019 | Moura., et al. (2019). Facilitating the Delivery of Cognitive Remediation in First-Episode Psychosis: Pilot Study of a Home-Delivered Web-Based Intervention. Journal of Nervous and Mental Disease 207(11): 951-957. |
| n/a | 206 | Fulford2020 | Fulford, D., et al. (2020). Development of the Motivation and Skills Support (MASS) social goal attainment smartphone app for (and with) people with schizophrenia. Journal of Behavioral and Cognitive Therapy 30(1): 23-32. |
| n/a | 212 | Whiteman2017 | Whiteman et al. (2017). Adapting a Psychosocial Intervention for Smartphone Delivery to Middle-Aged and Older Adults with Serious Mental Illness. American Journal of Geriatric Psychiatry 25(8): 819-828. |
| n/a | 220 | Gorczynski2014 | Gorczynski, P., et al. (2014). Examining strategies to improve accelerometer compliance for individuals living with schizophrenia. Psychiatric rehabilitation journal 37(4): 333-335. |
| 131 | 221 | Gordon-Smith2019 | Gordon-Smith, K., et al. (2019). Large-scale roll out of electronic longitudinal mood-monitoring for research in affective disorders: Report from the UK bipolar disorder research network. Journal of Affective Disorders 246: 789-793. |
| n/a | 225 | Granholm2012 | Granholm, E., et al. (2012). Mobile Assessment and Treatment for Schizophrenia (MATS): a pilot trial of an interactive text-messaging intervention for medication adherence, socialization, and auditory hallucinations. Schizophr Bull 38(3): 414-425. |
| 144 | 233 | Hanssen2020#1 | Hanssen, E., et al. (2020). An ecological momentary intervention incorporating personalised feedback to improve symptoms and social functioning in schizophrenia spectrum disorders. Psychiatry Research 284: 112695. |
| n/a | 234 | Rotondi2021 | Rotondi (2021). Key variables for effective ehealth designs for individuals with and without mental health disorders: 2 12-4 Fractional factorial experiment. Journal of Medical Internet Research 23(3): e23137. |
| 89 | 235 | Hardy2018 | Hardy, A., et al. (2018). How Inclusive, User-Centered Design Research Can Improve Psychological Therapies for Psychosis: Development of SlowMo. JMIR Mental Health 5(4). |
| 138 | 236 | Harris2017 | Harris, A. W. F., et al. (2017). Web-Based Cognitive Remediation Improves Supported Employment Outcomes in Severe Mental Illness: Randomized Controlled Trial. JMIR Mental Health 4(3). |
| 83 | 238 | Hatch2018 | Hatch, A., et al. (2018). Expert Consensus Survey on Digital Health Tools for Patients With Serious Mental Illness: Optimizing for User Characteristics and User Support. JMIR Mental Health 5(2). |
| 146 | 243 | Hidalgo-Mazzei2016#1 | Hidalgo-Mazzei, D., et al. (2016). Psychoeducation in bipolar disorder with a SIMPLe smartphone application: Feasibility, acceptability and satisfaction. Journal of Affective Disorders 200: 58-66. |
| 125 | 244 | Hidalgo-Mazzei2016#2 | Hidalgo-Mazzei, D., et al. (2017). Is a SIMPLe smartphone application capable of improving biological rhythms in bipolar disorder? J Affect Disord 223: 10-16. |
| 39 | 245 | Hidalgo-Mazzei2018 | Hidalgo-Mazzei, D., et al. (2018). OpenSIMPLe: A real-world implementation feasibility study of a smartphone-based psychoeducation programme for bipolar disorder. J Affect Disord 241: 436-445. |
| 58 | 250 | Huerta-Ramos2016#1 | Huerta-Ramos, E., et al. (2016). Measuring Users' Receptivity Toward an Integral Intervention Model Based on mHealth Solutions for Patients With Treatment-Resistant Schizophrenia (m-RESIST): A Qualitative Study. JMIR mHealth and uHealth 4(3). |
| 59 | 251 | Huerta-Ramos2016#2 | Huerta-Ramos, E., et al. (2017). m-RESIST, a complete m-Health solution for patients with treatment-resistant schizophrenia: A qualitative study of user needs and acceptability in the Barcelona metropolitan area. Actas espanolas de psiquiatria 45(6): 277-289. |
| n/a | 257 | Jonathan2021 | Jonathan, G. K., et al. (2021). A smartphone-based self-management intervention for bipolar disorder (livewell): User-centered development approach. JMIR Mental Health 8(4): e20424. |
| 63 | 258 | Ben-Zeev2018b#4 | Jonathan, G., et al. (2019). Life with FOCUS: A qualitative evaluation of the impact of a smartphone intervention on people with serious mental illness. Psychiatric rehabilitation journal 42(2): 182-189. |
| n/a | 262 | Depp2021 | Depp , et al. (2021). Ecological momentary facial emotion recognition in psychotic disorders. Psychological medicine: 1-9. |
| n/a | 264 | Kane2013 | Kane, J. M., et al. (2013). First experience with a wireless system incorporating physiologic assessments and direct confirmation of digital tablet ingestions in ambulatory patients with schizophrenia or bipolar disorder. Journal of Clinical Psychiatry 74(6): e533-e540. |
| n/a | 265 | Välimäki2017#2 | Kannisto, K. A., et al. (2017). Factors Associated With Dropout During Recruitment and Follow-Up Periods of a mHealth-Based Randomized Controlled Trial for Mobile.Net to Encourage Treatment Adherence for People With Serious Mental Health Problems. Journal of Medical Internet Research 19(2): e46. |
| 109 | 271 | Kidd2019 | Kidd, S. A., et al. (2019). Feasibility and outcomes of a multi-function mobile health approach for the schizophrenia spectrum: APP4Independence (A4I). PLoS ONE 14(7): e0219491. |
| n/a | 284 | Kopelowicz2017 | Kopelowicz, A., et al. (2017). A multicenter, open-label, pilot study evaluating the functionality of an integrated call center for a digital medicine system to optimize monitoring of adherence to oral aripiprazole in adult patients with serious mental illness. Neuropsychiatric Disease and Treatment 13: 2641-2651. |
| 108 | 291 | Laine2019 | Laine, A., et al. (2019). Feasibility, Acceptability, and Preliminary Impacts of Web-Based Patient Education on Patients With Schizophrenia Spectrum Disorder: Quasi-Experimental Cluster Study. Journal of Medical Internet Research 21(10): e13073. |
| 60 | 292 | Lal2015 | Lal, S., et al. (2015). Preferences of Young Adults With First-Episode Psychosis for Receiving Specialized Mental Health Services Using Technology: A Survey Study. JMIR Mental Health 2(2). |
| 55 | 298 | Lederman2014 | Lederman, R., et al. (2014). Moderated online social therapy: Designing and evaluating technology for mental health. ACM Transactions on Computer-Human Interaction 21(1): 1-26. |
| 110 | 302 | Lewandowski2021 | Lewandowski, K. E. (2021). Feasibility and tolerability of a cognitive remediation clinical service in first episode coordinated specialty care. Early Intervention in Psychiatry 15(2): 391-396. |
| 111 | 306 | Xu2019#2 | Cai et al. (2020). Mobile texting and lay health supporters to improve schizophrenia care in a resource-poor community in rural China (LEAN Trial): Randomized controlled trial extended implementation. Journal of Medical Internet Research 22(12): e22631. |
| 142 | 311 | Lim2020 | Lim et al. (2020). A pilot digital intervention targeting loneliness in young people with psychosis. Social psychiatry and psychiatric epidemiology 55(7): 877-889. |
| 61 | 315 | Ludwig2021 | Ludwig, K. A., et al. (2021). Horyzons USA: A moderated online social intervention for first episode psychosis. Early Intervention in Psychiatry 15(2): 335-343. |
| n/a | 316 | Rotondi2012 | Rotondi et al. (2012). Use of a fractional factorial experiment to assess the e-healthcare application design needs of persons with dual diagnosis. Journal of Dual Diagnosis 8(4): 277-282. |
| 130 | 317 | Luther2020 | Luther, L., et al. (2020). Mobile Enhancement of Motivation in Schizophrenia: A Pilot Randomized Controlled Trial of a Personalized Text Message Intervention for Motivation Deficits. Journal of Consulting and Clinical Psychology. |
| 82 | 323 | Matthews2017 | Matthews, M., et al. (2017). Quantifying the changeable self: The role of self-tracking in coming to terms with and managing bipolar disorder. Human-Computer Interaction 32(5-6): 413-446. |
| 115 | 326 | McClelland2018 | McClelland, G. T. and M. Fitzgerald (2018). A participatory mobile application (app) development project with mental health service users and clinicians. Health Education Journal 77(7): 815-827. |
| 149 | 329 | McKnight2017 | McKnight, R. F., et al. (2017). Longitudinal mood monitoring in bipolar disorder: Course of illness as revealed through a short messaging service. Journal of Affective Disorders 223: 139-145. |
| 62 | 330 | Medalia2021 | Medalia, A., et al. (2021). Feasibility and acceptability of remotely accessed cognitive remediation for schizophrenia in public health settings. Psychiatry Research 301: 113956. |
| n/a | 331 | Medenblik2020 | Medenblik, A. M., et al. (2020). Treatment Outcomes of a Multi-Component Mobile Health Smoking Cessation Pilot Intervention for People with Schizophrenia. Journal of Dual Diagnosis 16(4): 420-428. |
| n/a | 333 | Merzenich2014 | Merzenich, M. M., et al. (2014). A novel, online social cognitive training program for young adults with schizophrenia: A pilot study. Schizophrenia Research: Cognition 1(1): e11-e19. |
| 145 | 335 | Meyer2018 | Meyer, N., et al. (2018). Capturing Rest-Activity Profiles in Schizophrenia Using Wearable and Mobile Technologies: Development, Implementation, Feasibility, and Acceptability of a Remote Monitoring Platform. JMIR mHealth and uHealth 6(10). |
| n/a | 338 | Miller2015 | Miller, B. J., et al. (2015). How connected are people with schizophrenia? Cell phone, computer, email, and social media use. Psychiatry Research 225(3): 458-463. |
| 116 | 340 | Mistler2017 | Mistler, L. A., et al. (2017). Mobile Mindfulness Intervention on an Acute Psychiatric Unit: Feasibility and Acceptability Study. JMIR Mental Health 4(3). |
| 99 | 341 | Moitra2017 | Moitra, E., et al. (2017). Feasibility and acceptability of post-hospitalization ecological momentary assessment in patients with psychotic-spectrum disorders. Comprehensive Psychiatry 74: 204-213. |
| n/a | 342 | Moitra2021 | Moitra, E., et al. (2021). Development and Initial Testing of an mHealth Transitions of Care Intervention for Adults with Schizophrenia-Spectrum Disorders Immediately Following a Psychiatric Hospitalization. Psychiatric Quarterly 92(1): 259-272. |
| 90 | 348 | Michalak2019#2 | Morton, E., et al. (2019). Experiences of a Web-Based Quality of Life Self-Monitoring Tool for Individuals With Bipolar Disorder: A Qualitative Exploration. JMIR Mental Health 6(12). |
| 47 | 349 | Morton2021b | Morton, E., et al. (2021). Use of smartphone apps in bipolar disorder: An international web-based survey of feature preferences and privacy concerns. Journal of Affective Disorders 295: 1102-1109. |
| 49 | 350 | Morton2021a | Morton, E., et al. (2021). Using apps for bipolar disorder - An online survey of healthcare provider perspectives and practices. Journal of Psychiatric Research 137: 22-28. |
| 93 | 351 | Mueller2018 | Mueller, N. E., et al. (2018). Using Smartphone Apps to Promote Psychiatric Rehabilitation in a Peer-Led Community Support Program: Pilot Study. JMIR Mental Health 5(3). |
| 69 | 353 | Murnane2016 | Murnane, E. L., et al. (2016). Self-monitoring practices, attitudes, and needs of individuals with bipolar disorder: Implications for the design of technologies to manage mental health. Journal of the American Medical Informatics Association 23(3): 477-484. |
| n/a | 355 | Murray2015 | Murray, G., et al. (2015). Online mindfulness-based intervention for late-stage bipolar disorder: Pilot evidence for feasibility and effectiveness. Journal of Affective Disorders 178: 46-51. |
| 57 | 358 | Naslund2016#2 | Naslund, J. A., et al. (2016). Feasibility and acceptability of Facebook for health promotion among people with serious mental illness. DIGITAL HEALTH 2. |
| 105 | 361 | Naslund2016#1 | Naslund, J. A., et al. (2016). Wearable devices and smartphones for activity tracking among people with serious mental illness. Mental Health and Physical Activity 10: 10-17. |
| 121 | 362 | Proudfoot2012#2 | Nicholas, J., et al. (2010). The ins and outs of an online bipolar education program: a study of program attrition. Journal of Medical Internet Research 12(5): e57. |
| n/a | 364 | Niendam2018 | Niendam, T. A., et al. (2018). Enhancing early psychosis treatment using smartphone technology: A longitudinal feasibility and validity study. Journal of Psychiatric Research 96: 239-246. |
| n/a | 366 | Aref-Adib2016 | Aref-Adib, et al. (2016). A qualitative study of online mental health information seeking behaviour by those with psychosis. BMC Psychiatry 16(1): 232. |
| 79 | 369 | Röhricht2021 | Röhricht et al. (2021). Simple Mobile technology health management tool for people with severe mental illness: a randomised controlled feasibility trial. BMC Psychiatry 21(1): 357. |
| n/a | 370 | Palmier-Claus2012 | Palmier-Claus, J. E., et al. (2012). The feasibility and validity of ambulatory self-report of psychotic symptoms using a smartphone software application. BMC Psychiatry 12: 172. |
| 91 | 374 | Peters-Strickland2016 | Peters-Strickland, T., et al. (2016). Usability of a novel digital medicine system in adults with schizophrenia treated with sensor-embedded tablets of aripiprazole. Neuropsychiatric Disease and Treatment 12: 2587-2594. |
| 80 | 375 | Ybarra2019 | Ybarra, M. L., et al. (2019). Developing Texting for Relapse Prevention: A Scalable mHealth Program for People With Schizophrenia and Schizoaffective Disorder. J Nerv Ment Dis 207(10): 854-862. |
| 72 | 377 | Poole2012 | Poole, R., et al. (2012). Internet-based psychoeducation for bipolar disorder: A qualitative analysis of feasibility, acceptability and impact. BMC Psychiatry 12(1): 139. |
| 103 | 380 | Prociow2012 | Prociow, P., et al. (2012). Mobile psychiatry: towards improving the care for bipolar disorder. International Journal of Mental Health Systems 6: 5. |
| 134 | 381 | Proudfoot2012#1 | Proudfoot, J., et al. (2012). Effects of adjunctive peer support on perceptions of illness control and understanding in an online psychoeducation program for bipolar disorder: A randomised controlled trial. Journal of Affective Disorders 142(1-3): 98-105. |
| 74 | 382 | Puskar2011 | Puskar, K., et al. (2011). Relational agents as an adjunct in Schizophrenia treatment. Journal of Psychosocial Nursing and Mental Health Services 49(8): 22-29. |
| n/a | 384 | Raugh2021 | Raugh, I. M., et al. (2021). Digital phenotyping adherence, feasibility, and tolerability in outpatients with schizophrenia. J Psychiatr Res 138: 436-443. |
| n/a | 386 | Raymaker2020 | Raymaker, D. M., et al. (2020). Early Assessment and Support Alliance Connections: Community-Based Participatory Research to Develop a Peer-based Early Psychosis Web Resource with Young Adults. Progress in community health partnerships : research, education, and action 14(4): 471-480. |
| 70 | 387 | Realpe2020 | Realpe, A., et al. (2020). Co-designing a virtual world with young people to deliver social cognition therapy in early psychosis. Early Intervention in Psychiatry 14(1): 37-43. |
| n/a | 390 | Roberts2017 | Roberts, D. L., et al. (2017). A tablet-based intervention to manipulate social cognitive bias in schizophrenia. American Journal of Psychiatric Rehabilitation 20(2): 143-155. |
| 106 | 393 | Rodriguez-Villa2021 | Rodriguez-Villa, E., et al. (2021). Cross cultural and global uses of a digital mental health app: results of focus groups with clinicians, patients and family members in India and the United States. GLOBAL MENTAL HEALTH 8. |
| n/a | 397 | Hatch2017 | Hatch, A., et al. (2017). Expert consensus survey on medication adherence in psychiatric patients and use of a digital medicine system. Journal of Clinical Psychiatry 78(7): e803-e812. |
| n/a | 398 | Rotondi2015 | Rotondi, A. J., et al. (2015). Critical Design Elements of E-Health Applications for Users with Severe Mental Illness: Singular Focus, Simple Architecture, Prominent Contents, Explicit Navigation, and Inclusive Hyperlinks. Schizophrenia Bulletin 41(2): 440-448. |
| n/a | 401 | Sablier2012 | Sablier, J., et al. (2012). Ecological assessments of activities of daily living and personal experiences with Mobus, an assistive technology for cognition: a pilot study in schizophrenia. Assistive technology : the official journal of RESNA 24(2): 67-77. |
| 66 | 404 | Saunders2017 | Saunders, K. E., et al. (2017). Experiences of remote mood and activity monitoring in bipolar disorder: A qualitative study. Eur Psychiatry 41: 115-121. |
| n/a | 407 | Schlosser2016 | Schlosser, D., et al. (2016). Feasibility of PRIME: A Cognitive Neuroscience-Informed Mobile App Intervention to Enhance Motivated Behavior and Improve Quality of Life in Recent Onset Schizophrenia. JMIR RESEARCH PROTOCOLS 5(2). |
| n/a | 412 | Sedgwick2021 | Sedgwick, O., et al. (2021). I wanted to do more of the homework!-Feasibility and acceptability of blending app-based homework with group therapy for social cognition in psychosis. Journal of clinical psychology. |
| 135 | 418 | Simon2011 | Simon, G. E., et al. (2011). An online recovery plan program: Can peer coaching increase participation? Psychiatric Services 62(6): 666-669. |
| n/a | 427 | Sreejith2019 | Sreejith, G. and V. Menon (2019). Mobile Phones as a Medium of Mental Health Care Service Delivery: Perspectives and Barriers among Patients with Severe Mental Illness. INDIAN JOURNAL OF PSYCHOLOGY MEDICINE 41(5): 428-433. |
| 141 | 429 | Steare2020#1 | Steare, T., et al. (2020). Smartphone-delivered self-management for first-episode psychosis: The ARIES feasibility randomised controlled trial. BMJ Open 10(8): e034927. |
| 34 | 430 | Steare2020#2 | Steare, T., et al. (2021). A qualitative study of stakeholder views on the use of a digital app for supported self-management in early intervention services for psychosis. BMC Psychiatry 21(1): 311. |
| n/a | 434 | Storm2021 | Storm, M., et al. (2021). Usability testing of a mobile health application for self-management of serious mental illness in a norwegian community mental health setting. International Journal of Environmental Research and Public Health 18(16): 8667. |
| 137 | 438 | Swartz2021 | Swartz, H. A., et al. (2021). A randomized pilot study of Rhythms And You (RAY): An internet-based program for bipolar disorder administered with and without clinical helper support in primary care. Journal of Affective Disorders 295: 183-191. |
| 78 | 440 | Switsers2018 | Switsers, L., et al. (2018). Users' Perspectives on mHealth Self-Management of Bipolar Disorder: Qualitative Focus Group Study. JMIR mHealth and uHealth 6(5). |
| 45 | 443 | Terp2018 | Terp, M., et al. (2018). A Smartphone App to Foster Power in the Everyday Management of Living With Schizophrenia: Qualitative Analysis of Young Adults' Perspectives. JMIR Mental Health 5(4). |
| 40 | 444 | Lal2018 | Lal, et al. (2018). Seeking mental health information and support online: experiences and perspectives of young people receiving treatment for first-episode psychosis. Early Intervention in Psychiatry 12(3): 324-330. |
| 150 | 445 | Thomas2017 | Thomas, N., et al. (2017). Are people with severe mental illness ready for online interventions? Access and use of the Internet in Australian mental health service users. Australasian Psychiatry 25(3): 257-261. |
| n/a | 446 | Wilson2019 | Wilson, et al. (2019). Patient-Informed Treatment Development of Behavioral Smoking Cessation for People With Schizophrenia. Behavior Therapy 50(2): 395-409. |
| 129 | 447 | Thonon2022 | Thonon, B., et al. (2022) A Group Intervention for Motivational Deficits: Preliminary Investigation of a Blended Care Approach Using Ambulatory Assessment. Behavior modification. |
| 122 | 450 | Todd2013 | Todd, N. J., et al. (2013). What do service users with bipolar disorder want from a web-based self-management intervention? A qualitative focus group study. Clinical psychology & psychotherapy 20(6): 531-543. |
| n/a | 452 | Tolley2015 | Tolley, C., et al. (2015). The feasibility of using electronic clinical outcome assessments in people with schizophrenia and their informal caregivers. PATIENT-RELATED OUTCOME MEASURES 6: 91-101. |
| n/a | 453 | Torous2017 | Torous, J. and S. Roux (2017). Patient-Driven Innovation for Mobile Mental Health Technology: Case Report of Symptom Tracking in Schizophrenia. JMIR Mental Health 4(3). |
| 155 | 455 | Torous2018 | Torous, J., et al. (2018). Mental Health Mobile Phone App Usage, Concerns, and Benefits Among Psychiatric Outpatients: Comparative Survey Study. JMIR Mental Health 5(4). |
| n/a | 463 | Vaessen2019 | Vaessen, T., et al. (2019). ACT in daily life in early psychosis: An ecological momentary intervention approach. Psychosis: Psychological, Social and Integrative Approaches 11(2): 93-104. |
| n/a | 464 | Vaidyam2020 | Vaidyam, A., et al. (2020). Patient innovation in investigating the effects of environmental pollution in schizophrenia: Case report of digital phenotyping beyond apps. JMIR Mental Health 7(8): e19778. |
| 68 | 468 | Valentine2020 | Valentine, L., et al. (2020). Blended digital and face-to-face care for first-episode psychosis treatment in young people: Qualitative study. JMIR Mental Health 7(7): e18990. |
| 65 | 469 | Alvarez-Jimenez2021#3 | Valentine, L., et al. (2021). Young people's experience of online therapy for first-episode psychosis: A qualitative study. Psychology and psychotherapy. |
| 112 | 472 | Välimäki2017#1 | Valimaki, M., et al. (2017). Short Text Messages to Encourage Adherence to Medication and Follow-up for People With Psychosis (Mobile.Net): Randomized Controlled Trial in Finland. Journal of Medical Internet Research 19(7): e245. |
| 88 | 474 | vandenHeuvel2018 | van den Heuvel, S. C. G. H., et al. (2018). The user experiences and clinical outcomes of an online personal health record to support self-management of bipolar disorder: A pretest-posttest pilot study. Journal of Affective Disorders 238: 261-268. |
| n/a | 475 | vanderKrieke2012 | van der Krieke, L., et al. (2012). Usability evaluation of a web-based support system for people with a schizophrenia diagnosis. Journal of Medical Internet Research 14(1): e24. |
| 136 | 478 | VanTil2020 | Van Til, K., et al. (2020). A comparative study of engagement in mobile and wearable health monitoring for bipolar disorder. Bipolar Disorders 22(2): 182-190. |
| 77 | 483 | Vilardaga2019 | Vilardaga, R., et al. (2019). Formative, multimethod case studies of learn to quit, an acceptance and commitment therapy smoking cessation app designed for people with serious mental illness. Translational Behavioral Medicine 9(6): 1076-1086. |
| 94 | 489 | Wenze2016 | Wenze, S. J., et al. (2016). An Open Trial of a Smartphone-assisted, Adjunctive Intervention to Improve Treatment Adherence in Bipolar Disorder. Journal of Psychiatric Practice 22(6): 492-504. |
| 35 | 491 | Williams2018 | Williams, A., et al. (2018). Going Online Together: The Potential for Mental Health Workers to Integrate Recovery Oriented E-Mental Health Resources Into Their Practice. Psychiatry (New York) 81(2): 116-129. |
| n/a | 492 | Thomas2016#2 | Williams, A., et al. (2018). Recovery After Psychosis: Qualitative Study of Service User Experiences of Lived Experience Videos on a Recovery-Oriented Website. JMIR Mental Health 5(2). |
| 117 | 493 | Hanssen2020#2 | Williams, A., et al. (2021). Impact of jointly using an e-Mental health resource (self-management and recovery technology) on interactions between service users experiencing severe mental illness and community mental health workers: Grounded theory study. JMIR Mental Health 8(6): e25998. |
| n/a | 497 | Brunette2020 | Brunette, M., et al. (2020). Brief, web-based interventions to motivate smokers with schizophrenia: Randomized controlled trial. JMIR Mental Health 7(2): e16524. |
| 113 | 498 | Xu2019#1 | Xu, D., et al. (2019). Lay health supporters aided by mobile text messaging to improve adherence, symptoms, and functioning among people with schizophrenia in a resource-poor community in rural China (LEAN): A randomized controlled trial. PLoS Medicine 16(4): e1002785. |

1. In most studies to date, VR interventions are only tested during in-person sessions with a clinician/researcher. Hence, most VR studies would be excluded by the previous criterion, leaving only an incomplete sub-set of such studies to be reviewed. Furthermore, VR interventions are rather different (e.g., more immersive/realistic) than digital tools delivered via other means (smartphone, website) and may therefore have quite distinct barriers/facilitators. In future, a separate review examining barriers/facilitators to user engagement with VR, specifically, may be valuable. [↑](#footnote-ref-2)
2. Ng, M. M., Firth, J., Minen, M., & Torous, J. (2019). User engagement in mental health apps: A review of measurement, reporting, and validity. Psychiatric Services, 70(7), 538–544. https://doi.org/10.1176/appi.ps.201800519 [↑](#footnote-ref-3)
